# Bimolecular Coupling in Olefin Metathesis: Correlating Structure and Decomposition for Leading and Emerging Ruthenium−Carbene Catalysts

**DOI:** 10.1021/jacs.1c04424

**Published:** 2021-07-16

**Authors:** Daniel L. Nascimento, Marco Foscato, Giovanni Occhipinti, Vidar R. Jensen, Deryn E. Fogg

**Affiliations:** † Center for Catalysis Research & Innovation, and Department of Chemistry and Biomolecular Sciences, University of Ottawa, Ottawa, Canada K1N 6N5; ‡ Department of Chemistry, University of Bergen, Allégaten 41, N-5007 Bergen, Norway

## Abstract

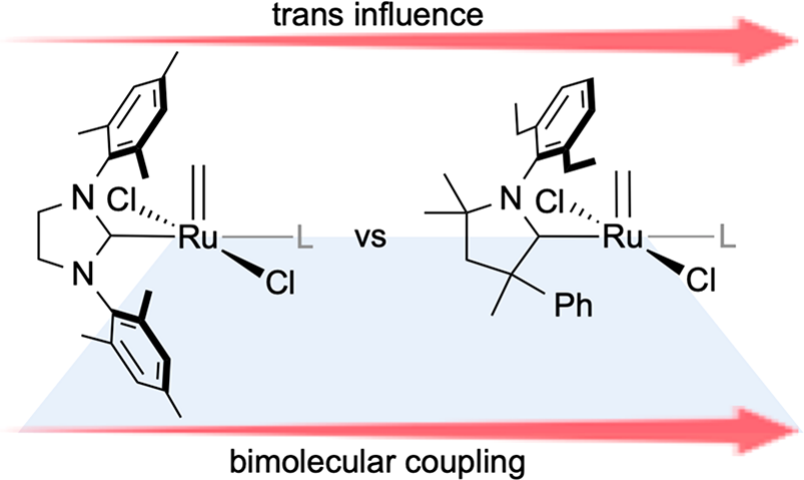

Bimolecular catalyst decomposition is a fundamental, long-standing challenge in olefin metathesis. Emerging ruthenium–cyclic(alkyl)(amino)carbene (CAAC) catalysts, which enable breakthrough advances in productivity and general robustness, are now known to be extraordinarily susceptible to this pathway. The details of the process, however, have hitherto been obscure. The present study provides the first detailed mechanistic insights into the steric and electronic factors that govern bimolecular decomposition. Described is a combined experimental and theoretical study that probes decomposition of the key active species, RuCl_2_(L)(py)(=CH_2_) **1** (in which L is the N-heterocyclic carbene (NHC) H_2_IMes, or a CAAC ligand: the latter vary in the NAr group (NMes, N-2,6-Et_2_C_6_H_3_, or N-2-Me,6-*^i^*PrC_6_H_3_) and the substituents on the quaternary site flanking the carbene carbon (i.e., CMe_2_ or CMePh)). The transiently stabilized pyridine adducts **1** were isolated by cryogenic synthesis of the metallacyclobutanes, addition of pyridine, and precipitation. All are shown to decompose via second-order kinetics at −10 °C. The most vulnerable CAAC species, however, decompose more than 1000-fold faster than the H_2_IMes analogue. Computational studies reveal that the key factor underlying accelerated decomposition of the CAAC derivatives is their stronger trans influence, which weakens the Ru−py bond and increases the transient concentration of the 14-electron methylidene species, RuCl_2_(L)(=CH_2_) **2**. Fast catalyst initiation, a major design goal in olefin metathesis, thus has the negative consequence of accelerating decomposition. Inhibiting bimolecular decomposition offers major opportunities to transform catalyst productivity and utility, and to realize the outstanding promise of olefin metathesis.

## Introduction

**Chart 1:**
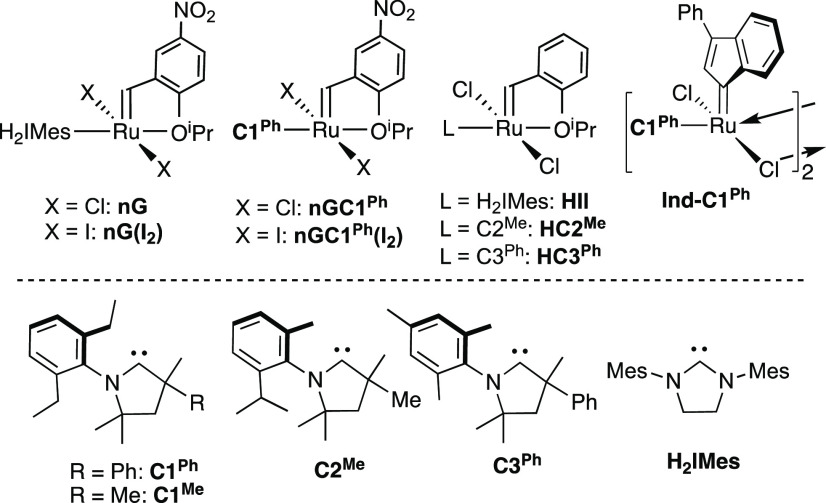
Catalysts and Carbene Ligands Discusseda

Olefin metathesis offers exceptional versatility in the catalytic assembly of carbon–carbon bonds.^1,2^ Recent advances hold great promise for overcoming productivity challenges in frontier applications, including pharmaceutical manufacturing,^3^ materials science,^4,5^ and chemical biology.^6^ Notwithstanding the groundbreaking impact of the dominant Ru–H_2_IMes catalysts, their facile decomposition is a fundamental limitation.^7^ Of major importance, therefore, is the breakthrough performance of cyclic (alkyl)(amino) carbene derivatives (CAAC; Chart 1).^8^ The CAAC catalysts show unprecedented productivity in the transformation of renewable fatty acids into α-olefins by cross-metathesis with ethylene (“ethenolysis”),^9−12^ as first reported by Bertrand and Grubbs in 2015,^10^ and in macrocyclization via ring-closing metathesis^11−13^ (mRCM). The latter process is of highly topical interest for the production of antiviral drugs.^3^

a The CAAC labeling system adopted (C#^R^) defines ligand families by common NAr moiety. The superscript R specifies the fourth substituent on the quaternary site flanking the carbene carbon.

**Scheme 1:**
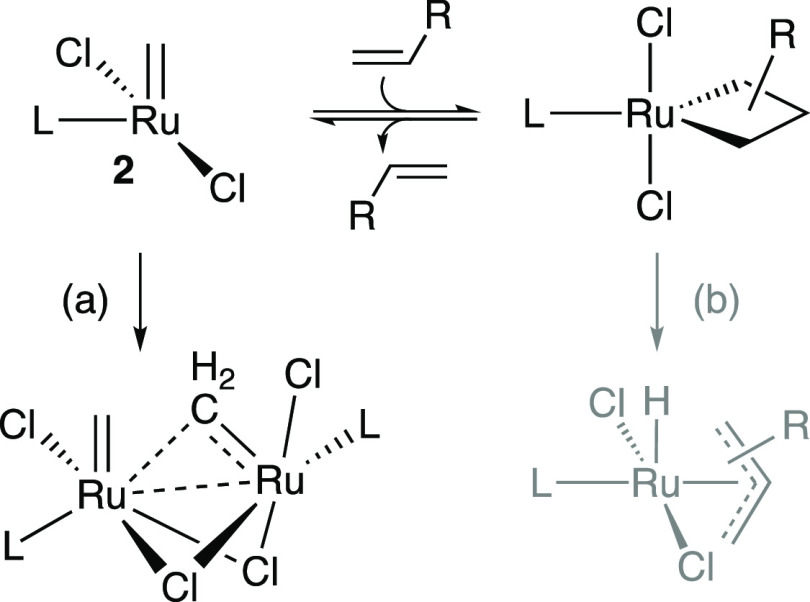
Intrinsic Decomposition Pathways: (a) Bimolecular Decomposition; (b) β-Hydride Elimination Path (b) was found to be negligible for L = **C1**^**Ph**^ and **C2**^**Me**^: see text.

Leading Ru–H_2_IMes catalysts were long thought to initiate too slowly to decompose via bimolecular coupling of methylidene species **2** (Scheme 1a).^14,15^ This is not the case: bimolecular decomposition is now known to compete with the general, well-established β-hydride elimination pathway^16,17^ shown in Scheme 1b.^18^ Indeed, we recently reported that the Ru-CAAC catalysts resist β-hydride elimination, but appear highly sensitive to bimolecular decomposition.^18a^ This would account for the sometimes striking drop in metathesis productivity evident when catalyst loadings are increased.^19^ In studies of transiently stabilized methylidene species, we demonstrated that bimolecular coupling is significantly faster for the CAAC catalyst **1-C1**^**Ph**^ than its H_2_IMes analogue **1-H**_**2**_**IMes**.^20^ To date, the factors that govern this pathway remain poorly understood. Although bimolecular coupling is a general vector for decomposition of both early and late transition methylidene species,^14−16,18^ many details remain obscure. Here we present an experimental and computational study that provides the first detailed insight into the process, and its sensitivity to the nature of the neutral carbene ligand. These findings are expected to aid both strategic planning and de novo catalyst design.^21,22^

The key experimental evidence for bimolecular coupling of RuCl_2_(L)(py)(=CH_2_) (L = H_2_IMes, **C1**^**Ph**^) in our prior work was the liberation of ethylene from the isolated pyridineadducts in ca. 80% yield.^18a,18b^ Essential for quantitation was rapid warming of the samples from −20 °C to rt, to minimize loss of ethylene to the headspace. In the present study, we sought to probe the relevant structure–decomposition relationships, by assessing the relative susceptibility to bimolecular coupling of the series of CAAC and H_2_IMes complexes shown in Chart 1. We began with a kinetics study of the isothermal decomposition of these transiently stabilized complexes at −10 °C.

## Results and Discussion

**Scheme 2:**
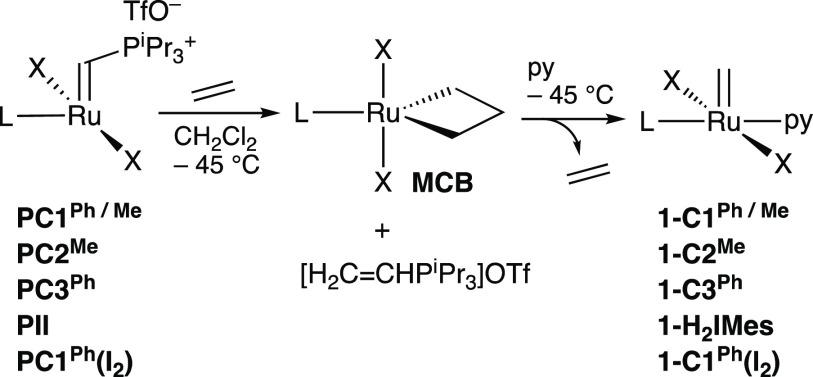
Synthesis of Transiently Stabilized Methylidene Complexes RuX_2_(L)(py)(=CH_2_), **1** L = **C1**^**Ph**^, **C1**^**Me**^, **C2**^**Me**^, **C3**^**Ph**^, H_2_IMes. X = Cl in all cases except RuI_2_(**C1**^**Ph**^)(py)(=CH_2_).

The methylidene species were synthesized via the cryogenic protocol of Scheme 2,^18a,18b^ in which the Piers phosphonium alkylidenes were treated with ethylene to form the metallacyclobutane **MCB**,^17a,23^ then with pyridine to collapse the ring and form the pyridine adducts **1**. The phosphonium ylide coproduct, [H_2_C=CHP^i^Pr_3_]OTf, was precipitated by cannula addition of cold (−110 °C) hexanes, and removed by filtration. Evaporation of the filtrate enabled isolation of the py adducts for all but **1-C2**^**Me**^. The latter was formed, as indicated by observation of the diagnostic ^1^H NMR signal for the [Ru]=C*H*_2_ protons at 18.22 ppm (Figure S18), but was too unstable to isolate.

**Figure 1:**
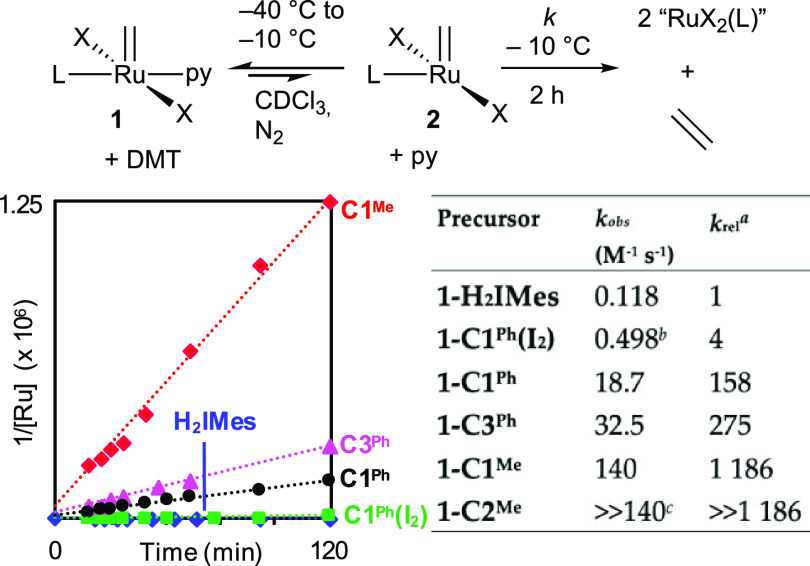
Second-order plot for bimolecular decomposition, and tabulated rate constants (*k*_obs_). Average of two trials.^24^^*a*^*k*_rel_ = rate constants normalized to that for the slowest-decomposing system, **1-H**_**2**_**IMes**. DMT = dimethyl terephthalate (internal standard). *^b^*A similar rate (0.444 M^–1^ s^–1^) was observed in C_7_D_8_. *^c^*A lower limit is given for **1-C2**^**Me**^, which decomposed too rapidly to isolate.

With this set of five methylidene complexes in hand, we undertook NMR studies to establish their relative susceptibility to bimolecular decomposition. Accordingly, each was redissolved at −35 °C in a solution of CDCl_3_ containing an integration standard of known concentration. The samples were warmed to −10 °C, and their rates of decomposition were monitored from the decline in the intensity of the methylidene signal relative to that for the internal standard. Second-order kinetics were observed (Figure 1), confirming that decomposition is dominated by bimolecular coupling. The second-order rate constants spanned 3 orders of magnitude, with coupling being slowest for **1-H**_**2**_**IMes** and ≫1200 times faster for **1-C2**^**Me**^. The lower limit for the latter is set by the rate for **1-C1**^**Me**^, the fastest-decomposing species for which a rate could be measured.

**Figure 2:**
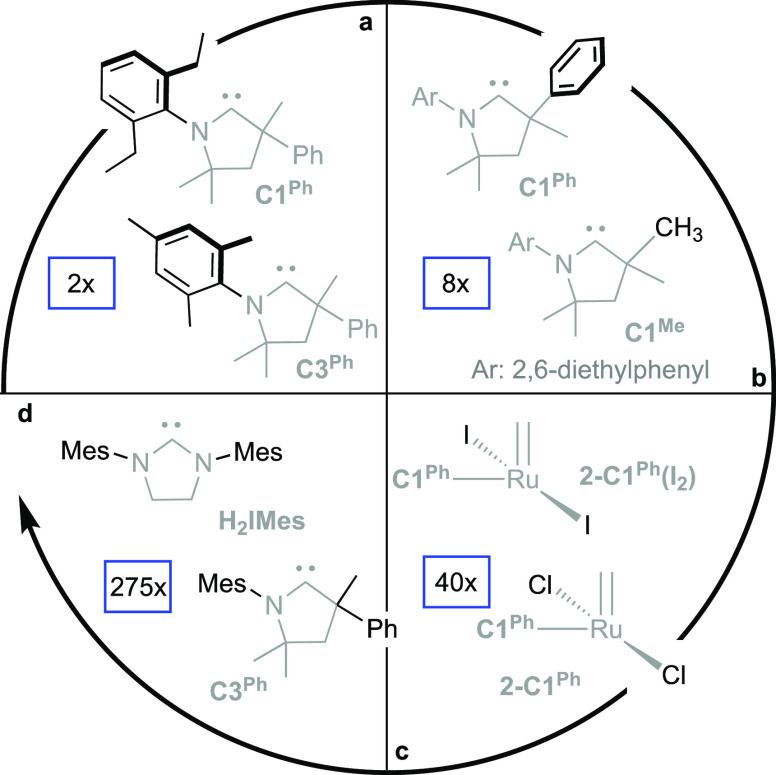
Relative rates (text in blue boxes) of bimolecular decomposition as a function of the structural changes shown in black: (a) NAr substituents. (b) Substitution at C_α_ (the quaternary center α to the carbene carbon). (c) The anionic ligand: chloride vs iodide. (d) NHC vs CAAC: H_2_IMes vs its closest analogue, **C3**^**Ph**^.

Figure 2 highlights the impact of individual structural features on rates of decomposition. We first consider the impact of the NAr *o*-aryl substituents, within CAAC ligands bearing a CMePh group adjacent to the carbene carbon (Figure 2a). The *N*-mesityl complex **1-C3**^**Ph**^ decomposes at twice the rate of its *N*-diethylphenyl (N-DEP) analogue **1-C1**^**Ph**^. That is, the rate of coupling is doubled by removing just one methylene unit from each *o*-substituent. (The mesityl *p*-methyl substituent in **C3**^**Ph**^ may also play a role, for example by increasing σ-donation slightly relative to **C1**^**Ph**^, but this effect is presumed to be minor.) Faster decomposition with diminishing NAr bulk would account for the lower productivity reported for multiple catalyst classes (including Hoveyda, Grela, and bis-CAAC platforms) when the **C1**^**Ph**^ ligand is replaced with **C3**^**Ph**^.^10,12,13^

Truncation of the quaternary CMePh group to CMe_2_ (**1-C1**^**Ph**^ vs **1-C1**^**Me**^; see Figure 2b) triggers both steric and electronic impacts. The N-DEP group is then too small to retard coupling, and **1-C1**^**Me**^ decomposes nearly 10× faster than **1-C1**^**Ph**^. Consistent with this trend are the lower turnover numbers reported for **C1**^**Me**^ catalysts relative to their **C1**^**Ph**^ analogues in multiple contexts, ranging from ethenolysis to acrylonitrile metathesis.^12,13,25^

Of note in this context is the much faster decomposition seen for **1-C2**^**Me**^, despite the presence of one relatively bulky *o*-*^i^*Pr substituent. Computational examination (see below) revealed that the latter in fact promotes pyridine loss to form the four-coordinate species **2-C2**^**Me**^, while being insufficient to impede coupling. The extreme sensitivity of the **C2**^**Me**^ catalysts to bimolecular decomposition is implied by multiple experimental studies, as we have noted elsewhere.^18,26^ Perhaps most striking is the negative impact of increased catalyst loadings on TONs for **HC2**^**Me**^ even at <5 ppm catalyst.^10,27^ Indeed, bimolecular coupling of **HC2**^**Me**^ appears to be so rapid at 70 °C that nucleophilic abstraction of the methylidene ligand is unable to compete, even when aggressive^28^ nucleophiles such as unencumbered primary amines are employed.^26^

An inherent trade-off is thus apparent between the steric protection required to retard bimolecular decomposition and the steric accessibility required for fast initiation and turnover. As illustrated in Figure 2c, replacing the chloride ligands in the **C1**^**Ph**^ derivative by iodide slows the rate of decomposition 40-fold. Iodide catalysts, long overlooked because of their lower reactivity,^29^ have recently been shown to offer productivity superior to their faster-initiating analogues in demanding contexts that require long catalyst lifetimes.^19b,30−33^ Retarded bimolecular decomposition is clearly an important component of this robustness, although it should be noted that coupling remains operative for **nG(I**_**2**_**)** even at micromolar catalyst concentrations.^19b^ Slowly initiating CAAC-iodide metathesis catalysts may thus be of keen interest for metathesis of accessible olefinic bonds, although few such complexes have yet been developed.^8a,33^

We come last to a more difficult comparison (Figure 2d), between **1-H**_**2**_**IMes** and its closest CAAC analogue, **1-C3**^**Ph**^. The superficially minor replacement of one H_2_IMes N-mesityl group by a CMePh unit dramatically increases the rate constant for decomposition, by 275×. Multiple parameters are affected by the transformation of an NHC to even a closely corresponding CAAC ligand, a point that has seen much recent discussion.^8a,34−37^ To probe the specific impact on bimolecular decomposition, we turned to computational analysis.

**Scheme 3:**
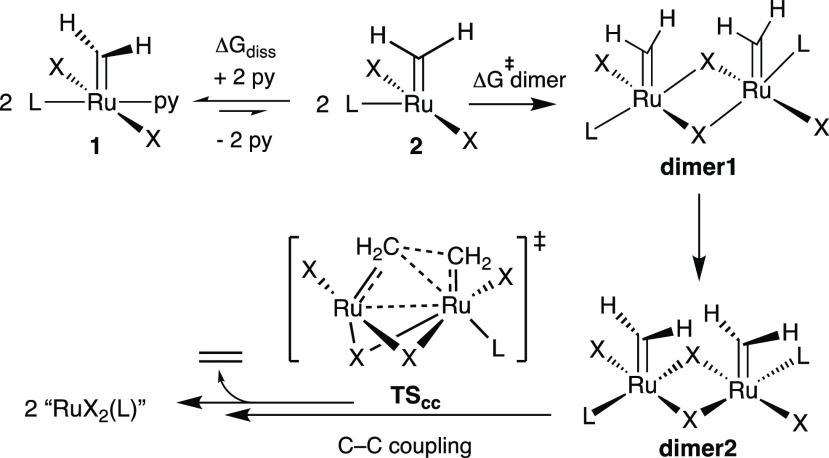
Key Steps in the Bimolecular Decomposition of **1** Identified by DFT Calculations

A density functional theory (DFT) analysis of the bimolecular coupling of **1-H**_**2**_**IMes** reveals a complex overall mechanism. Key intermediates and transition states are shown in Scheme 3, with details in the SI. Full exploration for the CAAC complexes is hampered by the multitude of isomers arising from the unsymmetrical nature of the carbene, and the chiral centers present in **C1**^**Ph**^ and **C3**^**Ph**^. We therefore limited study of the CAAC systems to the Ru species of Scheme 3, with diruthenium structures being further limited to the diastereomeric dimers and transition states of **1-C3**^**Ph**^. Even with these restrictions, the study included 16 unique structures for the C–C bond-forming transition state (**TS**_**CC**_) alone. The free energies in Table 1 were calculated using experimental catalyst concentrations: free energies calculated at 1 mM for all catalysts are provided in the Supporting Information (SI).

**Table 1:** Calculated Free Energies and Buried Volumes^a^

Starting Complex	Pyridine Loss (Δ*G*_diss_)	Dimerization (Δ*G*_dimer_^‡^)	Buried Volume (%*V*_bur_)^b^
**1-H**_**2**_**IMes**	7.6	19.5	81.9
**1-C1**^**Ph**^**(I**_**2**_**)**	4.4	13.2	88.6
**1-C1**^**Ph**^	3.9	12.3	83.7
**1-C3**^**Ph**^	3.8	12.1	82.6
**1-C1**^**Me**^	0.4	5.1	83.7
**1-C2**^**Me**^	–2.0	0.4	82.8

a Free energies in kcal/mol vs *G*(**1**), calculated for the most stable rotamers of **1** and **2** at experimental catalyst concentrations (**1-H**_**2**_**IMes**: 1.4 mM, **1-C1**^**Ph**^**(I**_**2**_**)**: 0.59 mM, **1-C1**^**Ph**^: 0.061 mM, **1-C3**^**Ph**^: 0.027 mM, **1-C1**^**Me**^: 0.01 mM). Δ*G*_diss_ = *G*(**2**) + *G*(py) – *G*(**1**); Δ*G*_dimer_^‡^ = 2 × Δ*G*_diss_ + Δ*G*_diff_^‡^ where Δ*G*_diff_^‡^ is the estimated lower limit for the free-energy barrier (4.4 kcal/mol). See SI for details.

b *%V*_bur_ = fraction of the first coordination sphere (radius 3.5 Å) that is occupied in **2**.^38^

**Figure 3:**
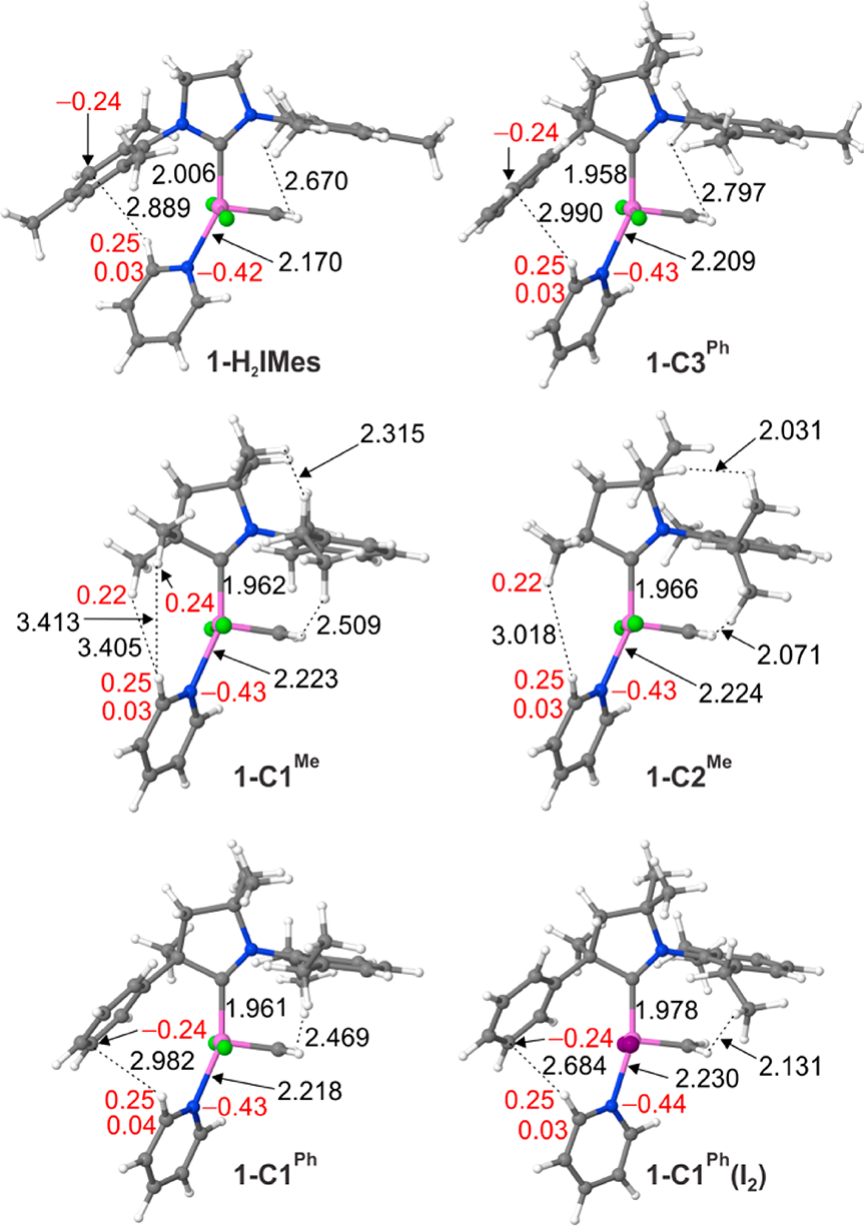
Selected atomic distances (Å) for py adducts **1** (DFT-optimized geometries). Ru: pink; Cl: green; I: violet; C: gray; N: blue; H: white). Natural charges (*e*) of selected atoms appear in red text.

The calculations suggest that bimolecular decomposition is controlled by a few key steps (Scheme 3). Even the initial ligand dissociation is important, as indicated by the inverse correlation between the rate constants for decomposition in Figure 1 and the free-energy changes for pyridine dissociation in Table 1. Thus, the highest penalty for loss of pyridine (Δ*G*_diss_ = 7.6 kcal/mol) is found for **1-H**_**2**_**IMes**, which is experimentally most resistant to bimolecular decomposition. Pyridine binding is ca. 3–10 kcal/mol weaker in the CAAC complexes, and the Ru–N bond distances are 3–6 pm longer (see Table 1 and DFT-optimized structures in Figure 3). The impact of this difference will be doubled in the relative decomposition rates, as two pyridine ligands must be lost for a single dimer to form.

Weakening of the Ru–py bonds in the CAAC complexes is due chiefly to the enhanced σ-donor and π-acceptor character of this carbene class,^8^ which increases the trans influence of the CAAC ligands relative to NHCs. In **1-C1**^**Ph**^**(I**_**2**_**)**, the most stable of the CAAC species studied, the trans influence of **C1**^**Ph**^ is attenuated by the Ru–C_carbene_ bond elongation induced by the bulky iodide ligands. The significant steric impact of the latter is evident from the much higher buried volume calculated for this complex (Table 1). The Ru–py bond in the iodide complex is hence 0.5 kcal/mol stronger than that in chloride analogue **1-C1**^**Ph**^, contributing to the reduced susceptibility to bimolecular decomposition.

A significantly weaker Ru–py bond is seen in **1-C1**^**Me**^ and (in particular) **1-C2**^**Me**^. Given the broad similarity in calculated buried volumes (%*V*_bur_; Table 1) for the various CAAC ligands,^39^ this instability is unlikely to be steric in origin. Rather, we suggest that the key feature that distinguishes **C1**^**Me**^ and **C2**^**Me**^ is the absence of an aromatic quaternary substituent that can participate in polar CH−π interactions^40,41^ with the pyridine ligand in **1**. In the most stable conformers of **1-C2**^**Me**^ and **1-C1**^**Me**^, the N-aryl group is syn to the methylidene, precluding such interaction. In the **C1**^**Ph**^ and **H**_**2**_**IMes** complexes, in comparison, an electron-rich aromatic ring is positioned to engage in hydrogen bonding and donor–acceptor bonding with the electron-deficient *o*-H and *o*-C pyridine atoms (natural charges = 0.25 *e* (H), 0.03–0.04 *e* (C); Figure 3).^42^

Importantly, these stabilizing interactions are not restricted to the pyridine ligand: they are likewise expected for bound olefin, owing to Ru-induced polarization of the sp^2^ C–H bonds. The consequent reduction in the concentration of the 14-electron species would limit bimolecular decomposition.^43^ For the CAAC catalysts to achieve these effects, however, a quaternary aromatic group is essential. In **1-C1**^**Me**^ and **1-C2**^**Me**^, the hydrogen atoms of the quaternary methyl groups bear a positive charge, as do the pyridine *o*-H and *o*-C atoms: this and the minimum Me–pyridine interatomic distances (>3 Å; Figure 3) reflect the absence of attractive interactions.

An additional factor affecting **1-C2**^**Me**^, beyond the absence of stabilizing polar CH−π interactions, is steric repulsion associated with the NAr *o*-isopropyl substituent. The latter is within ca. 2 Å of both the methylidene ligand and the methyl groups on the carbene backbone. Steric repulsion is relieved by pyridine dissociation and 90° rotation of the methylidene group to form **2**. The observed instability of **1-C2**^**Me**^ is thus due to a combination of steric and electronic factors.

The second-order kinetics evident in Figure 1 indicate that pyridine dissociation is not rate-limiting. Detailed calculations on **1-H**_**2**_**IMes** and **1-C3**^**Ph**^ instead suggest that the rate-determining step is coupling of two molecules of 14-electron **2** to form **dimer1** (Scheme 3), in which a chloride from each Ru atom serves as a dative ligand to the other Ru atom. Within this dimer, the geometry of the individual Ru centers in **2** is largely conserved, including the essentially orthogonal disposition of the methylidene ligand relative to the RuCl_2_ plane (Figures S20, S25). The minimal geometrical adaption needed for **2-H**_**2**_**IMes** and **2-C3**^**Ph**^ suggests little to no enthalpic cost to formation of **dimer1** from **2**. A lower bound for the barrier to dimerization can be obtained by assuming that the rate is diffusion-controlled. Rate constants for diffusion in common organic solvents are on the order of 4 × 10^9^ s^–1^,^44^ from which a barrier (Δ*G*_diff_^‡^) of 4.4 kcal/mol can be extracted using the Eyring equation. Summing this value and the free energies of two 14-electron complexes **2** gives an estimated overall barrier to dimerization Δ*G*_dimer_^‡^ of ca. 19.5 kcal/mol for **1-H**_**2**_**IMes** and 12.1 kcal/mol for **1-C3**^**Ph**^, relative to **1**.

In contrast, the ensuing rearrangement from **dimer1** to the more stable, tightly bonded **dimer2** is essentially barrierless. In **dimer2**, the methylidene groups return to a conformation aligned with the RuCl_2_ plane. All subsequent steps are facile compared to the initial dimerization. That is, the barrier to C–C bond formation via **TS**_**CC**_ is lower than that to formation of **dimer1** (Table S1), as is the subsequent formation of an ethylene-bridged Ru dimer, rearrangement to a η^2^-ethylene complex, and release of ethylene and Ru decomposition products (Figures S21, S22). The calculations for **1-H**_**2**_**IMes** and **1-C3**^**Ph**^ thus strongly suggest that the most energy-demanding step in bimolecular decomposition of the 14-electron complexes **2** is the formation of **dimer1**, rather than the ensuing coupling of methylidene units. Errors on the order of 2–5 kcal/mol for the calculated barriers Δ*G*_dimer_^‡^ are expected, given the general accuracy of DFT-calculated relative free energies (see the SI) and the exclusion of enthalpic contributions to dimerization of **2** discussed above. These translate to orders-of-magnitude variation in the rate constants, owing to the exponential (Eyring) relationship between barriers and rate constants. The agreement between the calculated dimerization barriers and the experimental rate constants should thus be expected to be qualitative only. Nevertheless, the computational prediction of the kinetic bottleneck is supported by the qualitative, rank-order agreement between the calculated barriers and the experimental rate constants, as well as the second-order kinetics (Figure S1), which support dimerization as the rate-determining step in the overall reaction.

## Conclusions

Bimolecular catalyst decomposition has long been recognized as a fundamental challenge in olefin metathesis. Leading ruthenium–carbene catalysts, initially thought to be immune, are now known to be extraordinarily susceptible, even at ppm catalyst loadings. The foregoing provides the first detailed mechanistic insights into the process, and the steric and electronic factors that govern decomposition. An experimental “catalyst susceptibility ranking” was established for the most productive CAAC and NHC catalysts, and qualitatively reproduced via DFT analysis, which revealed that dimerization of the 14-electron complex **2** is rate-determining. A major component of this barrier is ligand dissociation to generate **2**, dimerization of which is retarded surprisingly little even by relatively bulky carbene ligands. Fast catalyst initiation, aimed at rapid generation of metathesis-active **2**, is thus inextricably connected to accelerated bimolecular decomposition for state-of-the-art NHC and (particularly) CAAC catalysts. The striking susceptibility of the latter to bimolecular decomposition is shown to originate in the high trans influence of the CAAC ligand, which promotes formation of four-coordinate **2**. Very low catalyst concentrations are then necessary to restrict bimolecular decomposition. Inhibition of this major decomposition pathway offers major opportunities to transform catalyst productivity and scope, and to realize the outstanding promise of olefin metathesis.

## Supporting Information Available

Experimental details, NMR spectra, computational details and supplementary computational results and data (PDF)

## Notes

The computational data set is available from the ioChem-BD repository^45^ via 10.19061/iochem-bd-6-79.
