# Supplementary material for: Bimolecular Coupling in Olefin Metathesis: Correlating Structure and Decomposition for Leading and Emerging Ruthenium−Carbene Catalysts
Source: J Am Chem Soc. 2021 Jul 16;143(29):11072–9. doi: 10.1021/jacs.1c04424 (PMC8397316; doi:10.1021/jacs.1c04424)
Supplement: Supplementary file 1 — ja1c04424_si_001.pdf [file ja1c04424_si_001.pdf]

*Supporting Information*

# Bimolecular Coupling in Olefin Metathesis: Correlating Structure and Decomposition for Leading and Emerging Ruthenium-Carbene Catalysts

Daniel L. Nascimento,[a] Marco Foscato,[b] Giovanni Occhipinti,[b] Vidar R. Jensen[b]* and Deryn E. Fogg[a,b]*

[a]Center for Catalysis Research & Innovation, and Department of Chemistry and Biomolecular Sciences, University of Ottawa, Ottawa, Canada K1N 6N5. [b]Department of Chemistry, University of Bergen, Allégaten 41, N-5007 Bergen, Norway

*Corresponding author: dfogg@uottawa.ca, Deryn.Fogg@uib.no, Vidar.Jensen@uib.no

## Table of Contents

**S1. EXPERIMENTAL PROCEDURES** ..... **2**

S1.1. SYNTHESIS OF RU COMPLEXES ..... 2

*S1.1.1. Synthesis of Carbide Complexes $RuX_2(CAAC)(P^iPr_3)(\equiv C:)$* ..... *3*

*S1.1.2. Synthesis of Piers Complexes $[RuX_2(CAAC)(=CHP(^iPr)_3)]OTf$* ..... *4*

*S1.1.3. Synthesis of Pyridine Adducts $RuX_2(L)(py)_n(=CH_2)$* ***1*** ..... *6*

*S1.1.4. Bimolecular Coupling of Methylidene Complex* ***1*** ..... *6*

**S2. NMR SPECTRA** ..... **9**

S2.1. CARBIDE COMPLEXES ..... 9

S2.2. PIERS CATALYSTS ..... 12

S2.3. PYRIDINE-STABILIZED METHYLIDENE COMPLEXES ..... 15

**S3. COMPUTATIONAL RESULTS AND DISCUSSION** ..... **18**

S3.1. MECHANISM OF BIMOLECULAR COUPLING OF RU METHYLIDENE ..... 18

S3.2. $H_2$IMES VS **C3**$^{PH}$ ..... 23

S3.3. COMPUTATIONAL MODELS FOR CATALYSTS BEARING CARBENES **C1**$^{PH}$, **C2**$^{ME}$, AND **C1**$^{ME}$ ..... 29

**S4. COMPUTATIONAL METHODS** ..... **33**

S4.1. LOCATION OF STATIONARY POINTS AND MINIMUM ENERGY CROSSING POINTS (MECPS) ..... 33

S4.2. HESSIAN CALCULATIONS AND THERMOCHEMICAL CORRECTIONS ..... 34

S4.3. SINGLE-POINT ENERGY CALCULATIONS ..... 34

S4.4. CALCULATION OF GIBBS FREE ENERGIES ..... 35

*S4.4.1. The Barrier of Diffusion-Controlled Reactions* ..... *35*

*S4.4.2. The Barrier Associated with Spin Inversion* ..... *35*

S4.5. NATURAL BOND ORDER AND PARTIAL CHARGE ANALYSIS ..... 36

S4.6. CALCULATIONS OF BURIED VOLUME ..... 36

**S5. COMPUTATIONAL DATA** ..... **37**

**S6. REFERENCES** ..... **41**

## S1. Experimental Procedures

**General Procedures.** Reactions were carried out under $N_2$ using glovebox or Schlenk techniques. HPLC-grade $CH_2Cl_2$, *n*-hexane, $C_6H_6$ were dried and degassed using a Glass Contour solvent purification system, toluene by distillation from Na: all were stored under $N_2$ over 4 Å molecular sieves for at least 18 h prior to use. NMR solvents ($CDCl_3$, $C_6D_6$ and $C_7D_8$: Cambridge Isotopes) were degassed (freeze/pump/thaw; 5x), and stored as above. CAAC salts[1] and Feist's ester[2] were prepared by literature methods. Known Hoveyda-class CAAC complexes,[3,4] iodide derivative **HC1$^{Ph}$(I$_2$)**,[5] carbide complexes [$RuCl_2(L)PR_3(\equiv C:)$] and Piers catalysts [$RuCl_2(L)(=CHPR_3)$]OTf (L = $H_2$IMes, R = Cy;[2,6] L = **C1$^{Me}$**,[5] or **C1$^{Ph}$**,[7] R = $^i$Pr) were synthesized by literature methods. Ethylene (BOC Gases; Linde, 99.9%), internal standard dimethyl terephthalate (DMT, Aldrich, 99%) and P$^i$Pr$_3$ (Alfa, 98%) were used as received. The latter was stored in the glovebox freezer until use.

NMR spectra were recorded on Advance II 500 spectrometers at 23±0.2 °C, unless otherwise specified. Chemical shifts are reported in ppm, referenced to the residual proton or carbon signals of the solvent. MALDI mass spectra were collected on a Bruker UltrafleXtreme MALDI-TOF/TOF mass spectrometer. Solutions of matrix (pyrene) and analyte (20:1) were prepared such that the final concentration of matrix and analyte was ca. 100 mM and 5 mM, respectively. A 1 µL aliquot of the mixed solution was spotted on the MALDI target plate and allowed to evaporate by the dried-droplet method.[8] Spectra were calibrated internally with pyrene (*m/z* 202.078).

### S1.1. Synthesis of Ru Complexes

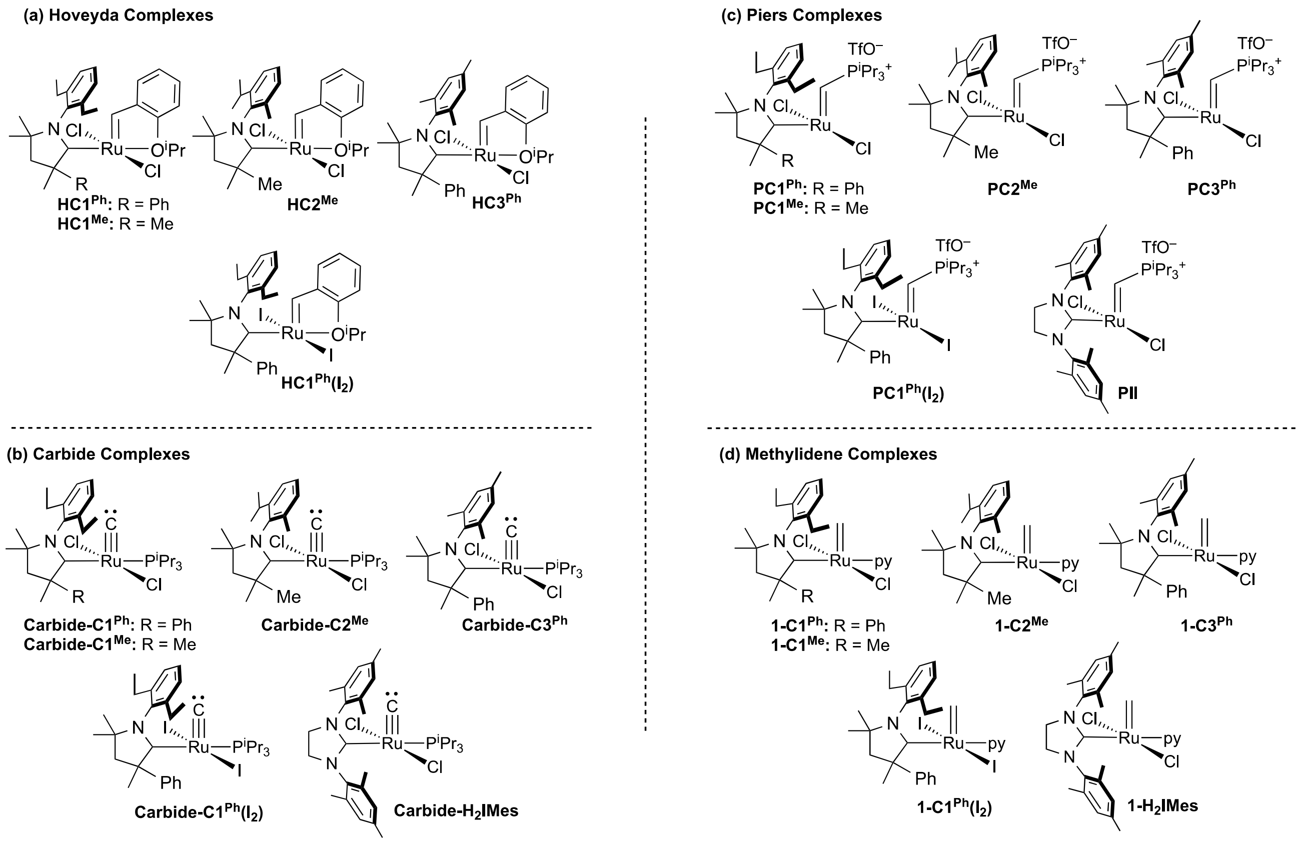


**Chart S1.** Overview of complexes synthesized, showing numbering system.

### S1.1.1. Synthesis of Carbide Complexes $RuX_2$(CAAC)($P^iPr_3$)(≡C:)

Higher yields of the CAAC carbide complexes were obtained, relative to our prior route,[7] by starting with the known Hoveyda-CAAC complexes rather than the indenylidene CAAC dimer (Scheme S1). Washing the product with *n*-hexane sufficed to remove the organic side-products, where our original procedure required chromatography to remove a species that gave rise to a broad aromatic signal.

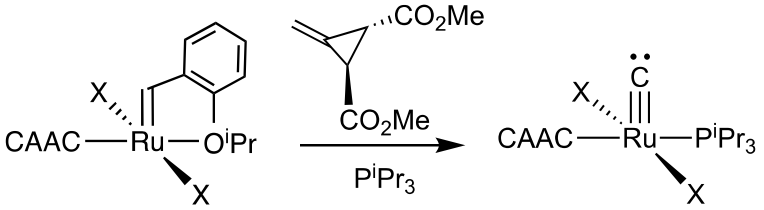


**Scheme S1.** Synthesis of Carbide Complexes.

**Representative Procedure for Synthesis of $RuCl_2(C1^{Ph})(P^iPr_3)$(≡C:), Carbide-$C1^{Ph}$.** To a green solution of **$HC1^{Ph}$** (130 mg, 0.203 mmol) and Feist's ester (55 mg, 0.32 mmol, 1.6 equiv) in 3 mL $CH_2Cl_2$ was added a solution of $P^iPr_3$ (53 mg, 0.33 mmol, 1.6 equiv) in 1 mL $CH_2Cl_2$. The solution was transferred to an oil bath at 35 °C and stirred for 20 h to give a dark yellow solution in which complete conversion to **Carbide-$C1^{Ph}$** was evident ($^1H$ NMR). The volatiles were removed under vacuum, and the residue was taken up in *n*-hexane (2 mL). The suspension was stirred vigorously until a fine powder resulted. The suspension was chilled (–35 °C) for 2 h, after which the pale-yellow solid was filtered off, washed with *n*-hexane (–35 °C, 1 mL), dried, and sublimed at 60 °C under reduced pressure for 2 h. Yield: 117 mg, 82%. The $^1H$ NMR data in $CDCl_3$ agree with values reported.[7] Particularly diagnostic is the most downfield signal: δ 7.73 (d, $^3J_{HH}$ = 6.4 Hz, 2H, *m*-C*H* of **$C1^{Ph}$** Ar).

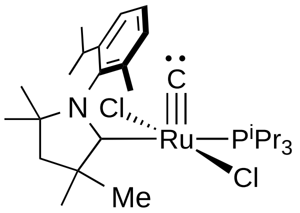


**Synthesis of Carbide-$C2^{Me}$.** As in the representative procedure, with **$HC2^{Me}$** (112 mg, 0.194 mmol), Feist's ester (45 mg, 0.27 mmol, 1.4 equiv) and $P^iPr_3$ (44 mg, 0.28 mmol, 1.4 equiv). $^1H$ NMR analysis indicated complete consumption of starting material after 6 h of reaction. Product obtained as a pale-yellow solid. Yield after workup: 84 mg (72%). For NMR spectra (including the assigned $^1H$ spectrum), see Figure S2 and Figure S3.
$^1H$ NMR ($CDCl_3$, 500 MHz): δ 7.29 (d, $^3J_{HH}$ = 4.5 Hz, 2H, *m*-C*H* of **$C2^{Me}$** Ar), 7.14 (t, $^3J_{HH}$ = 4.6 Hz, 1H, *p*-C*H* of **$C2^{Me}$** Ar), 3.05 (sept, $^3J_{HH}$ = 6.5 Hz, 1H, C*H* of **$C2^{Me}$** $^i$Pr), 2.74 (m, 3H, C*H* of $P^iPr_3$), 2.42 (s, 3H, $CH_3$ of **$C2^{Me}$**), 2.09 (d, $^2J_{HH}$ = 3.3 Hz, 2H, backbone $CH_2$ of **$C2^{Me}$**), 1.75 (d, $^3J_{HH}$ = 16.9 Hz, 6H $CH_3$ of **$C2^{Me}$** $^i$Pr), 1.47 (s, 3H, $CH_3$ of **$C2^{Me}$** or $CH_3$ of $P^iPr_3$), 1.41 (s, 3H, $CH_3$ of **$C2^{Me}$** or $CH_3$ of $P^iPr_3$), 1.34 (m, 21H, $CH_3$ of $P^iPr_3$ and **$C2^{Me}$**, overlapping), 1.17 (d, *J* = 6.4 Hz, 3H, $CH_3$ of **$C2^{Me}$** or $CH_3$ of $P^iPr_3$). $^{13}C\{^1H\}$ NMR ($CDCl_3$, 125 MHz): δ 474.0, 266.5, 265.9, 147.5, 138.5, 137.1, 129.0, 128.9, 125.1, 80.1, 58.4, 52.3, 31.0, 30.8, 30.5, 28.7, 28.4, 26.5, 24.4, 22.9, 21.7, 19.7, 19.6. MALDI-TOF MS (pyrene matrix), *m/z*: [**Carbide-$C2^{Me}$**]$^+$ 601.127 (calculated for $C_{28}H_{48}Cl_2NPRu$: 601.194).

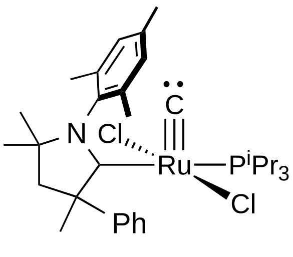


**Synthesis of Carbide-$C3^{Ph}$.** As in the representative procedure, with **$HC3^{Ph}$** (148 mg, 0.237 mmol), Feist's ester (52 mg, 0.31 mmol, 1.3 equiv) and $P^iPr_3$ (51 mg, 0.32 mmol, 1.4 equiv). $^1H$ NMR analysis indicated complete consumption of starting material after 2 h. Product obtained as a beige solid.

Yield after workup: 130 mg (84%). For NMR spectra (including the assigned $^{1}$H spectrum), see Figure S4 and Figure S5.
$^{1}$H NMR ($CDCl_3$, 500 MHz): Note. Signals are broad, causing small deviations in integration. δ 7.82 (s br, 2H, *m* or *p*-C*H* of **C3$^{Ph}$** Ph), 7.66 (s br, 1H, *m*-C*H* of **C3$^{Ph}$** Ar or *p*-C*H* of **C3$^{Ph}$** Ph), 7.42 – 7.17 (m br, 4H, *m* or *p*-C*H* of **C3$^{Ph}$** Ph and C*H*$Cl_3$, overlapping), 6.93 (s br, 1H, *m*-C*H* of **C3$^{Ph}$** Ar or *p*-C*H* of **C3$^{Ph}$** Ph), 6.86 (s br, 1H, *m*-C*H* of **C3$^{Ph}$** Ar or *p*-C*H* of **C3$^{Ph}$** Ph), 2.66 (s br, 3H, C*H* of $P^iPr_3$), 2.47 (m br, 6H, $CH_3$ of **C3$^{Ph}$**), 2.39 (s br, 4H, $CH_3$ and backbone $CH_2$ of **C3$^{Ph}$**), 2.26 (s br, 4H, $CH_3$ of **C3$^{Ph}$**), 1.58 (s br, 1H, backbone $CH_2$ of **C3$^{Ph}$**), 1.46 (s br, 3H, $CH_3$ of **C3$^{Ph}$**), 1.28 (s br, 2H, $CH_3$ of **C3$^{Ph}$**), 1.13 (m br, 20H, $CH_3$ of $P^iPr_3$). $^{13}$C{$^{1}$H} NMR ($CDCl_3$, 125 MHz): δ 435.0, 265.1, 264.4, 148.4, 138.2, 136.0, 135.7, 130.3, 130.3, 130.0, 129.4, 128.3, 126.89, 126.9, 126.5, 81.7, 80.4, 66.4, 54.9, 29.8, 29.5, 28.9, 25.0, 24.2, 21.8, 21.6, 20.8, 19.3. MALDI-TOF MS (pyrene matrix), *m/z*: [**Carbide-C3$^{Ph}$**–Cl,$^{i}$Pr]$^{+}$ 517.109 (calc'd for $C_{29}H_{41}ClNPRu$: 517.171).

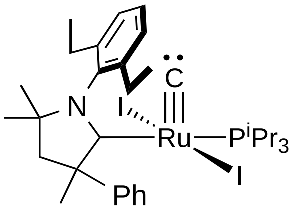

**Synthesis of Carbide-C1$^{Ph}$(I$_2$).** As in the representative procedure, with minor modifications: **HC1$^{Ph}$(I$_2$)** (193 mg, 0.235 mmol), Feist's ester (61 mg, 0.36 mmol, 1.5 equiv), $P^iPr_3$ (57 mg, 0.36 mmol, 1.5 equiv). Toluene was used as solvent. No starting material remained after 22 h at 80 °C. Yield of brown product after workup: 176 mg (88%). For NMR spectra (including the assigned $^{1}$H NMR spectrum), see Figure S6 and Figure S7.
$^{1}$H NMR ($C_6D_6$, 500 MHz): δ 7.79 (d, $^{3}J_{HH}$ = 7.5 Hz, 2H, C*H* of **C1$^{Ph}$** Ar or Ph), 7.34 (t, $^{3}J_{HH}$ = 7.9 Hz, 2H, C*H* of **C1$^{Ph}$** Ar or Ph), 7.18 (s, 2H, C*H* of **C1$^{Ph}$** Ar or Ph), 7.13 (d, $^{3}J_{HH}$ = 8.1 Hz, 1H, C*H* of **C1$^{Ph}$** Ar or Ph), 7.05 (t, $^{3}J_{HH}$ = 7.6 Hz, 1H, *p*-C*H* of **C1$^{Ph}$** Ar or Ph), 3.64 (dq, $^{3}J_{HH}$ = 15.0, 7.4 Hz, 1H, $CH_2$ of **C1$^{Ph}$** Ar), 3.45 (dq, $^{3}J_{HH}$ = 14.6, 7.3 Hz, 1H, $CH_2$ of **C1$^{Ph}$** Ar), 3.08 (m, 3H, C*H* of $P^iPr_3$), 2.88 (d, $^{2}J_{HH}$ = 12.3 Hz, 1H, backbone $CH_2$ of **C1$^{Ph}$**), 2.73 (dq, $^{3}J_{HH}$ = 14.7, 7.3 Hz, 2H, $CH_2$ of **C1$^{Ph}$** Ar), 2.42 (s, 3H, backbone $CH_3$ of **C1$^{Ph}$**), 1.69 (d, $^{2}J_{HH}$ = 12.3 Hz, 1H, backbone $CH_2$ of **C1$^{Ph}$**), 1.39 (t, $^{3}J_{HH}$ = 7.4 Hz, 3H, $CH_3$ of **C1$^{Ph}$** Ar), 1.34 (t, $^{3}J_{HH}$ = 7.4 Hz, 3H, $CH_3$ of **C1$^{Ph}$** Ar), 1.28 (dd, $^{3}J_{HH}$ = 13.4, 7.3 Hz, 6H, $CH_3$ of $P^iPr_3$ and backbone $CH_3$ of **C1$^{Ph}$**, overlapping), 1.18 – 1.11 (m, 15H, $CH_3$ of $P^iPr_3$). $^{13}$C{$^{1}$H} NMR ($C_6D_6$, 125 MHz): δ 269.6, 269.0, 147.7, 144.1, 142.1, 139.5, 129.4, 129.2, 127.0, 126.8, 126.6, 79.0, 67.4, 67.4, 36.2, 28.0, 27.0, 25.8, 25.5, 25.4, 20.4, 20.3, 15.8, 15.0. MALDI-TOF MS (pyrene matrix), *m/z*: [**Carbide-C1$^{Ph}$–I$_2$**]$^{+}$ 593.211 (calc'd for $C_{33}H_{50}NPRu$: 593.272).

### S1.1.2. Synthesis of Piers Complexes [RuX$_2$(CAAC)(=CHP($^{i}$Pr)$_3$)]OTf

**Representative Procedure for Synthesis of [RuCl$_2$(C1$^{Ph}$)(=CHP$^{i}$Pr$_3$)]OTf, PC1$^{Ph}$.** The Piers complexes were prepared according to the method reported[7] for **C1$^{Ph}$** (Scheme S2; reproduced here for convenience). To a stirred yellow solution of **Carbide-C1$^{Ph}$** (117 mg, 0.166 mmol) in 4 mL $CH_2Cl_2$ was added a solution of HOTf (27 mg, 0.18 mmol, 1.1 equiv) in 1.5 mL $CH_2Cl_2$. The solution immediately turned dark-green. The mixture was stirred for 45 min at RT, after which the solvent was removed under vacuum. A fine green precipitate was obtained by adding *n*-hexane (2 mL) and stirring vigorously. This material was filtered off, washed with *n*-hexane (2 mL) and dried. Yield: 127 mg (89%). The $^{1}$H NMR data in $CDCl_3$ agree with values reported.[7] Key alkylidene signal: δ 18.67 (d, $^{2}J_{HP}$ = 35.3 Hz, 1H, Ru=C*H*).

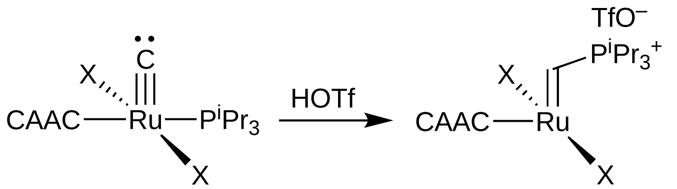


**Scheme S2.** Synthesis of Piers Complexes.

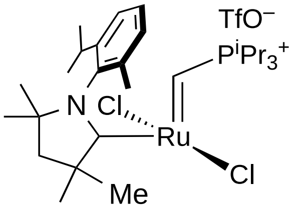


**Synthesis of PC2$^{\mathbf{Me}}$.** As above, with **Carbide-C2$^{\mathbf{Me}}$** (70 mg, 0.116 mmol) and HOTf (18 mg, 0.12 mmol, 1 equiv). Product obtained as a light-green solid. Yield after workup: 75 mg (80%). For NMR spectra (including the assigned $^{1}$H spectrum), see Figure S8 and Figure S9.
$^{1}$H NMR ($CDCl_3$, 500 MHz): δ 18.12 (d, $^{2}J_{HP}$ = 41.9 Hz, 1H, Ru=C*H*), 7.60 – 7.49 (m, 2H, *m*-C*H* of **C2$^{\mathbf{Me}}$** Ar), 7.34 (d, $^{3}J_{HH}$ = 7.3 Hz, 1H, *p*-C*H* of **C2$^{\mathbf{Me}}$** Ar), 3.20 – 3.11 (m, 3H, C*H* of P$^{i}$Pr$_3$), 3.10–3.05 (m, 1H, C*H* of **C2$^{\mathbf{Me}}$** $^{i}$Pr), 2.30 (d, $^{2}J_{HH}$ = 1.6 Hz, 2H, backbone C$H_2$ of **C2$^{\mathbf{Me}}$**), 2.22 (s, 3H, C$H_3$ of **C2$^{\mathbf{Me}}$**), 2.18 (s, 3H, C$H_3$ of **C2$^{\mathbf{Me}}$**), 2.13 (s, 3H, C$H_3$ of **C2$^{\mathbf{Me}}$**), 1.46 (s, 3H, C$H_3$ of **C2$^{\mathbf{Me}}$**), 1.39 – 1.34 (m, 6H, C$H_3$ of **C2$^{\mathbf{Me}}$** and C$H_3$ of P$^{i}$Pr$_3$, overlapping), 1.30 (m, 5H, C$H_3$ of P$^{i}$Pr$_3$), 1.22 (m, 6H, C$H_3$ of P$^{i}$Pr$_3$), 0.87 (d, $^{3}J_{HH}$ = 6.6 Hz, 3H, C$H_3$ of P$^{i}$Pr$_3$). $^{13}$C{$^{1}$H} NMR ($CDCl_3$, 125 MHz): δ 254.6, 232.6, 148.4, 136.9, 130.3, 130.2, 126.6, 81.1, 51.9, 29.6, 29.3, 29.0, 28.7, 26.6, 24.3, 24.0, 23.9, 18.3, 18.2, 14.3. MALDI-TOF MS (pyrene matrix), *m/z*: [**PC2$^{\mathbf{Me}}$–Cl,H**]$^{+}$ 715.171 (calc'd for $C_{29}H_{48}ClF_3NO_3PRuS$: 715.178).

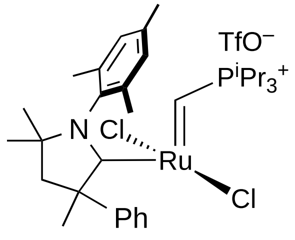


**Synthesis of PC3$^{\mathbf{Ph}}$.** As above, with **Carbide-C3$^{\mathbf{Ph}}$** (70 mg, 0.116 mmol) and HOTf (18 mg, 0.12 mmol, 1 equiv). Product obtained as a green solid. Yield after workup: 113 mg (82%). For NMR spectra (including the assigned $^{1}$H spectrum), see Figure S10 and Figure S11.
$^{1}$H NMR ($CDCl_3$, 500 MHz): δ 18.74 (d, $^{2}J_{HP}$ = 34.0 Hz, 1H, Ru=C*H*), 7.79 (s, 1H, *m*-C*H* of **C3$^{\mathbf{Ph}}$** Mes), 7.53 (t, $^{3}J_{HH}$ = 7.7 Hz, 2H, *m*-C*H* of **C3$^{\mathbf{Ph}}$** Ph), 7.38 (t, $^{3}J_{HH}$ = 7.5 Hz, 1H, *p*-C*H* of **C3$^{\mathbf{Ph}}$** Ph), 7.26 (s, 1H, *m*-C*H* of **C3$^{\mathbf{Ph}}$** Mes), 7.12 (d, $^{3}J_{HH}$ = 10.0 Hz, 2H, *o*-C*H* of **C3$^{\mathbf{Ph}}$** Ph), 2.94 (m, 3H, C*H* of P$^{i}$Pr$_3$), 2.77 (s, 1H, backbone C$H_2$ of **C3$^{\mathbf{Ph}}$**), 2.49 (d, $^{2}J_{HH}$ = 13.3 Hz, 1H, backbone C$H_2$ of **C3$^{\mathbf{Ph}}$**), 2.38 (m, 9H, C$H_3$ of **C3$^{\mathbf{Ph}}$**), 2.03 (s, 3H, C$H_3$ of **C3$^{\mathbf{Ph}}$**), 1.50 (s, 3H, C$H_3$ of **C3$^{\mathbf{Ph}}$**), 1.39 (s, 3H, C$H_3$ of **C3$^{\mathbf{Ph}}$**), 1.11 (m, 18H, C$H_3$ of P$^{i}$Pr$_3$). $^{13}$C{$^{1}$H} NMR ($CDCl_3$, 125 MHz): δ 241.9, 140.5, 137.9, 136.5, 134.7, 131.4, 131.2, 130.6, 128.6, 126.3, 81.2, 64.0, 31.7, 30.5, 29.6, 28.1, 22.8, 22.1, 21.8, 21.1, 17.9, 17.8, 17.7, 14.3. MALDI-TOF MS (pyrene matrix), *m/z*: [**PC3$^{\mathbf{Ph}}$–Cl, OTf**]$^{+}$ 615.201 (calc'd for $C_{32}H_{49}ClNPRu$: 615.233).

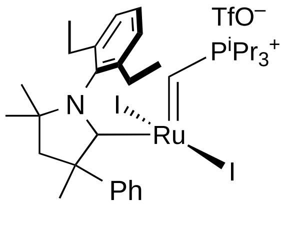


**Synthesis of PC1$^{\mathbf{Ph}}$(I$_2$).** As above, with minor modifications: **Carbide-C1$^{\mathbf{Ph}}$(I$_2$)** (174 mg, 0.205 mmol) and HOTf (34 mg, 0.23 mmol, 1.1 equiv). $C_6H_6$ was chosen as the solvent to avoid possible halide exchange during reaction, which was stirred for 2.5 h at RT. Product obtained as an olive-green solid. Yield after workup: 179 mg (87%). To limit the possibility of halide exchange, NMR analysis was carried out in $CD_2Cl_2$. For NMR spectra (including the assigned $^{1}$H spectrum), see Figure S12 and Figure S13.
$^{1}$H NMR ($CD_2Cl_2$, 500 MHz): δ 16.66 (d, $^{2}J_{HP}$ = 30.1 Hz, 1H, Ru=C*H*), 8.71 (s br, 2H, C*H* of **C1$^{\mathbf{Ph}}$** Ar or Ph), 8.10 (m br, 3H, C*H* of **C1$^{\mathbf{Ph}}$** Ar or Ph), 8.03 – 7.88 (m br, 3H, C*H* of **C1$^{\mathbf{Ph}}$** Ar or Ph), 3.70 (s br, 1H, backbone C*H* of **C1$^{\mathbf{Ph}}$**), 3.44 (m, 3H, C*H* of P$^{i}$Pr$_3$), 3.31 – 3.23 (s br, 1H, backbone C*H* of **C1$^{\mathbf{Ph}}$**), 3.17 (m br, 1H, C$H_2$ of **C1$^{\mathbf{Ph}}$** Ar), 3.07 (m br, 1H, C$H_2$ of **C1$^{\mathbf{Ph}}$** Ar), 2.95 (m, 2H, C$H_2$ of **C1$^{\mathbf{Ph}}$** Ar), 2.09

(d br, $^3J_{HH}$ = 5.7 Hz 3H, C$H_3$ of **C1$^{Ph}$** Ar), 1.92 (m, 7H, C$H_3$ of **C1$^{Ph}$** Ar and backbone, overlapping), 1.75 (m, 6H, C$H_3$ of **C1$^{Ph}$** backbone), 1.63 (m, 19H, C$H_3$ of P$^i$Pr$_3$). $^{13}$C{$^1$H} NMR ($CD_2Cl_2$, 125 MHz): δ 248.9, 146.6, 142.8, 142.1, 137.6, 132.7, 131.5, 130.6, 128.4, 124.3, 81.1, 63.4, 33.3, 32.1, 31.4, 28.4, 28.0, 25.5, 21.9, 21.6, 19.9, 19.6, 19.0, 18.8, 18.2, 17.2, 17.0, 14.3, 14.1. MALDI-TOF MS (pyrene matrix), *m/z*: [**PC1$^{Ph}$(I$_2$)**]$^+$ 721.130 (calc'd for $C_{33}H_{51}INPRu$: 721.184).

### S1.1.3. Synthesis of Pyridine Adducts RuX$_2$(L)(py)$_n$(=CH$_2$) 1

The pyridine adducts were prepared by minor modification of the method reported for **1-C1$^{Ph}$** and **1-H$_2$IMes** (Scheme S3).[7,9] A representative procedure is provided for **1-C1$^{Ph}$**.

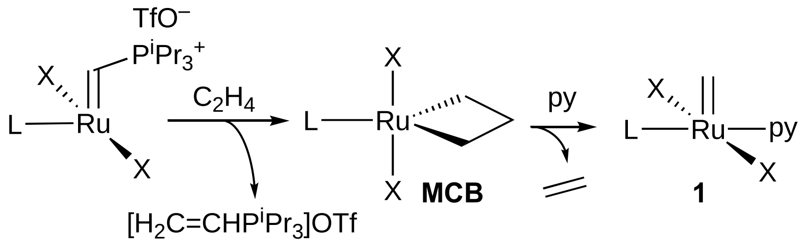


**Scheme S3.** Synthesis of Pyridine Adducts.

**Representative Procedure for Synthesis of RuCl$_2$(C1$^{Ph}$)(py)(=CH$_2$), 1-C1$^{Ph}$.** In a 10 mL Schlenk flask, a dark-green solution of the **PC1$^{Ph}$** (100 mg, 0.117 mmol) in 2 mL $CH_2Cl_2$ was frozen in $N_{2(l)}$. The sample was freeze-pump-thaw degassed 3x, and thawed under 1 atm ethylene at –45 °C (MeCN–dry ice bath). The resulting dark red solution was stirred for 15 min, briefly exposed to vacuum to remove ethylene, then stirred under $N_2$. A solution of pyridine (18.7 µL, 0.231 mmol, 2 equiv) in 500 µL *n*-hexane, chilled in an EtOH-$N_{2(l)}$ cold bath (–110 to –80 °C), was then added via syringe as a slow dribble down the flask wall (ca. 10 sec). The solution rapidly turned dark green, then brown-yellow. The mixture was stirred for a further 10 min. Cold *n*-hexane (4 mL; –110 to –80 °C) was then added using a cannula chilled with dry ice. The mixture was stirred for 10 min to precipitate the brown phosphonium salt [H$_2$C=CHP$^i$Pr$_3$]OTf, filtered cold, and the orange filtrate was immediately stripped to dryness at –45 °C to afford yellow **1-C1$^{Ph}$**. The flask containing solid **1-C1$^{Ph}$** was transferred to a –35 °C freezer inside a glovebox until use. Product masses were not measured, given their high static charge, and the high relative error inherent in weighing small amounts of material in even a small Schlenk flask on a balance in the gloveobox. Instead, Ru concentrations for the decomposition experiments were quantified based on known concentrations of injected internal standard: see next section.

NMR data for **1-C1$^{Ph}$** and **1-H$_2$IMes** agree with values reported.[7] For NMR spectra, see Figure S14, Figure S15, Figure S16, Figure S17, and Figure S18. Key [Ru]=C$H_2$ signals: $^1$H NMR ($CDCl_3$, 500 MHz, –40 °C): **1-C1$^{Ph}$**, δ 18.35 ppm; **1-H$_2$IMes**, δ 18.76 ppm; **1-C1$^{Me}$**, δ 17.98 ppm; **1-C3$^{Ph}$**, δ 18.31 ppm; **1-C1$^{Ph}$(I$_2$)**, δ 16.73 ppm; **1-C2$^{Me}$**, 18.22 ppm (not isolated, generated in situ).

### S1.1.4. Bimolecular Coupling of Methylidene Complex 1

The procedure for the coupling reaction is summarized in Scheme S4. In our original study, the objective was quantitation of ethylene evolved from **1-C1$^{Ph}$**, and considerations of purity were minor.[7] The solid methylidene complex **1-C1$^{Ph}$** was thus not purified to remove the phosphonium salt [H$_2$C=CHP$^i$Pr$_3$]OTf. In the present kinetics study, removal of phosphonium salt as described above yielded a powder with a high static charge, which could not be weighed: moreover, the CAAC

complexes were obtained in low yields owing to their much faster decomposition. Samples were therefore made up by adding a known volume of cold $CDCl_3$ to the Schlenk flask to dissolve the complex, and transferring the solution to an NMR tube containing a known volume of a stock solution of DMT. To protect against warming, the IS stock solution, NMR tubes, pipettes, $CDCl_3$ and sand-bath were kept in the glovebox freezer (–35 °C) for at least 12 h prior to use. To improve accuracy in NMR integrations, the recycle delay (d1) was raised to 0.1 sec from 0.01 sec.

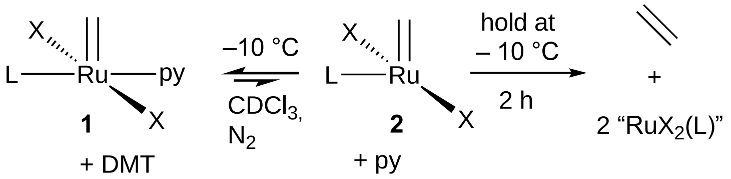


**Scheme S4.** Methylidene Coupling.

**Representative Procedure for Bimolecular Coupling of 1-C1$^{Ph}$:** The Schlenk flask containing solid **1-C1$^{Ph}$** was removed from the glovebox freezer and bedded in a cold sand-bath (chilled to −35 °C by storing in the freezer overnight). To a J-Young NMR tube also bedded in the sand-bath was added cold DMT (20.0 μL of a stock solution of 10 mg DMT in 1 mL $CDCl_3$; 1.03 μmol); NMR integration standard (IS). Solid **1-C1$^{Ph}$** was dissolved in cold $CDCl_3$ (0.6 mL) and transferred to the NMR tube via a chilled pipette. The NMR tube was sealed, transported to the NMR facility in a dry ice-MeCN bath (−45 °C), then quickly shaken and inserted into an NMR probe precooled to −40 °C. The initial integration ratio of **1-C1$^{Ph}$** vs IS was measured, after which the NMR probe was warmed to –10 °C to effect liberation of four-coordinate **2-C1$^{Ph}$**. After an initial 15 min period for thermal equilibration, loss of the [Ru]=C$H_2$ NMR signal was measured over time: see Figure 1 in the main text.

The same procedure was carried out for **1-C1$^{Me}$**, **1-C3$^{Ph}$** and **1-C1$^{Ph}$($I_2$)**. For $H_2$IMes complex **1-$H_2$IMes**, a higher volume of 1.2 mL $CDCl_3$ was used to reduce the headspace, to ensure that any propenes formed via β-H elimination could be detected (none were observed).

**Sample calculation: initial Ru concentration of 2-C1$^{Ph}$, based on known concentration of DMT**.

$$Concentration_{DMT} = \frac{0.010\ (g)}{0.001\ (L)} \bullet \frac{1}{194.18} \frac{(mol)}{(g)} = 0.0515\ (M) \quad \text{Equation S1}$$

For a volume of 20 μL injected into the NMR tube:

$$n_{DMT} = 0.0515 \frac{(mol)}{(L)} \bullet \frac{1}{10^6} \frac{(L)}{(\mu L)} \bullet 20\ (\mu L) = 1.03 \bullet 10^{-6}\ (mol) \quad \text{Equation S2}$$

Ratio of Ru=$CH_2$ protons : DMT methyl protons = 2 H : 6 H

Integration of Ru=$CH_2$ signal : DMT methyl protons = 1.21 : 98.79

$$\frac{n_{Ru}}{n_{DMT}} = \frac{Integration_{Ru}}{Integration_{DMT}} \bullet H\ ratio \quad \text{Equation S3}$$

$$n_{Ru} = \frac{1.21}{98.79} \bullet 1.03 \bullet 10^{-6}\ (mol) \bullet 3 = 3.78 \bullet 10^{-8}\ (mol) \quad \text{Equation S4}$$

$$Initial\ Ru\ concentration = \frac{n_{Ru}}{V_{total}} = \frac{3.78 \bullet 10^{-8}\ (mol)}{0.00062\ (L)} = 6.10 \bullet 10^{-5}\ (M) \quad \text{Equation S5}$$

(a)

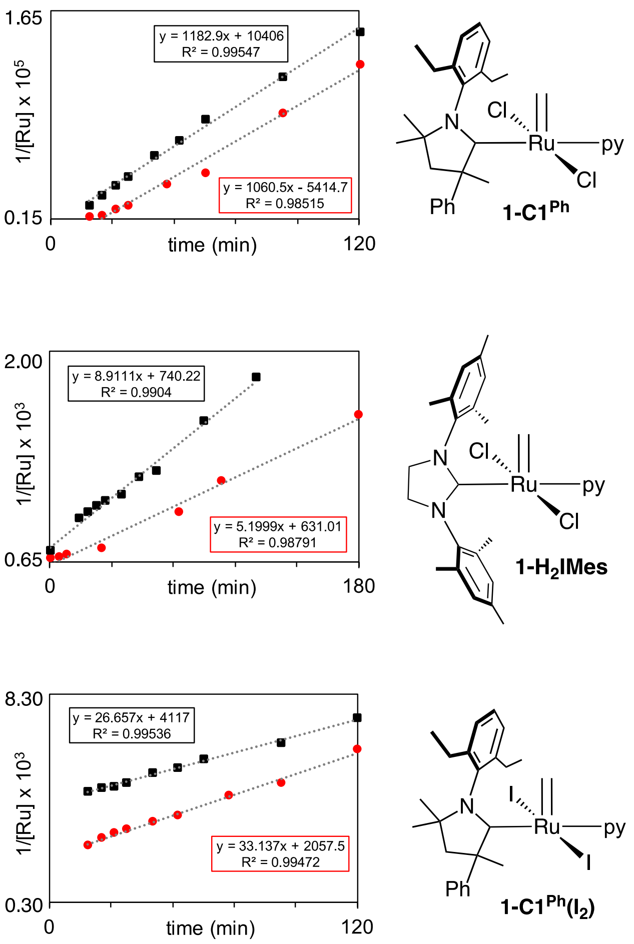


(b)

| **1-L** | 1st trial $k_{obs}$ ($M^{-1}.s^{-1}$) | 2nd trial $k_{obs}$ ($M^{-1}.s^{-1}$) | Average ($M^{-1}.s^{-1}$) | $k_{rel}$ |
|---|---|---|---|---|
| **1-H2IMes** | 0.149 | 0.0867 | 0.118 ± 0.031 | 1 |
| **1-C1$^{Ph}$(I$_2$)** | 0.444 | 0.552 | 0.498 ± 0.054 | 4 |
| **1-C1$^{Ph}$** | 19.7 | 17.7 | 18.7 ± 1.0 | 158 |
| **1-C3$^{Ph}$** | 38.0 | 27.0 | 32.5 ± 5.5 | 275 |
| **1-C1$^{Me}$** | 174 | 105 | 140 ± 34 | 1186 |

**Figure S1.** Second-order rate data for BMC reactions at –10 °C. (a) rate plots with equations and $R^2$ for each run. (b) Tabulated numeric values for $k_{obs}$ obtained in replicate experiments. The $k_{rel}$ value shown is the average between the two trials. The trend in $k_{rel}$ is consistent, but the absolute values of $k_{obs}$ should be treated with caution, as the relative errors range from 5% (**1-C1$^{Ph}$**) to 24% (**1-C1$^{Me}$**).

## S2. NMR Spectra

### S2.1. Carbide Complexes

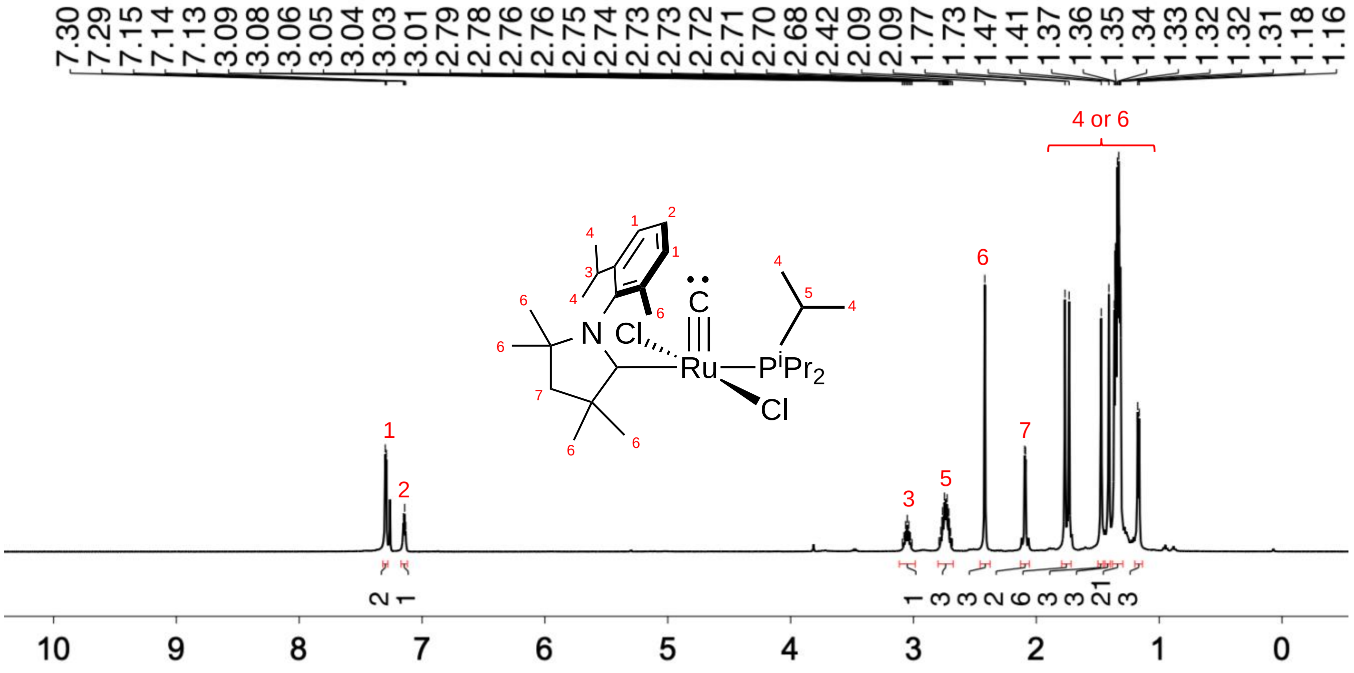

**Figure S2.** $^1$H NMR spectrum ($CDCl_3$, 500 MHz) of **Carbide-C2$^{Me}$**.

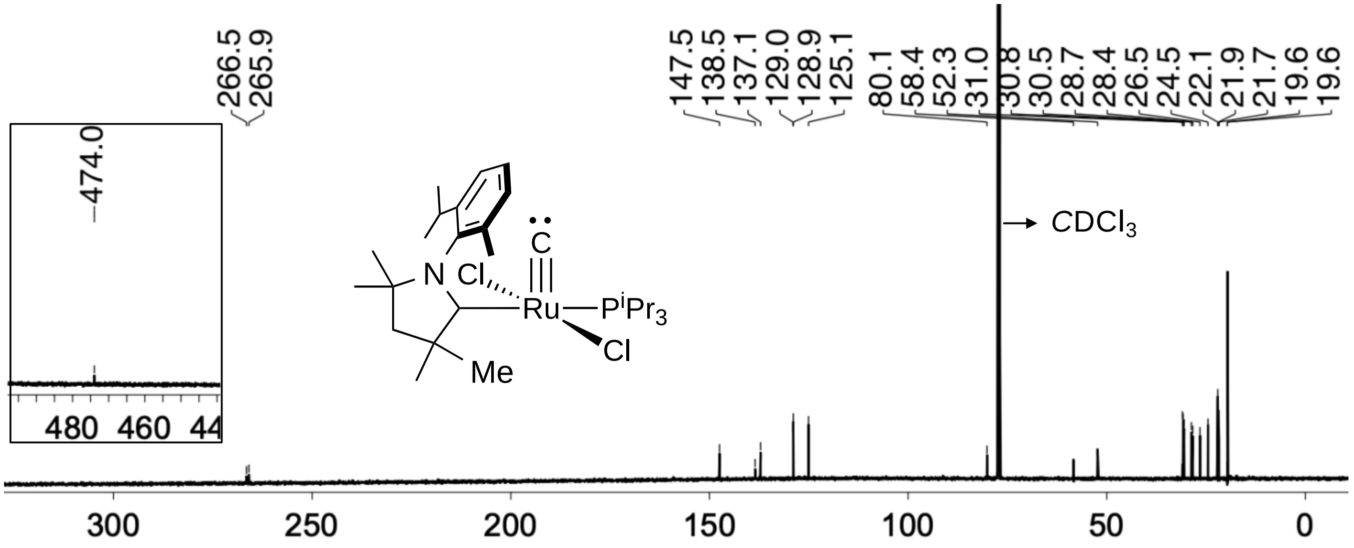

**Figure S3.** $^{13}C\{^1H\}$ NMR spectrum ($CDCl_3$, 125 MHz) of **Carbide-C2$^{Me}$**. Inset shows the carbide carbon.

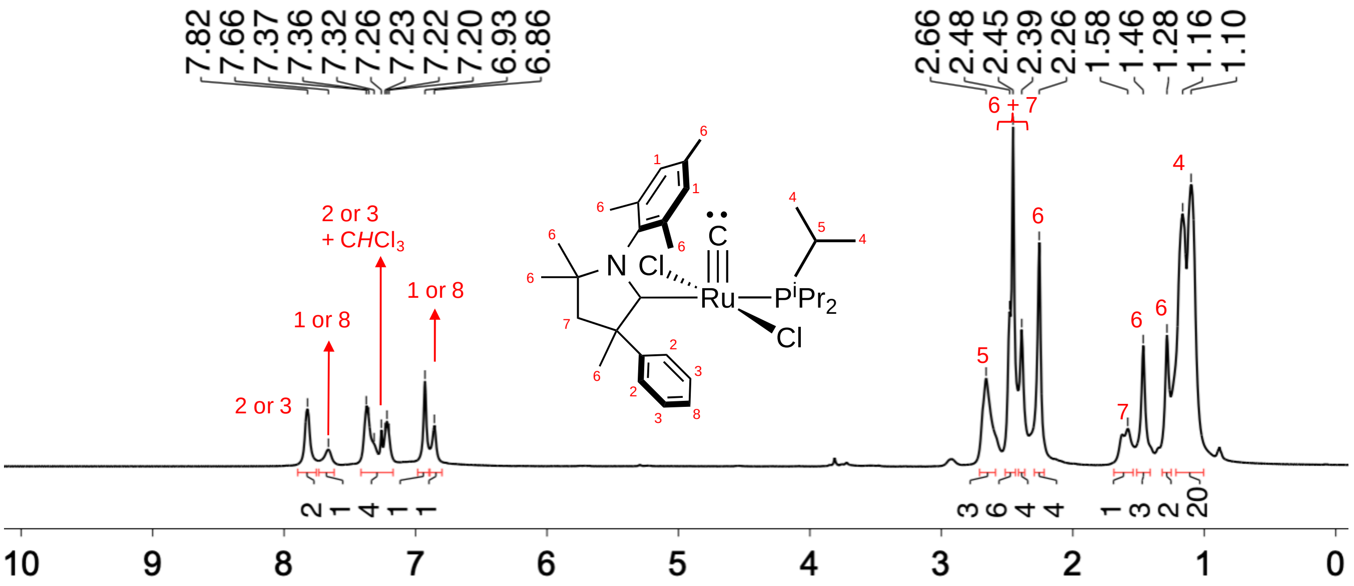

**Figure S4.** $^{1}$H NMR spectrum ($CDCl_3$, 500 MHz) of **Carbide-C3$^{Ph}$**.

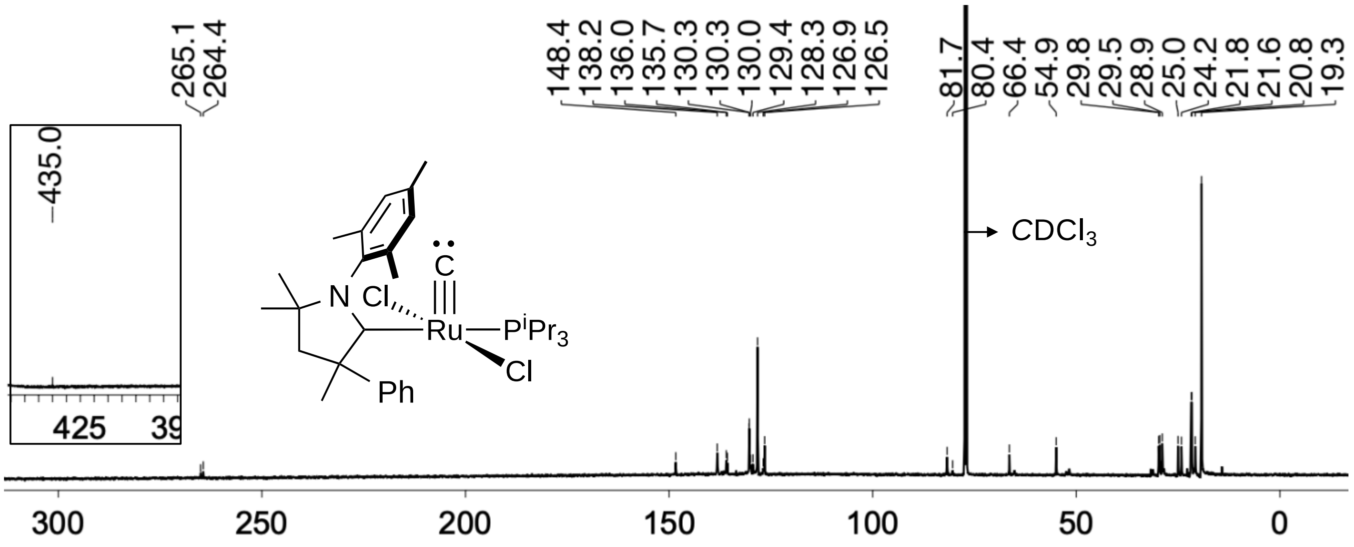

**Figure S5.** $^{13}C\{^{1}H\}$ NMR spectrum ($CDCl_3$, 125 MHz) of **Carbide-C3$^{Ph}$**. Inset shows carbide carbon.

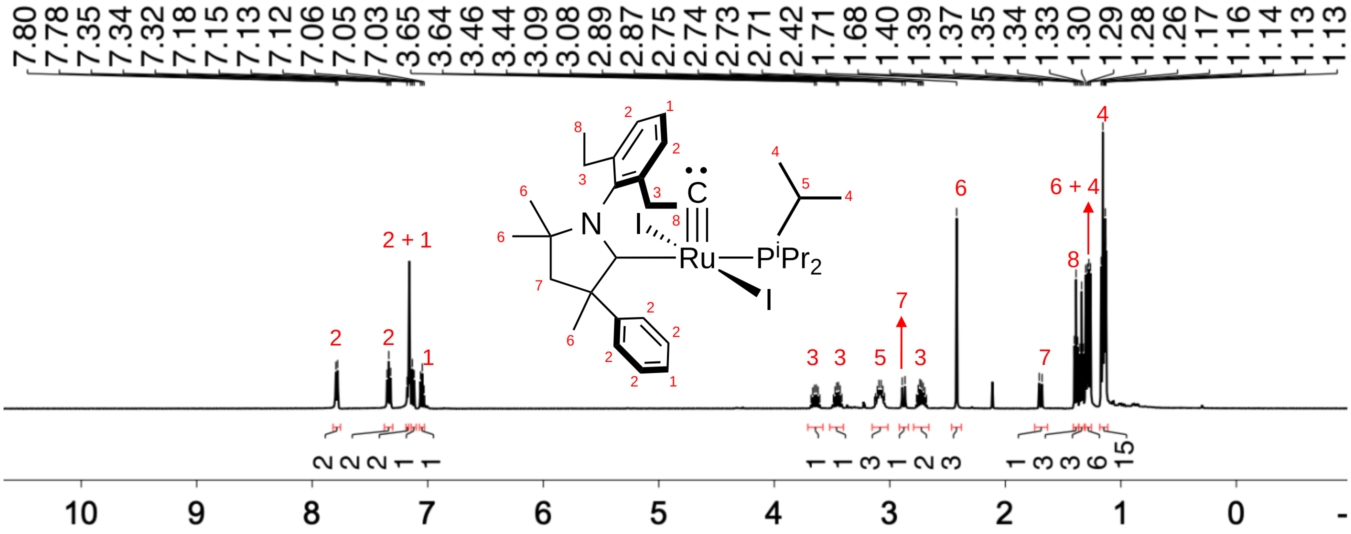


**Figure S6.** $^{1}H$ NMR spectrum ($C_6D_6$, 500 MHz) of **Carbide-C1$^{Ph}$(I$_2$)**.

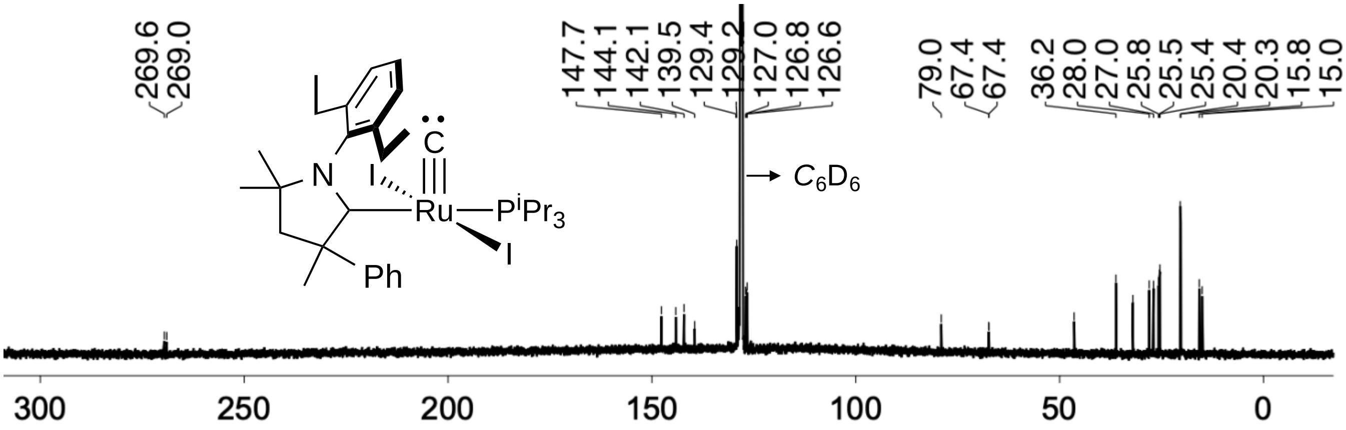


**Figure S7.** $^{13}C\{^{1}H\}$ NMR spectrum ($C_6D_6$, 125 MHz) of **Carbide-C1$^{Ph}$(I$_2$)**. Carbide signal not observed.

## S2.2. Piers Catalysts

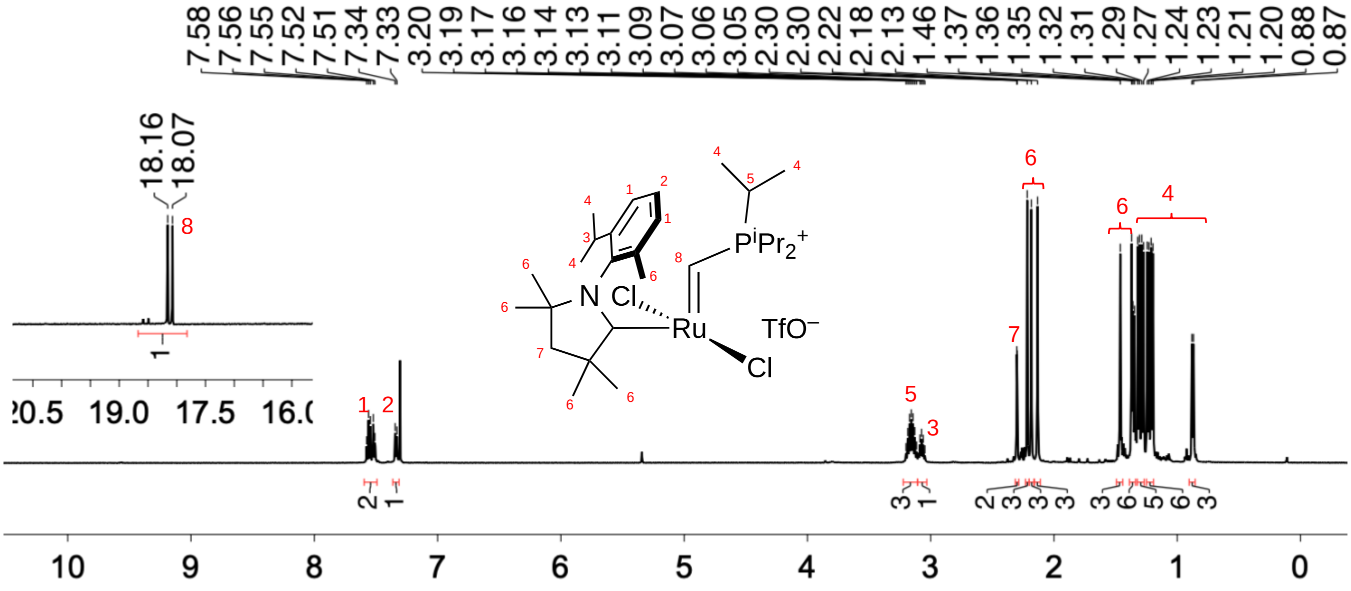

**Figure S8.** $^1$H NMR spectrum ($CDCl_3$, 500 MHz) of **PC2$^{Me}$**. The small downfield signals in the alkylidene region are assigned to a rotamer, as seen for other CAAC complexes.[10]

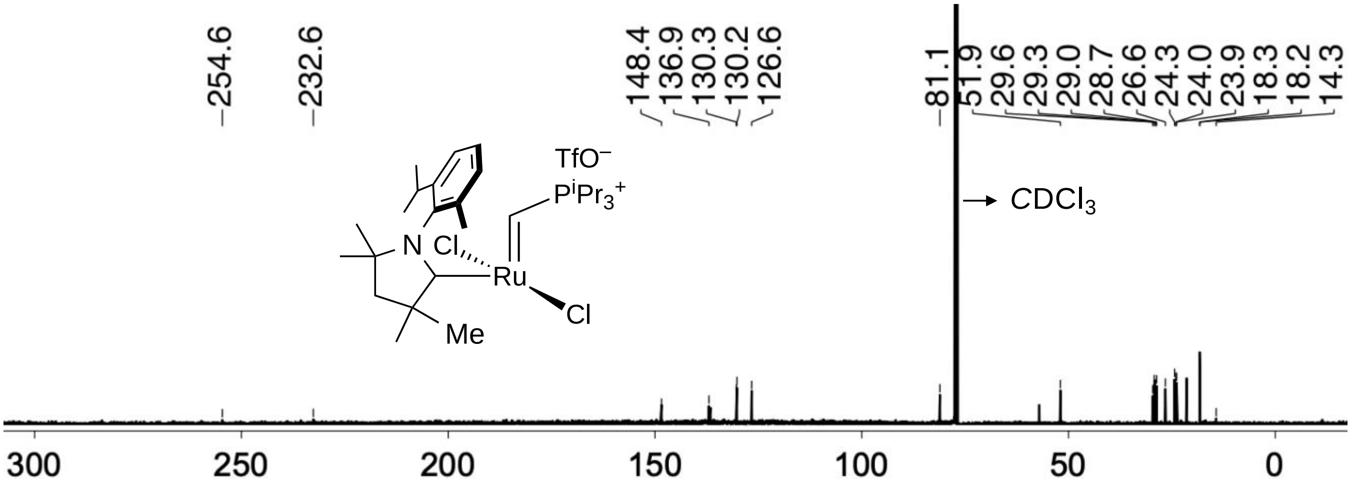

**Figure S9.** $^{13}C\{^1H\}$ NMR spectrum ($CDCl_3$, 125 MHz) of **PC2$^{Me}$**.

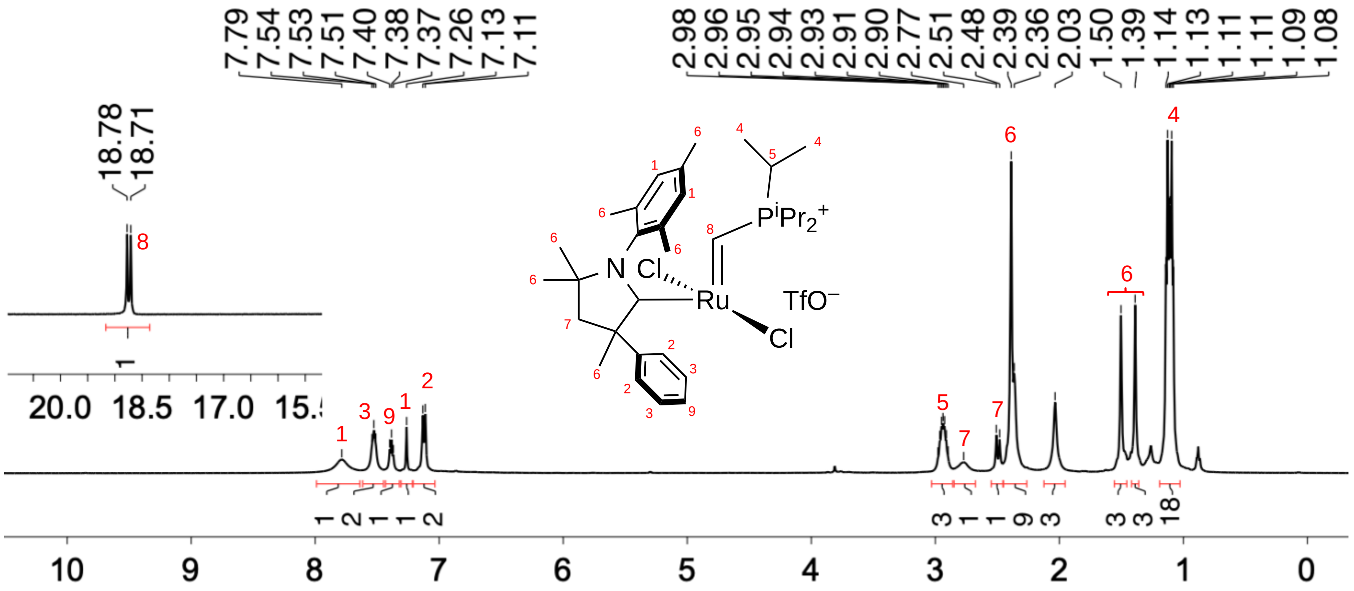

**Figure S10.** $^{1}H$ NMR spectrum ($CDCl_3$, 500 MHz) of **PC3$^{Ph}$**.

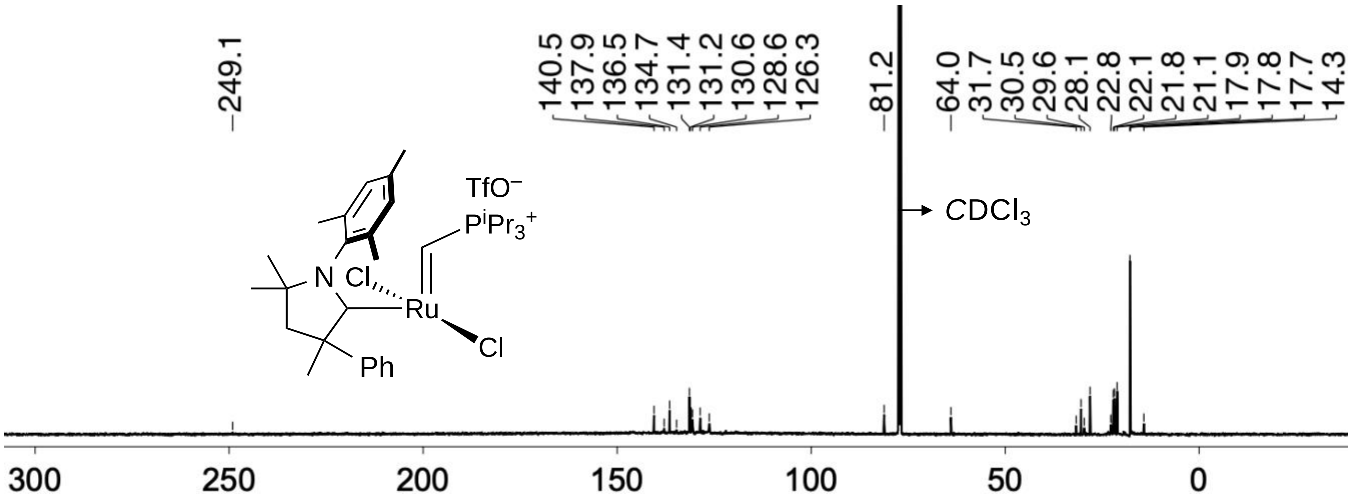

**Figure S11.** $^{13}C\{^{1}H\}$ NMR spectrum ($CDCl_3$, 125 MHz) of **PC3$^{Ph}$**.

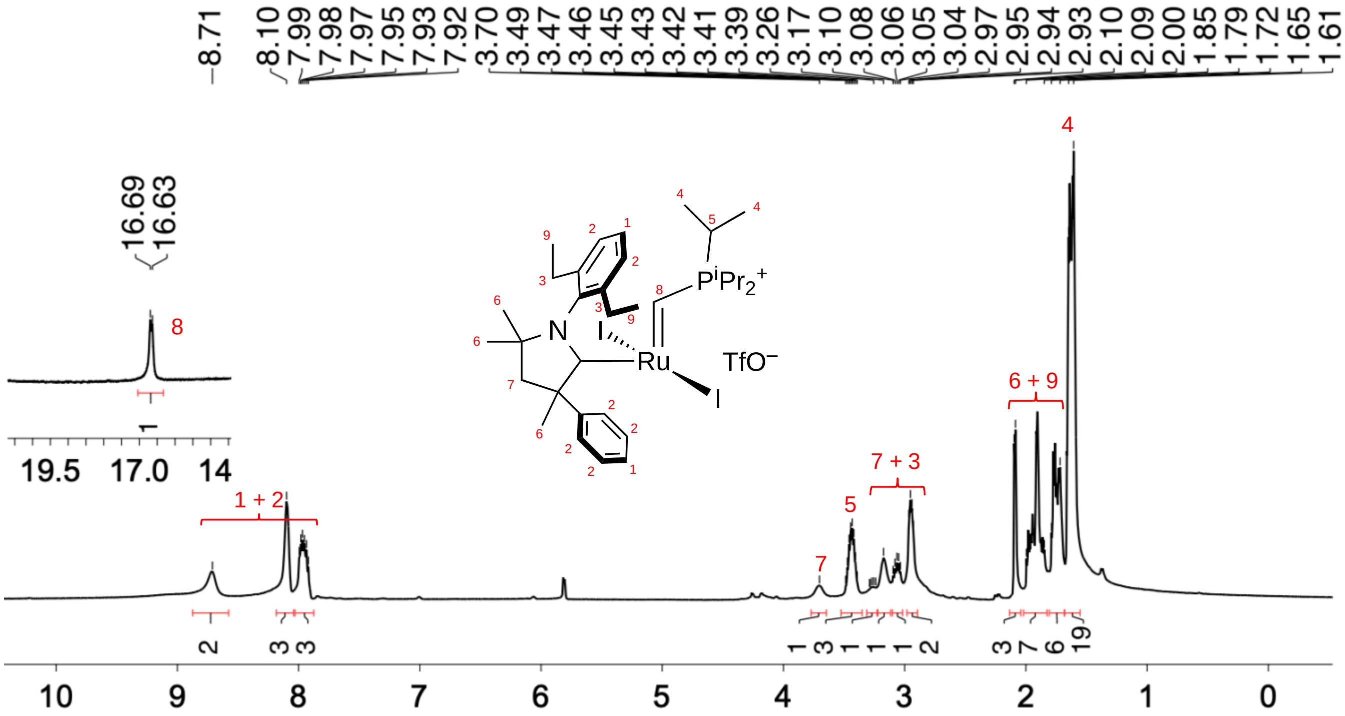

**Figure S12.** $^{1}$H NMR spectrum ($CD_2Cl_2$, 500 MHz) of **PC1$^{Ph}$(I$_2$)**.

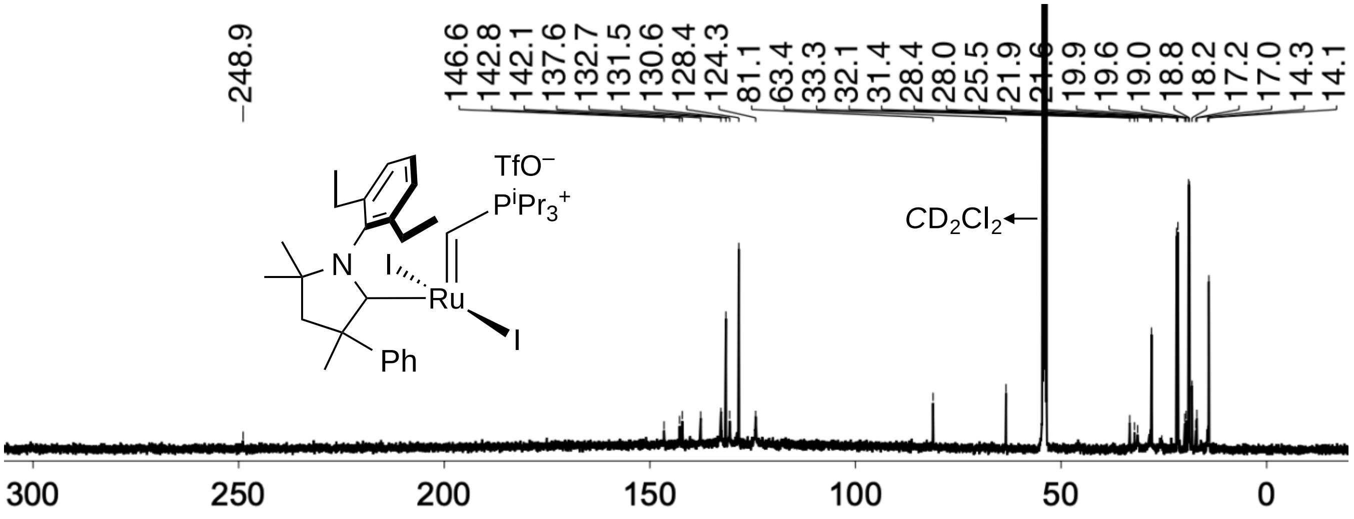

**Figure S13.** $^{13}C\{^{1}H\}$ NMR spectrum ($CD_2Cl_2$, 125 MHz) of **PC1$^{Ph}$(I$_2$)**.

**S2.3. Pyridine-stabilized methylidene complexes**

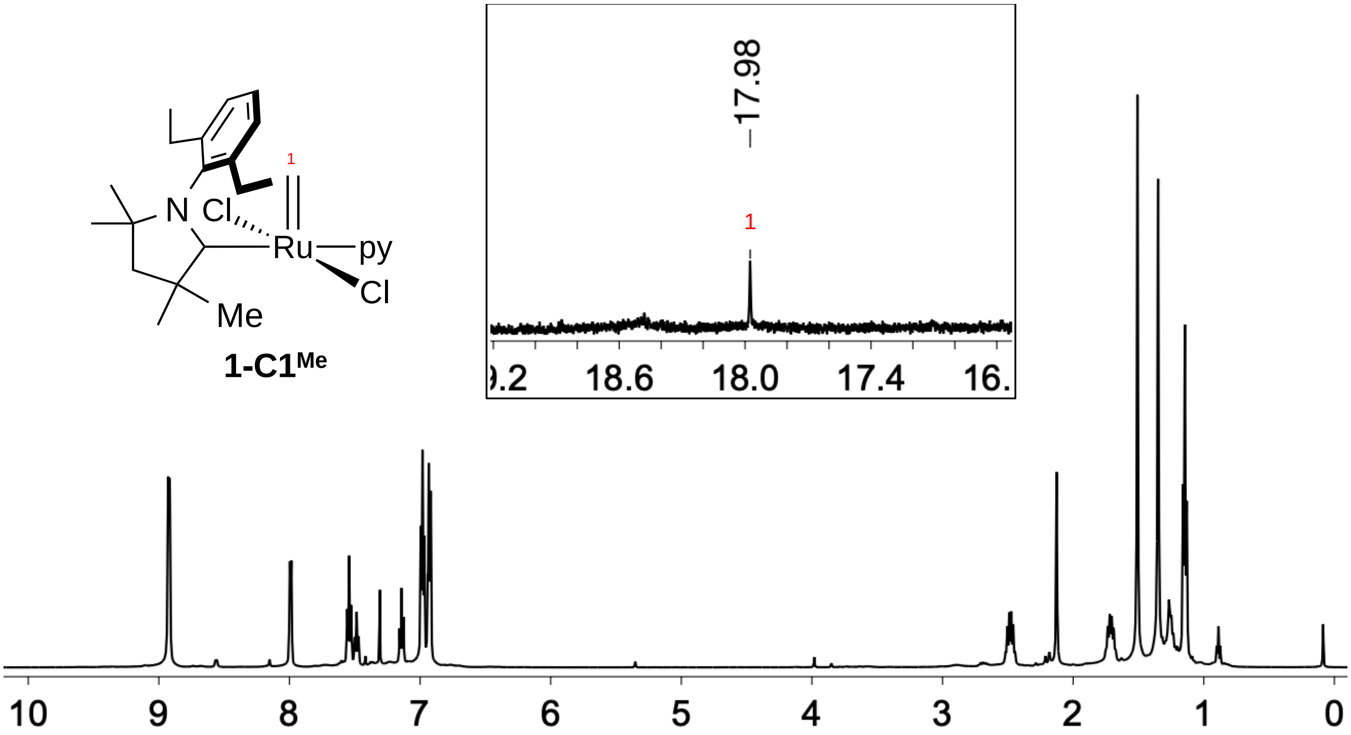


**Figure S14.** $^1$H NMR spectrum ($CDCl_3$, 500 MHz) of **1-C1$^{Me}$** at –40 °C. The downfield signal in the alkylidene region is assigned to a rotamer, as seen for other CAAC complexes.[10] The instability of the complex over 7 h at –40 °C hindered acquisition of a $^{13}C\{^1H\}$ NMR spectrum.

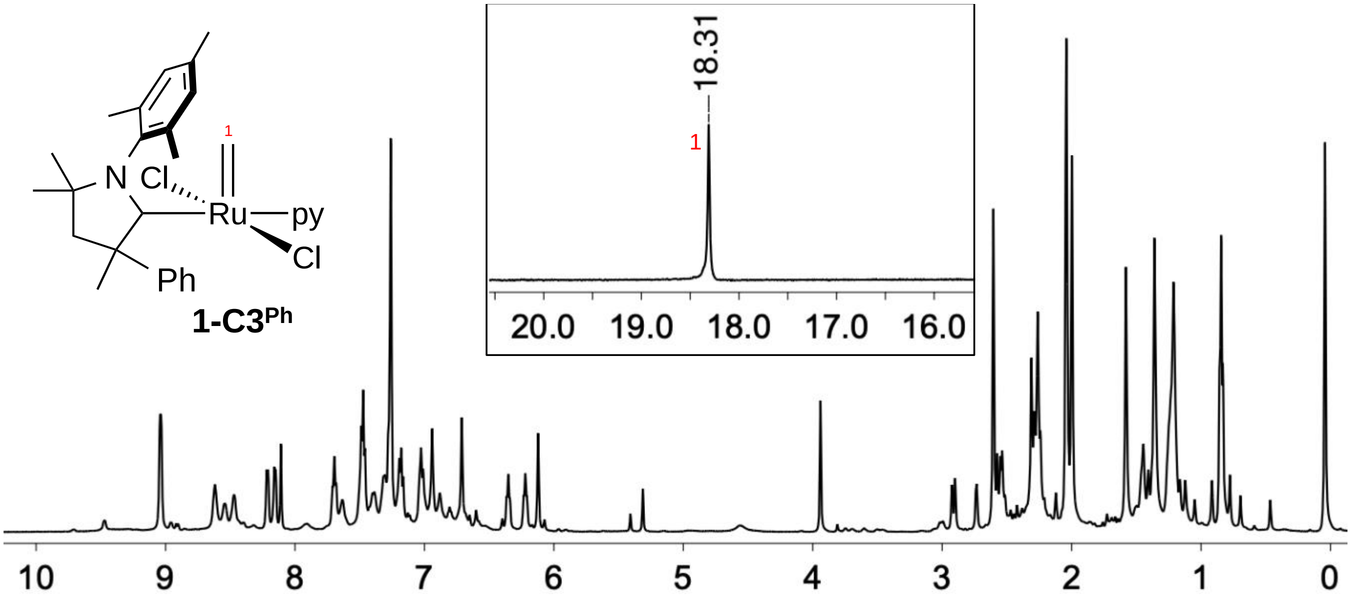


**Figure S15.** $^1$H NMR spectrum ($CDCl_3$, 500 MHz) of **1-C3$^{Ph}$** at –40 °C. The instability of the complex over 7 h at –40 °C hindered acquisition of a $^{13}C\{^1H\}$ NMR spectrum.

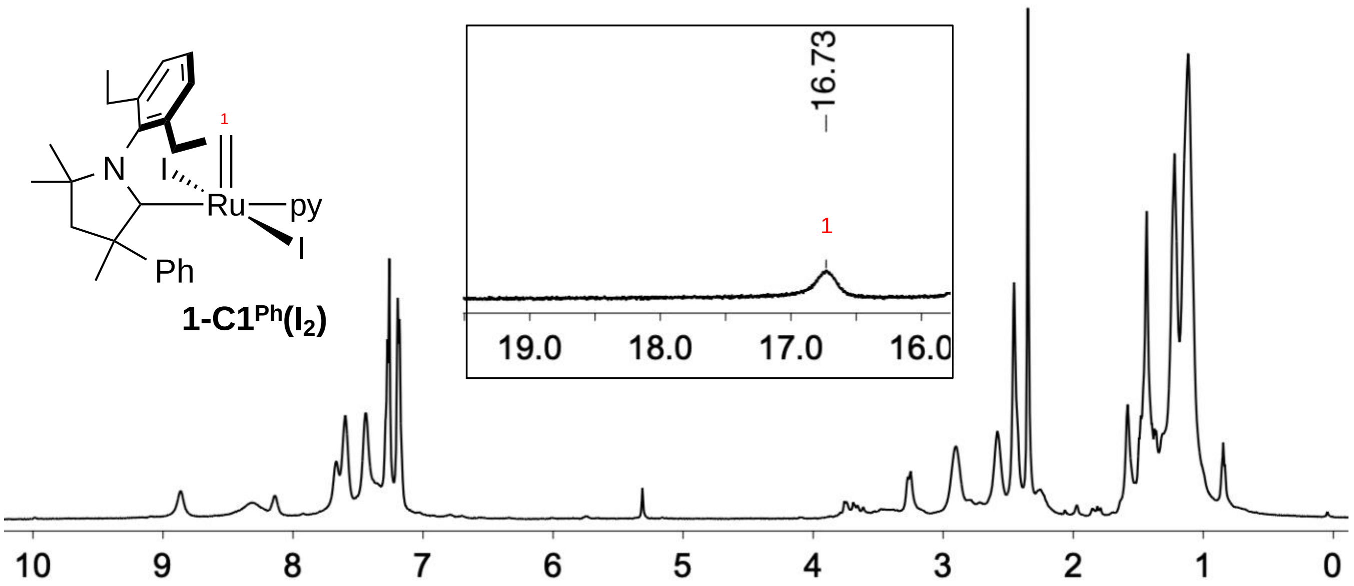


**Figure S16.** $^{1}$H NMR spectrum (CDCl$_3$, 500 MHz) of **1-C1$^{Ph}$(I$_2$)** at –40 °C.

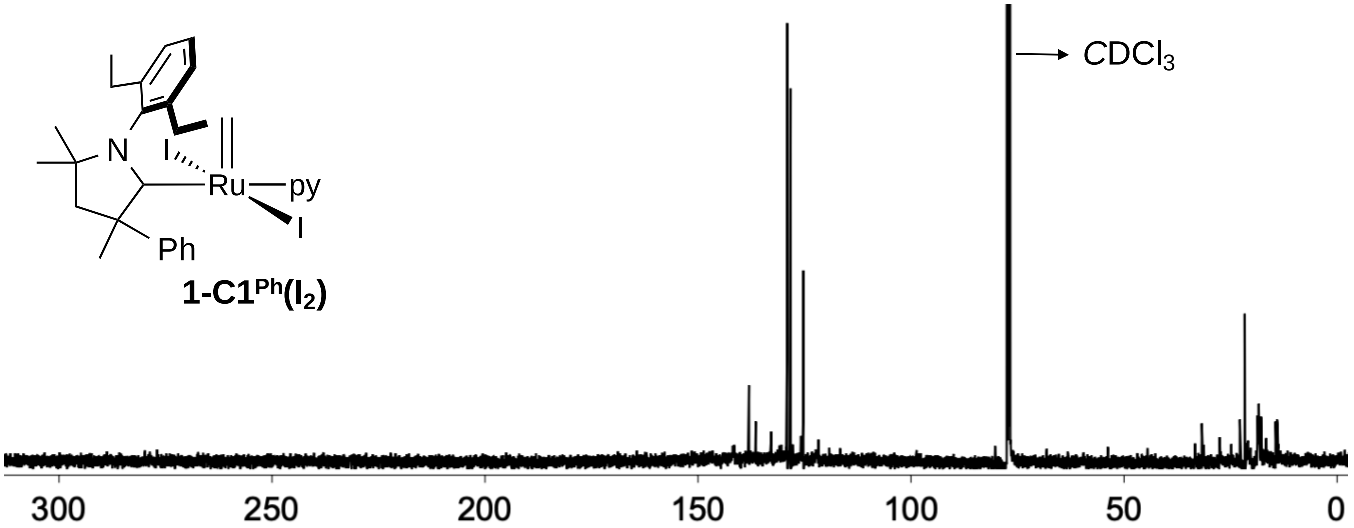


**Figure S17.** $^{13}$C{$^{1}$H} NMR spectrum (CDCl$_3$, 125 MHz) of **1-C1$^{Ph}$(I$_2$)** at –40 °C.

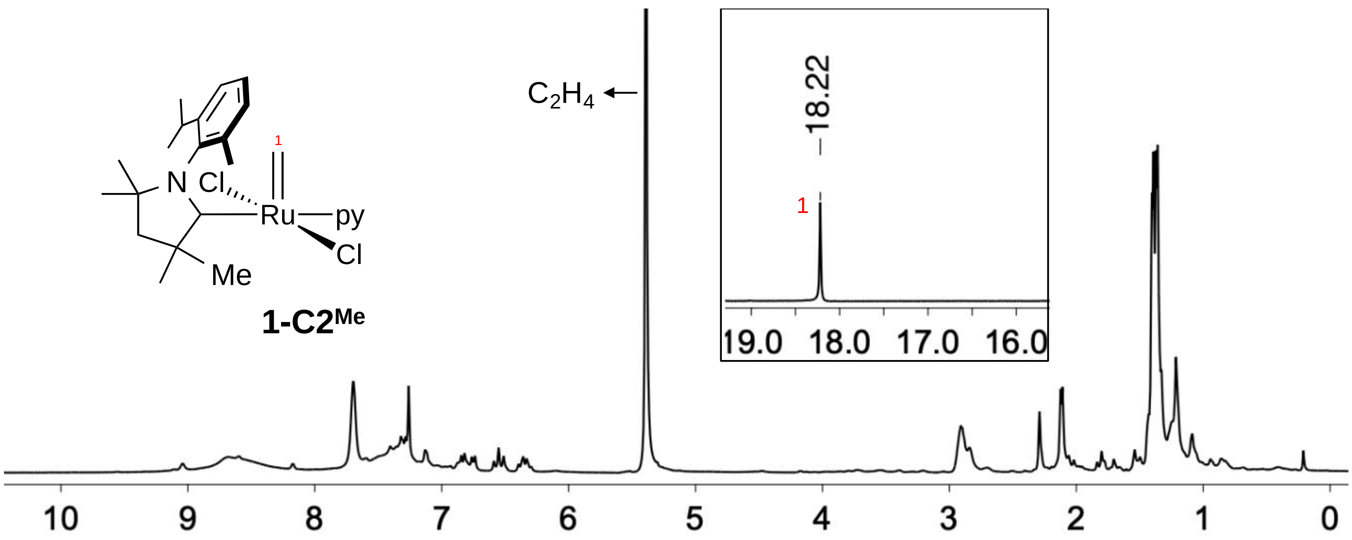

**Figure S18.** $^{1}$H NMR spectrum ($CDCl_3$, 500 MHz) showing in situ generation of **1-C2**$^{\mathbf{Me}}$ at – 40 °C.

## S3. Computational Results and Discussion

### S3.1. Mechanism of Bimolecular Coupling of Ru Methylidene

In our prior communication,[9] we briefly outlined key inferences from a computational analysis of the mechanism for bimolecular coupling: that is, interaction of two Ru-methylidene species to form a bond between the methylidene carbons, ultimately releasing ethylene. Here we present fuller details of this pathway (Figure S19, Figure S21, and Figure S22). In the discussion of the computational results, the py-stabilized methylidene species **1-H2IMes** is represented by model structure **M1**. The latter is the reference point against which relative free energies are calculated at 263 K (the temperature at which bimolecular decomposition was explored experimentally). **M1** is presumed to release pyridine to form methylidene species **M2**. These 14-electron species may increase their electronic saturation by reacting with each other to form chloride-bridged ruthenium dimers,[11,12] the nature of which is discussed below.

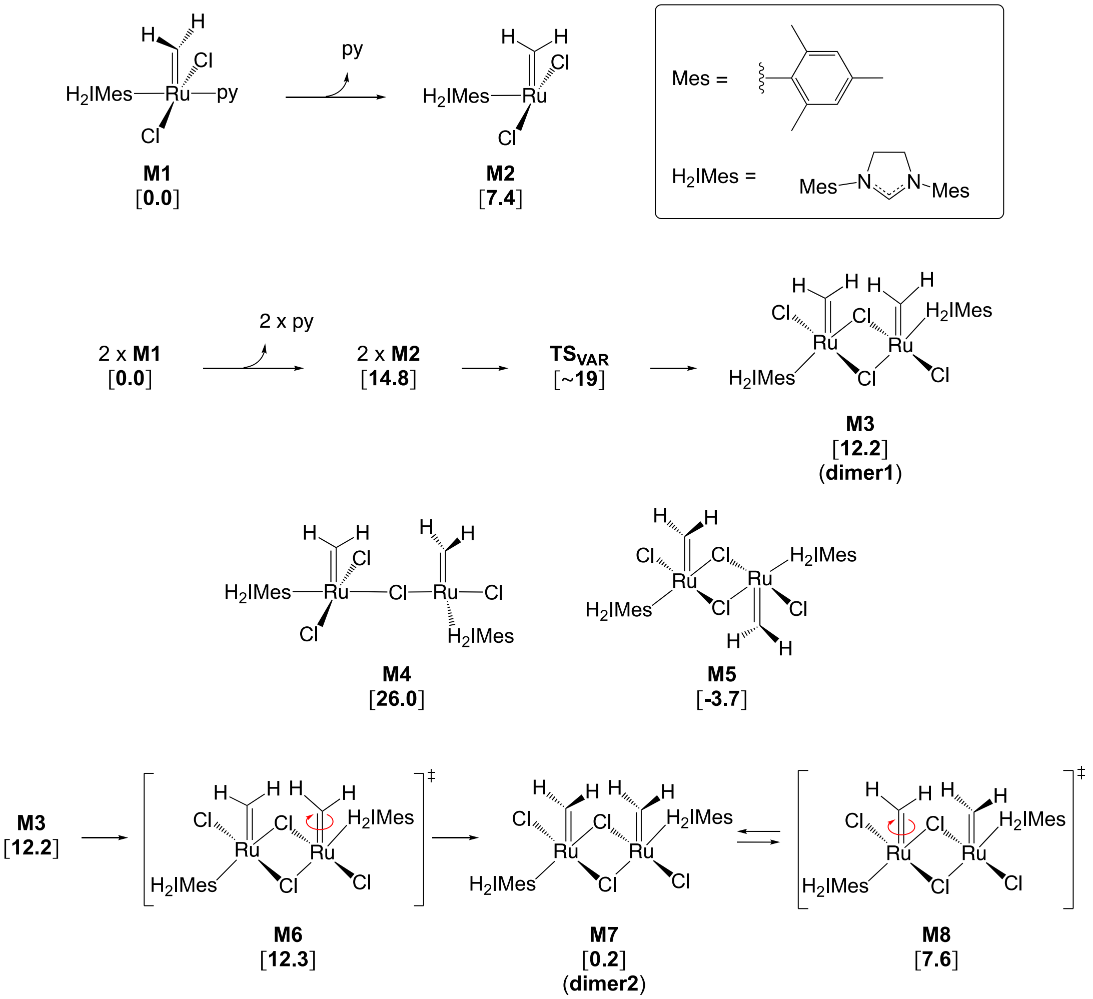


**Figure S19.** Molecular models related to formation and dimerization of **M2**. Free energies (kcal/mol, at 1 mM standard-state concentration) are reported relative to **M1**.

Whereas the methylidene unit is largely coplanar with $RuCl_2$ in **M1**, it is essentially orthogonal to this plane in the most stable conformer of 14-electron **M2**. Assuming a mechanism that is dissociative in py,[9] this conformer may dimerize with minimal conformational and geometrical changes to form **M3**. We

refer to dimers with this **M2**-like methylidene conformation as **dimer1** (see Scheme 3 in the main text). Although numerous possible mechanisms exist for dimerization, including pathways with involvement of solvent, the minimal geometrical adaption required to form **dimer1** suggests that this reaction requires negligible enthalpic activation, if any. Rather than screening a vast number of dimerization mechanisms, we estimated a lower bound for the dimerization barrier by assuming that the reaction rate is limited solely by diffusion of the monomers in solution. The free energy of the variational transition state (**TS$_{VAR}$**, at ca. 19 kcal/mol vs **M1**) for dimerization of **M2** is thus obtained by adding an estimate of the barrier to diffusion (4.4 kcal/mol, see Computational Methods) to the sum of the energies of the isolated monomers **M2** (a value of 14.8 kcal/mol vs **M1**).

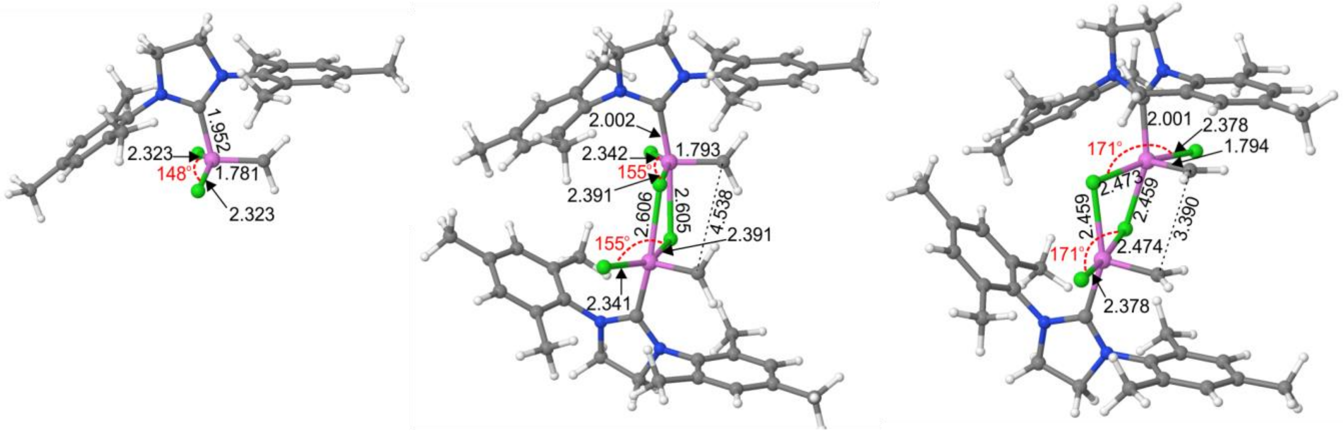


**Figure S20.** Selected geometrical parameters of the optimized geometries of **M2**, **M3** (**dimer1**), and **M7** (**dimer2**). Bond lengths are in Å and bond angle in degrees. Color coding: Ru (pink), Cl (green), C (grey), N (blue), H (white).

Whereas **M3** exhibits the syn-methylidene disposition of the $H_2C$=Ru—Ru=$CH_2$ moieties required for C–C coupling, bond formation also requires *rotation* of both methylidene units by ca. 90°, to become essentially coplanar with their respective $RuCl_2$ planes, as in **M1**.[9] We refer to this structure as **dimer2** below and in the main text. Within the $H_2$IMes system, **M7** is a model for **dimer2** and already has a C–C bond distance (3.39 Å) more than 1 Å shorter than that of **M3** (4.54 Å). **M7** is readily accessed from **M3** via transition state **M6**. The imaginary mode of **M6** is dominated by rotation of a single Ru=$CH_2$ bond, whereas the second Ru=$CH_2$ bond twists later along the minimum-energy pathway connecting **M6** and **M7**. On the latter pathway, the potential energy surface surrounding the geometry in which only a single Ru=$CH_2$ twist has occurred is quite flat. All attempts to locate a minimum in this flat region instead converged to **M7**. Similarly, rotating the Ru=$CH_2$ bond in **M7** to move backwards toward **M6** led to **M8**, the transition state for rotation of one Ru=$CH_2$ unit. Following the intrinsic reaction coordinate of **M8** along either direction led to the same basin of **M7**. In conclusion, all these explorations of the potential energy surface between **M6** and **M7** suggest that no minimum exists that corresponds to a structure with a single methylidene twisted relative to its geometry in **M3**.

In addition to **M3** and **M7**, we considered the singly-Cl bridged intermediate **M4**. This intermediate could in principle enable formation of a doubly-bridged dimer. However, its high energy excludes its involvement in the dimerization. Similarly, although known **M5** is the most stable methylidene dimer, the methylidene carbon atoms are too far apart to engage in C–C bond formation. Moreover, its Ru=C bond conformations correspond to those in **M1**, and are thus incompatible with a dimerization pathway that requires minimal geometric adaptation by monomeric **M2**. **M7**, although more stable than **M3**,

would also require considerable conformational adaption to form directly from **M2**. All these computational results, and the experimentally-observed evolution of ethylene (which implies an **M3**–**M5** equilibrium if **M5** is the primarily formed dimer), suggest that **M3**, formed by dimerization of **M2**, rearranges to **M7**, which undergoes C–C bond formation as described below.

From dimer **M7**, C–C bond formation commences with geometric distortion (via transition state **M9**) to bring the π orbital of one methylidene into the metal coordination sphere of the other Ru=$CH_2$ entity. In the resulting shallow minimum **M10**, one methylidene serves as an unusual dative ligand for the other Ru center. Close in energy is transition state **M11**, in which the bonding interaction between the two methylidene carbons first emerges. We previously described the orbital interaction involved.[9] Here we point out that the barrier to C–C bond formation from **M7** via **M11** is lower than that for sequential rotation of the Ru=$CH_2$ bonds to transform **M7** into **M3** (ca. 9 vs 12 kcal/mol, respectively), and considerably lower than the 19 kcal/mol overall barrier from pyridine adduct **M1** to **M7** via **M3** (that is, the variational transition state for dimerization of **M2**). We conclude that the rate-determining step in this pathway is dimerization, rather than formation of the C–C bond between the two methylidene units.

The first intermediate following C–C bond formation is **M12**, in which the carbon atoms are singly-bonded, as judged from the 1.55 Å C–C distance and the C-C-H bond angles (107°, 112°, 113°, 113°). The geometry suggests interaction of one $CH_2$ moiety with both Ru centers, while the other $CH_2$ moiety is bonded to only one Ru atom. The Ru atoms are only 2.5 Å apart, suggesting a metal–metal bond. Whereas electronic rearrangements leading to a spin-triplet pathway are treated below, the spin-singlet reaction pathway involves facile sliding of the **C2$^{Me}$** $H_4$ fragment to form **M14** via transition state **M13**. **M14** is best interpreted as containing an ethylene ligand (C–C 1.42 Å) $\eta^2$-coordinated to one Ru center and with a C–H agostic interaction to the second Ru atom. Breaking this agostic interaction is unexpectedly demanding, with a barrier of ca. 10 kcal/mol from **M14** via **M15**, but is facilitated by $\eta^2$-coordination of a $H_2$IMes mesityl ring trans to the agostic site. Mesityl binding, although transient, contributes to a rather substantial change in configuration at one Ru center, accounting for the significant energy barrier associated with **M15**. In the resulting structure **M16**, a conventional $\eta^2$-bound ethylene ligand (C–C 1.40 Å) is coordinated to a single Ru atom. Alternative structures bearing an ethylene ligand bound to a single Ru center (see **M18**, and **M19**) are higher in energy than **M16**, although not prohibitively so.

Finally, we modelled hypothetical spin-singlet species resulting from dissociation of ethylene. The pair of electron-deficient Ru(II) centers is likely to coordinate any electron-donating ligand, in particular the pyridine released earlier from the starting material. Moreover, especially when pyridine is not present in sufficient amounts, the Ru(II) species are expected to engage in aggregation to form Ru nanoparticles.[13]

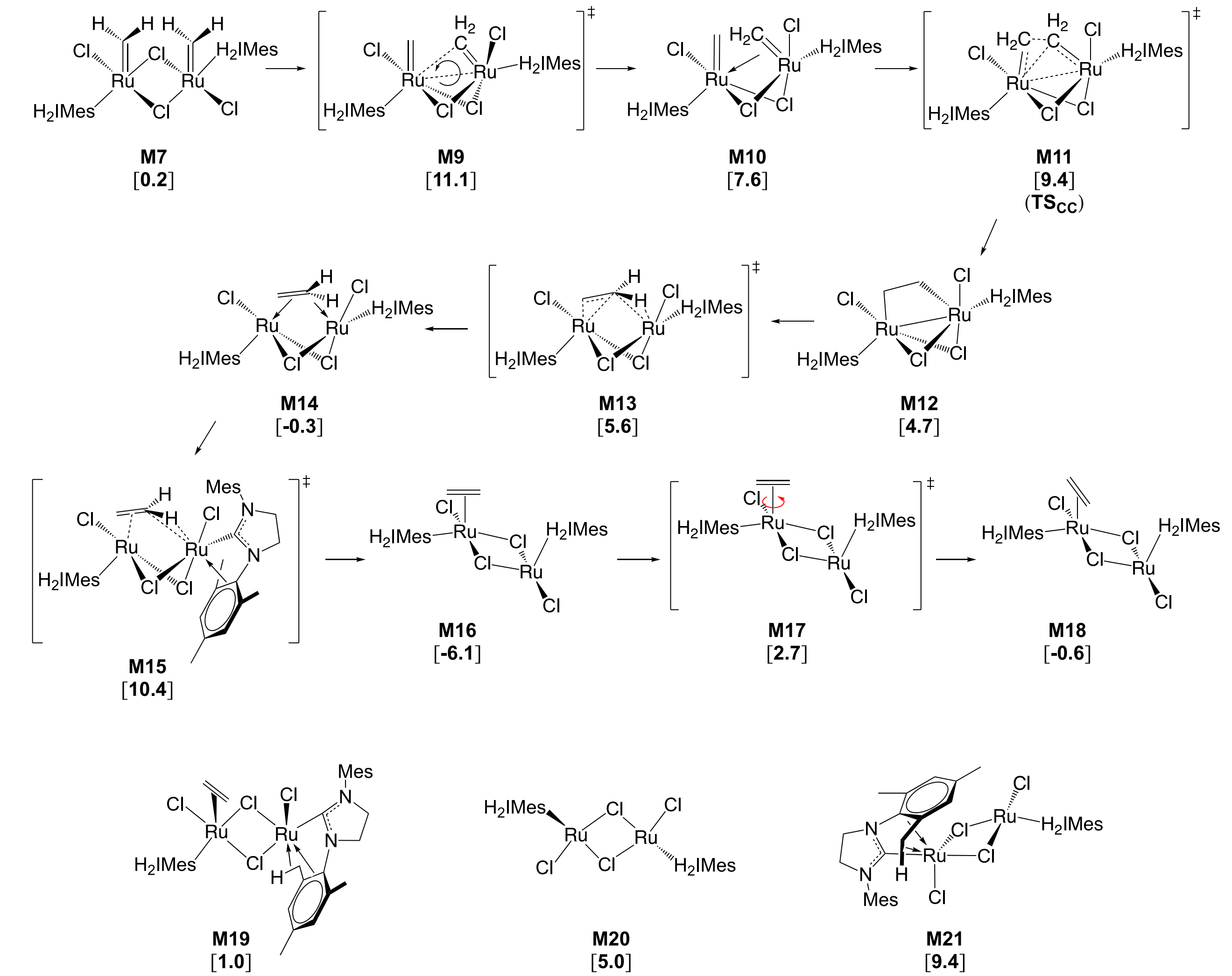


**Figure S21.** Reaction pathway for formation of ethylene from methylidenes along the spin-singlet surface. Free energy (kcal/mol, at 1 mM standard-state concentration) relative to **M1**.

As indicated above, **M12** may undergo electronic rearrangement to reach its spin-triplet counterpart $^{3}$**M23** (the superscript indicates spin multiplicity) via the minimum energy crossing point (MECP) **M22**. In $^{3}$**M23**, the two Ru centers are bridged by an ethylene moiety, in which the former methylidene carbon atoms again form a single C–C bond (1.51 Å). This structure is the lowest-energy species in the present reaction network and is thus an important and well-populated intermediate in the bimolecular decomposition pathway. Moreover, the estimated energy barrier via MECP **M22** is comparable to, or lower than, the corresponding barrier (10.4 kcal/mol, via **M15**) on the spin-singlet surface.

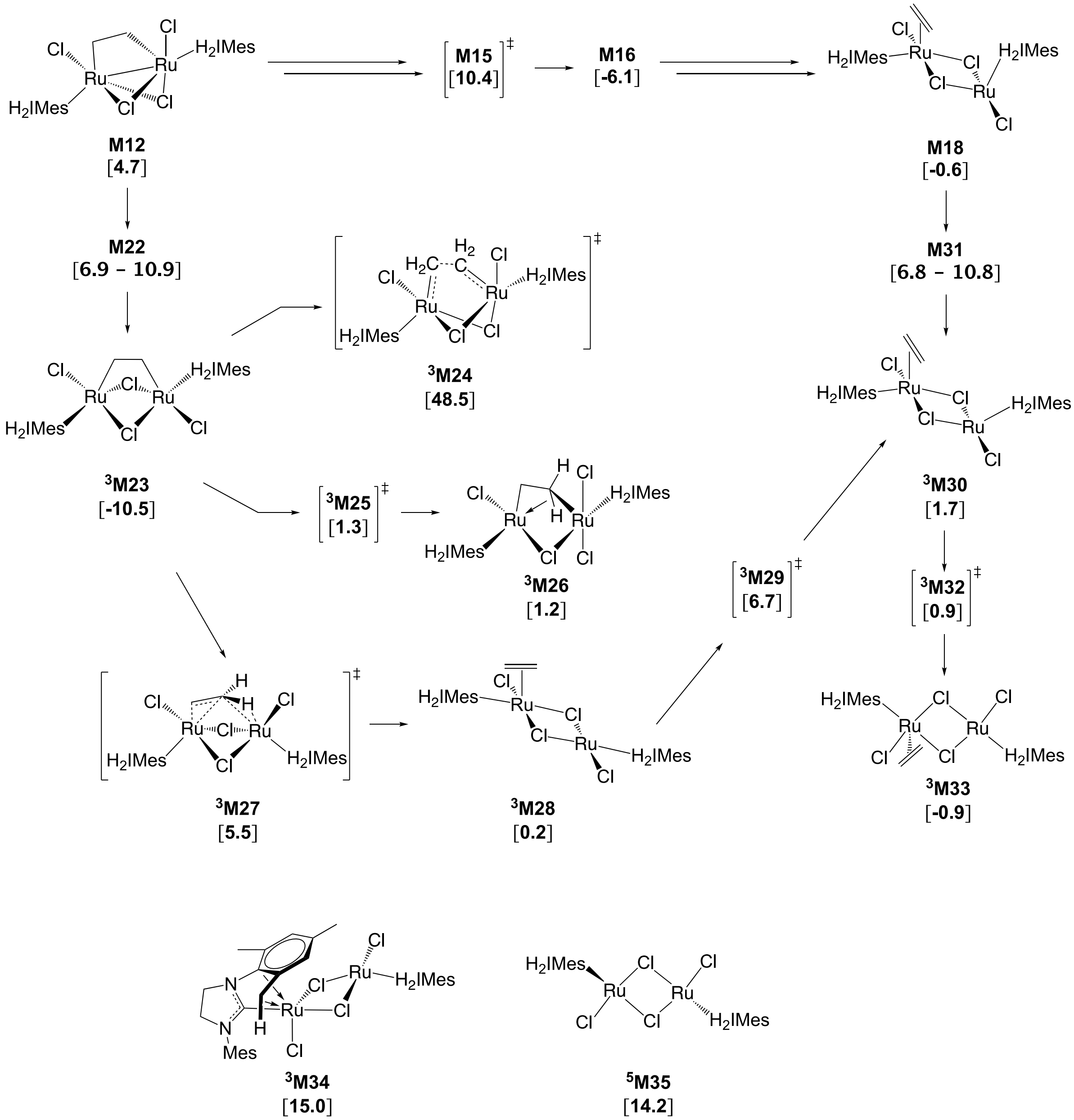


**Figure S22.** Reaction pathway for formation of ethylene along the spin-triplet surface. Free energy (kcal/mol, at 1 mM standard-state concentration) relative to **M1**. Superscript prefixes indicate spin multiplicities higher than unity.

Given the stability of $^3$**M23**, we evaluated possible reactions on the spin-triplet surface starting from this intermediate. The high-spin analogue of transition state **M11**, in which C–C bond formation starts, was ruled out on the basis of the prohibitively high energy for the relevant transition state $^3$**M24**. The least energetically demanding step from $^3$**M23** led to a C–H agostic interaction via transition state $^3$**M25**. No further energetically viable steps could be identified for evolution of the resulting species $^3$**M26**. Instead, we found, with $^3$**M27**, a step analogous to the $C_2H_4$ sliding observed at **M13** on the spin-singlet surface. However, in contrast to the spin-singlet surface, the minimum following $^3$**M27**, i.e., $^3$**M28**, does not involve C–H agostic interaction. Thus, $^3$**M28** can be viewed as the spin-triplet analogue of **M16**. Although we cannot exclude the existence of a MECP between **M16** and $^3$**M28**, the only related MECP

that could be found (i.e., **M31**) connects **M18** and $^3$**M30**. The latter intermediates are conformers of **M16** and $^3$**M28**, respectively, which differ by rotation about the Ru–ethylene bond. Notably, this ethylene twist permits access to the most stable ethylene-bound species on the spin-triplet surface, i.e., $^3$**M33**, without crossing any substantial energy barrier.

Finally, as with the spin-singlet surface, we considered the relative energy of ethylene-free intermediates of high spin. However, the energies of both the triplet $^3$**M34** and quintet $^5$**M35** are significantly higher than **M20**, their simplest counterpart on the spin-singlet surface. Ethylene dissociation may thus occur either completely on the singlet surface, or may terminate on the spin-singlet surface after undergoing spin inversion during ethylene dissociation.

The most significant barrier along the spin-triplet path is associated with rupture of the Ru–$CH_2CH_2$–Ru bridge in $^3$**M23** via $^3$**M27** (16 kcal/mol). The ensuing release of ethylene may occur from the resulting $^3$**M28** or by rearrangement via $^3$**M29** to give isomer $^3$**M33** (barrier ca. 17 kcal/mol from $^3$**M23**). Either barrier is lower than the variational estimate for dimerization of **M2** (19 kcal/mol vs **M1**). Similarly, the most demanding step after formation of the C–C bond on the spin-singlet surface is rearrangement of **M14** to yield **M16** (<12 kcal/mol from **M14**), which could already release ethylene. Overall, the barriers along both the spin-singlet and spin-triplet pathways of C–C bond formation are lower than the barrier estimated for dimerization of **M2**. Thus, irrespective of whether the ethylene-generating part of the bimolecular decomposition involves spin-singlet or spin-triplet species, the initial dimerization is predicted to be rate limiting.

### S3.2. $H_2$IMes vs C3$^{Ph}$

To limit the computational demand arising from the asymmetry of **C3$^{Ph}$**, which also involves a chiral center, we assumed that the general mechanistic features established for the $H_2$IMes catalyst would remain valid also for **C3$^{Ph}$**. Therefore, only the stationary points and elementary steps found to influence the decomposition rate of the $H_2$IMes-coordinated catalyst were investigated for the **C3$^{Ph}$**-coordinated catalyst (Figure S23 and Table S1).

First, the calculations predicted that the two rotamers of the catalyst precursor (**M36** and **M37**, in which the CAAC N-Ar moiety is syn or anti relative to the methylidene ligand, respectively) have similar solution stability. **M36** is marginally more stable, by 0.4 kcal/mol. Each rotamer exists as a racemic mixture.

Dissociation of pyridine from **M36** and **M37** leads to the 14-electron methylidene complexes (**M39** and **M38**, respectively). These are 6.2 kcal/mol and 5.7 kcal/mol less stable than **M36**, the most stable precursor rotamer. Loss of py is thus less costly with **C3$^{Ph}$** as ligand (6.2 or 5.7 kcal/mol) than with $H_2$IMes (7.4 kcal/mol). Opposite orders of stability are found for the rotamers of the 14-electron methylidene complex with respect to the catalyst precursor: The most stable rotamer for the precursor, **M36**, leads to the least stable 14-electron rotamer **M39**. The rotational barrier (via **M40**) between **M38** and **M39** is 15.9 kcal/mol vs **M36**, uphill from **M39** by only 9.7 kcal/mol. Given the very low concentration of the 14-electron species, equilibration of the rotamers **M38** and **M39** is likely to precede dimerization. The less stable **C3$^{Ph}$** rotamer in the precursor is thus probably the most abundant in the dimer, meaning that both rotamers are important for bimolecular decomposition.

For the $H_2$IMes complex, pyridine dissociation is accompanied by methylidene rotation. Whereas the methylidene unit is largely coplanar with $RuCl_2$ in **M36** and **M37**, it is essentially orthogonal to the $RuCl_2$ plane in the four 14-electron species **M38** (R' and S') and **M39** (R and S); see Figure S23 for labelling). As seen above for the $H_2$IMes catalyst (Figure S19), coupling of 14-electron methylidene

species may lead to a number of different dimers. For **C3$^{Ph}$**, we investigated only those dimers, labeled **M3** (**dimer1**) and **M7** (**dimer2**) for the $H_2$IMes catalyst, that are located along the C–C coupling pathway (Figure S23). The methylidene orientation of the 14-electron monomer is retained in **dimer1**, and dimerization is associated with minimal geometrical adaption. Assuming (as for the $H_2$IMes catalyst) that formation of this dimer is limited only by monomer diffusion in the solvent (see Section S4.4.1 The Barrier of Diffusion-Controlled Reactions), the barrier to dimerization is estimated to be 2–3 kcal/mol lower for the **C3$^{Ph}$** species than its $H_2$IMes analogue.

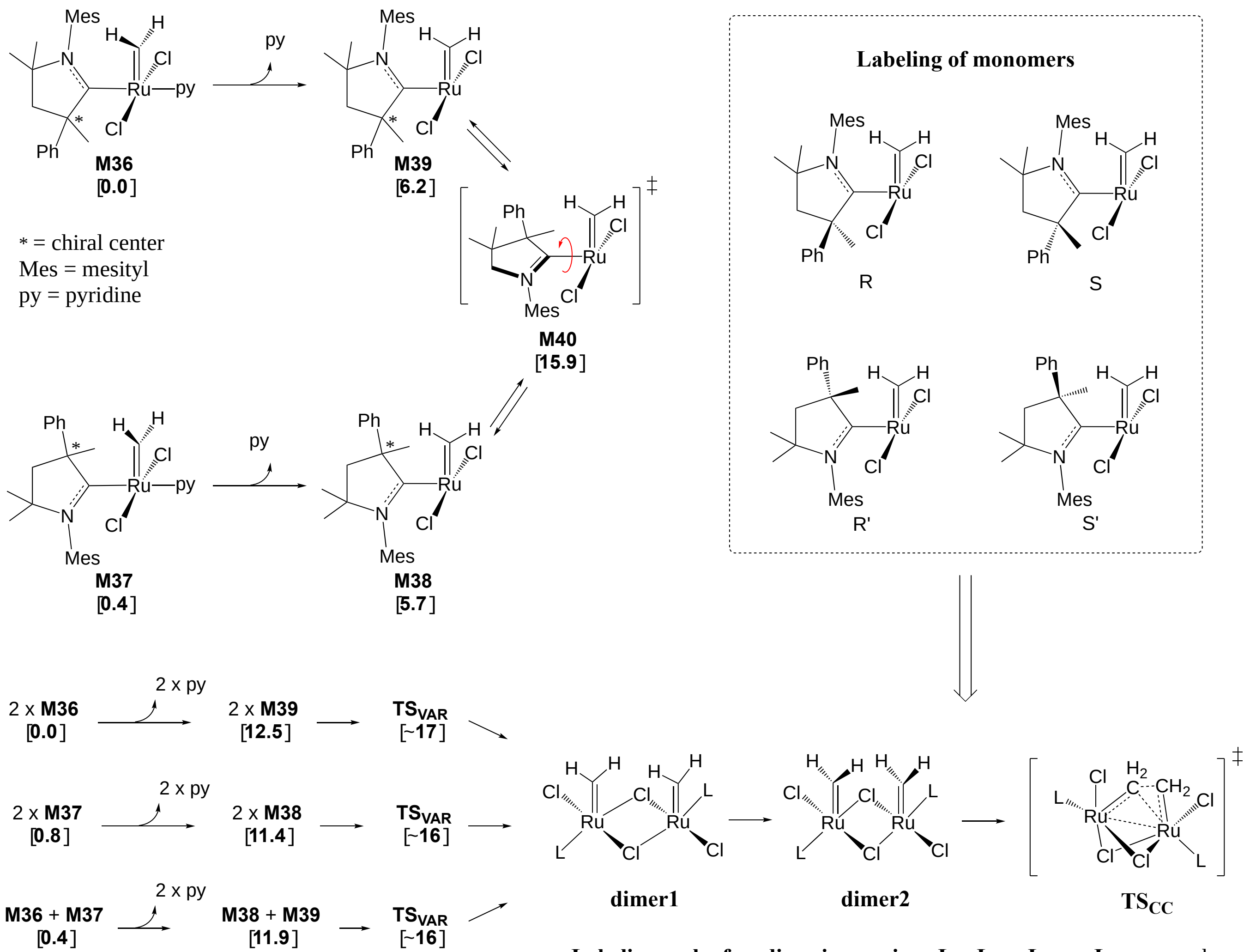


**Labeling rule for dimeric species: $L_{left}L_{right}L_{Ru\text{-}left}L_{Ru\text{-}right}$** where $\mathbf{L_{left}}$ is the label defining the left-hand side monomer, $\mathbf{L_{right}}$ is the label defining the right-hand side monomer, $\mathbf{L_{Ru\text{-}left}}$ is the configuration of the Ru atom on the left-hand side, and $\mathbf{L_{Ru\text{-}right}}$ is the configuration of the Ru atom on the right-hand side. $\mathbf{L_{Ru\text{-}left}}$ and $\mathbf{L_{Ru\text{-}right}}$ are defined in Figure S24. (e.g., RS'AA is the dimer resulting from having monomer R on the left-hand side, S' on the right-hand side, and both Ru atoms with configuration corresponding to chiral symbol A)

**Figure S23.** Stationary points and variational transition states along the bimolecular decomposition pathway of $RuCl_2$(**C3$^{Ph}$**)(py)(=$CH_2$). Free energy (kcal/mol, at 1 mM standard-state concentration) relative to **M36**.

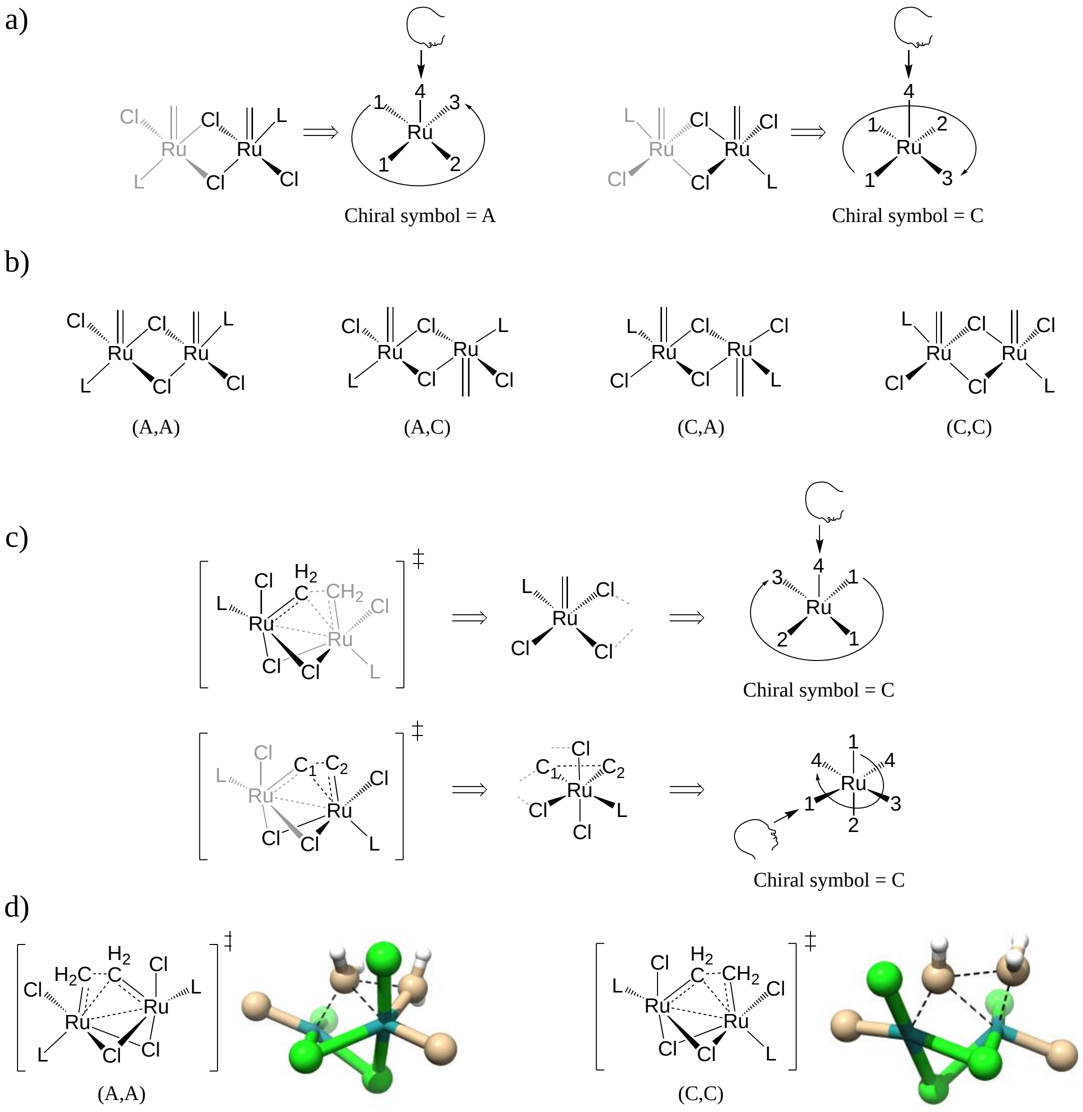


**Figure S24.** Definition of stereochemical descriptors of Ru atom in **dimer1**, **dimer2** and **$TS_{CC}$** according to IUPAC recommendations.[14] (a) Determination of chiral symbols for the square pyramidal Ru centers of **dimer1** and **dimer2**. The square pyramid is observed from the apical position toward the Ru atom, and Cahn-Ingold-Prelog priority rules (integers 1–4 represent priority 1 > 2 > 3 > 4) are applied to the substituents in the plane to determine whether the clockwise (C) or anticlockwise (A) order has highest priority. (b) Set of possible stereoisomers at the Ru centers for **dimer1** and **dimer2**. With achiral L, (A,C) and (C,A) are symmetric. (c) Determination of chiral symbols for the Ru centers in **$TS_{CC}$**. While one center is treated as a square pyramidal center (see above), the other center is approximately octahedral. The chiral symbol is defined by observing the complex along the axis involving the ligand with priority 1 (i.e., a bridging Cl) and the trans ligand with lowest priority (=$CH_2$). Clockwise (C) or anticlockwise (A) sequence of the ligands in the perpendicular plane is considered according to Cahn-Ingold-Prelog priority rules. (d) Enantiomeric core structures for **$TS_{CC}$**.

Dimerization of any pair of the four monomers R, R’, S, and S’ (nomenclature of monomers defined in Figure S23) into any of the four possible alternative stereochemistries AA, AC, CA, CC (configuration of Ru atoms in dimeric species defined in Figure S24) gives rise to 64 possible combinations. In 32 of

these combinations (involving AC and CA), the Ru=$CH_2$ moieties are mutually anti, as in **M5**, and these structures are not suitable for direct C–C bond formation. The remaining 32 consist of 16 pairs of enantiomers, six of which are redundant due to the symmetry of the dimer, leaving 10 diastereoisomeric dimers to model (see Table S1). Whereas two of these combinations (S'S'AA and RSAA, each with its degenerate stereoisomers (see Table S1) converge directly to **dimer2**, two (SS'AA and R'SAA) lead to a dimer with features intermediate between **dimer1** and **dimer2** (see Table S1 for details). The free energies of the six minima clearly corresponding to **dimer1** span over 5.8 kcal/mol (from 7.1 to 12.7 kcal/mol vs **M36**), with four being more stable than **dimer1** of the $H_2$IMes catalyst (**M3**, at 12.2 kcal/mol vs **M1**). Thus, with **C3$^{Ph}$** as ligand, some of the **dimer1** isomers form more rapidly, and are thermodynamically more stable, than those of $H_2$IMes.

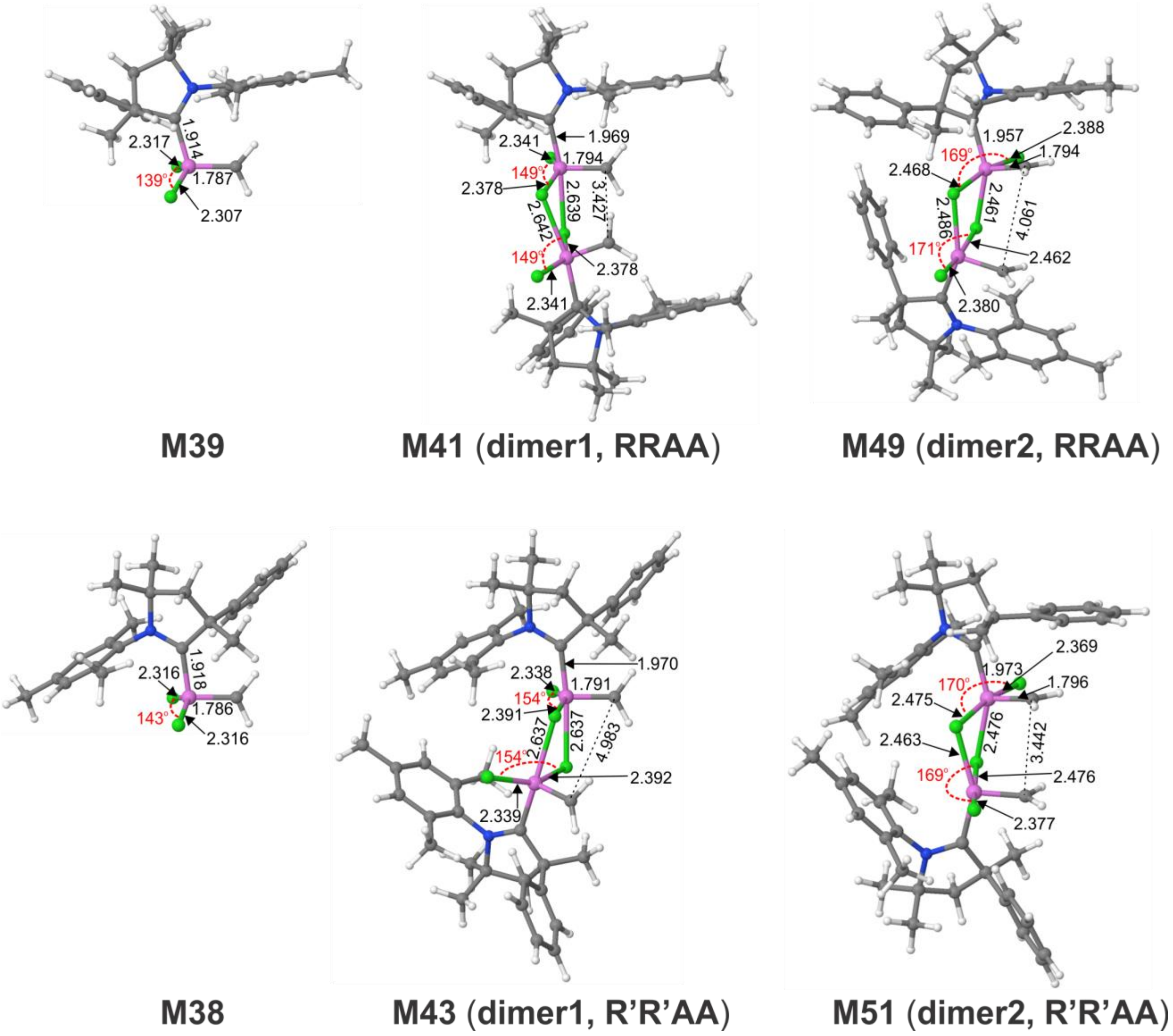


**Figure S25.** Selected geometrical parameters of the optimized geometries of **M38**, **M39**, and the corresponding dimerization products **dimer1** and **dimer2**. Bond lengths in Å, bond angles in degrees. Color coding: Ru (pink), Cl (green), C (grey), N (blue), H (white).

Nevertheless, **dimer1** is not the most stable dimer and, as for the $H_2$IMes catalyst, relaxation of **dimer1** leads to the more stable **dimer2** in which both methylidene ligands are essentially coplanar with $RuCl_2$. Except for the high-energy, symmetric combinations of R and S monomers (RR and SS), relaxation to **dimer2** leads to significant shortening of the distance between the two methylidene units. The energies of the **dimer2** complexes span 6.3 kcal/mol (from –1.9 to 4.4 kcal/mol, Table S1). With the exception of **M49** (enantiomers RRAA and SSCC), all are higher in energy relative to **M36** than the corresponding $H_2$IMes dimer **M7**, relative to **M1** (0.2 kcal/mol). Still, in all cases **dimer2** is more stable than **dimer1** and has the appropriate methylidene conformation for C–C coupling via transition state **TS**$_{CC}$.

In **TS**$_{CC}$ the two methylidene ligands have very different chemical environments, with one methylidene being weakly coordinated to the second ruthenium center (Figure S23). Therefore, whereas some stereochemical combinations were symmetrically redundant for **dimer1** and **dimer2** (e.g., RR'AA = R'RAA and RSAA = SRAA), these combinations become independent stereoisomers in **TS**$_{CC}$ (e.g., RR'AA ≠ R'RAA and RSAA ≠ SRAA). In total, 16 diastereoisomeric alternatives exist for **TS**$_{CC}$ (Table S1), spanning 8.6 kcal/mol in free energy (from 7.5 to 16.1 kcal/mol vs **M36**). For most of these isomers, C–C coupling is thus more costly than for the $H_2$IMes catalyst (with **M11** at 9.4 kcal/mol vs **M1**). However, by grouping all the alternatives for each symmetrically equivalent pair of monomers we identify six groups (**I**–**VI** in Table S1).

To illustrate, we consider the equivalence between the combination of two monomers R and that of two monomers S. Model **M59** represents **TS**$_{CC}$ with stereochemistry RRAA and its mirror image SSCC. Instead, **M63** represents **TS**$_{CC}$ with stereochemistry SSAA and its mirror image RRCC. Group **I** includes **M59** and **M63** because these identify two alternative pathways for a pair of either R or S monomers. Within this group, **M59** identifies the lowest-energy pathway. Therefore, the coupling of a pair of either R or S monomers occurs via **M59** and not via **M63**.

The lowest energy values for **TS**$_{CC}$ of each group (values underlined in Table S1) span the range 7.5–9.8 kcal/mol vs **M36**. Thus, in all cases the barrier to C–C bond formation is lower than that of the initial dimerization (**TS**$_{VAR}$ at 16–17 kcal/mol vs **M36**). The dimerization step is thus rate-determining for **C3**$^{Ph}$, as it is for $H_2$IMes. Moreover, with **TS**$_{VAR}$ at 16–17 kcal/mol vs **M36**, the dimerization step is clearly easier with **C3**$^{Ph}$ than with $H_2$IMes as ligand (**TS**$_{VAR}$ at 19 kcal/mol vs **M1**), accounting for the more rapid bimolecular decomposition observed for the **C3**$^{Ph}$ catalyst.

The above results are obtained for a standard-state concentration for all the species equal to 1 mM. However, in the kinetic experiments, the initial concentration of **1** varies: it is 1.4 mM for **1-H$_2$IMes** and 0.027 mM for **1-C3**$^{Ph}$. The concentration affects the relative energies. Table S2 displays the values for the lowest energy **TS**$_{CC}$ calculated for a standard-state concentration corresponding to the initial experimental concentration of **1**. (See Section S5, Computational Data, for the complete list of concentration-corrected energies). For $H_2$IMes, the change is small, and the barrier to dimerization ($\Delta G_2^{\ddagger}$ = 19.5 kcal/mol, see Table 1 of main text) remains clearly higher than that to CC coupling (**M11**, $\Delta G^{[1.4\ mM]}$ = 9.1 kcal/mol vs **M7**). For **C3**$^{Ph}$, the change is greater, due to the larger numerical difference between 1 mM and the experimental value of 0.027 mM. Thus, nearly all the **dimer2** species become more stable than the precursor **M36** (Table S2). Nevertheless, the energy demands of C–C coupling remain substantially lower than the barrier to dimerization ($\Delta G_2^{\ddagger}$ = 12.1 kcal/mol; see Table 1 in the main text). The latter therefore remains the rate determining step when a standard-state concentration of 0.027 mM is used for all species in the decomposition of **1-C3**$^{Ph}$.

**Table S1.** Relative Free Energies (kcal/mol) of Representative Intermediates and Transition States for C–C Bond Formation Calculated for **1-C3$^{Ph}$** at 1 mM Standard-State Concentration.

| dimer1 | | | dimer2 | | | $TS_{CC}$ | | | |
|---|---|---|---|---|---|---|---|---|---|
| Stereochemical Labels[a] | Model ID | $\Delta G^{[1\ mM]}$ [kcal/mol][b] | Stereochemical Labels[a] | Model ID | $\Delta G^{[1\ mM]}$ [kcal/mol][b] | Stereochemical Labels[a] | Group[f] | Model ID | $\Delta G^{[1\ mM]}$ [kcal/mol][b] |
| RRAA, SSCC | **M41** | 12.7 | RRAA, SSCC | **M49** | –1.9 | RRAA, SSCC | **I** | **M59** | 7.5 |
| RR'AA, SS'CC, R'RAA, S'SCC | **M42** | 10.3 | RR'AA, SS'CC, R'RAA, S'SCC | **M50** | 1.5 | RR'AA, SS'CC | **II** | **M60** | 8.3 |
| | | | | | | R'RAA, S'SCC | II | M61 | 11.7 |
| R'R'AA, S'S'CC | **M43** | 8.6 | R'R'AA, S'S'CC | **M51** | 0.7 | R'R'AA, S'S'CC | **III** | **M62** | 9.8 |
| SSAA, RRCC | M44 | 12.4 | SSAA, RRCC | M52 | 2.2 | SSAA, RRCC | I | M63 | 14.2 |
| SS'AA, RR'CC, S'SAA, R'RCC | M45[c] | 3.7 | SS'AA, RR'CC, S'SAA, R'RCC | M53 | 2.3 | SS'AA, RR'CC | II | M64 | 15.4 |
| | | | | | | S'SAA, R'RCC | II | M65 | 11.4 |
| S'S'AA, R'R'CC | -[d] | -[d] | S'S'AA, R'R'CC | M54 | 4.4 | S'S'AA, R'R'CC | III | M66 | 11.8 |
| RSAA, SRCC, SRAA, RSCC | -[d] | -[d] | RSAA, SRCC, SRAA, RSCC | **M55** | 0.3 | RSAA, SRCC | **IV** | **M67** | 8.2 |
| | | | | | | SRAA, RSCC | IV | M68 | 16.1 |
| RS'AA, SR'CC, S'RAA, R'SCC | **M46** | 10.4 | RS'AA, SR'CC, S'RAA, R'SCC | **M56** | 1.7 | RS'AA, SR'CC | V | **M69** | 9.0 |
| | | | | | | S'RAA, R'SCC | **V** | **M70** | 8.6 |
| R'SAA, S'RCC, SR'AA, RS'CC | M47[c] | 4.0 | R'SAA, S'RCC, SR'AA, RS'CC | M57 | 2.2 | R'SAA, S'RCC | V | -[e] | -[e] |
| | | | | | | SR'AA, RS'CC | V | **M72** | 14.9 |
| R'S'AA, S'R'CC, S'R'AA, R'S'CC | **M48** | 7.1 | R'S'AA, S'R'CC, S'R'AA, R'S'CC | **M58** | 2.0 | R'S'AA, S'R'CC | VI | M73 | 12.9 |
| | | | | | | S'R'AA, R'S'CC | **VI** | **M74** | 9.6 |

[a]Labeling rules defined in Figure S23. [b]Energies calculated relative to **M36**. The most favorable pathways within each group are rendered in bold face. [c]Attempts to optimize the geometry of **dimer1** gave a dimer with features intermediate between **dimer1** and **dimer2**, in which the monomer S' or R' retains the methylidene conformation present in **dimer1**, but the methylidene ligand in monomer S is rotated by ca. 90° as in **dimer2**. [d]Attempts to optimize the geometry of **dimer1** resulted in **dimer2**. [e]Despite several attempts, this stationary point could not be located. [f]Grouping based on the combination of symmetrically equivalent pairs of monomers.

**Table S2.** Relative Free Energies (kcal/mol) of Representative Intermediates and Transition States for C–C Bond Formation Calculated for **1-H2IMes** (1.4 mM) and **1-C3$^{Ph}$** (27 μM).

| **dimer1** | | | **dimer2** | | | **$TS_{CC}$** | | |
|---|---|---|---|---|---|---|---|---|
| **Label**[a] | **Model ID** | **$\Delta G^{[M]}$ [kcal/mol]**[b] | **Label**[a] | **Model ID** | **$\Delta G^{[M]}$ [kcal/mol]**[b] | **Label**[a] | **Model ID** | **$\Delta G^{[M]}$ [kcal/mol]**[b] |
| - | **M3** | 12.4 | - | **M7** | 0.4 | - | **M11** | 9.5 |
| RRAA, SSCC | **M41** | 10.8 | RRAA, SSCC | **M49** | –3.8 | RRAA, SSCC | **M59** | 5.6 |
| RR'AA, SS'CC | **M42** | 8.4 | RR'AA, SS'CC | **M50** | –0.4 | RR'AA, SS'CC | **M60** | 6.4 |
| R'R'AA, S'S'CC | **M43** | 6.7 | R'R'AA, S'S'CC | **M51** | –1.2 | R'R'AA, S'S'CC | **M62** | 7.9 |
| RSAA, SRCC | -[d] | -[d] | RSAA, SRCC | **M55** | –1.6 | RSAA, SRCC | **M67** | 6.3 |
| S'RAA, R'SCC | **M46** | 8.5 | S'RAA, R'SCC | **M56** | –0.2 | S'RAA, R'SCC | **M70** | 6.8 |
| S'R'AA, R'S'CC | **M48** | 5.2 | S'R'AA, R'S'CC | **M58** | 0.2 | S'R'AA, R'S'CC | **M74** | 7.7 |

[a]Labeling rules defined in Figure S23. [b]Energies calculated relative to **M36**. [c]Attempts to optimize the geometry of **dimer1** gave a dimer with features intermediate between **dimer1** and **dimer2**, in which the monomer S′ or R′ retains the methylidene conformation present in **dimer1**, but the methylidene ligand in monomer S is rotated by ca. 90° as in **dimer2**. [d]Attempts to optimize the geometry of **dimer1** resulted in **dimer2**. [e]Despite several attempts, this stationary point could not be located.

## S3.3. Computational Models for Catalysts Bearing Carbenes C1$^{Ph}$, C2$^{Me}$, and C1$^{Me}$

The data above for **1-H2IMes** and **1-C3$^{Ph}$** suggests that dissociation of pyridine from **1** and the ensuing dimerization of **2** are the key factors governing the rate of bimolecular decomposition. We therefore extended our computational investigation to include the pyridine-bound catalysts **1** and the 14-electron complexes **2** for the other CAAC catalysts, i.e., **1-C1$^{Ph}$** (Figure S26), **1-C1$^{Ph}$(I$_2$)** (Figure S27), **1-C2$^{Me}$** (Figure S28), and **1-C1$^{Me}$** (Figure S29).

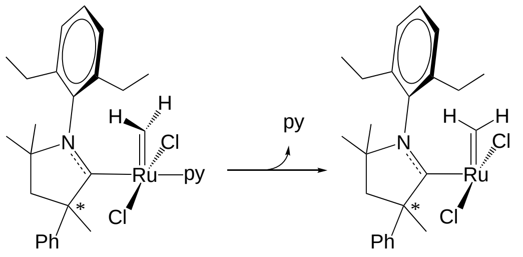


**M75** [**0.0**] → **M77** [**5.4**]

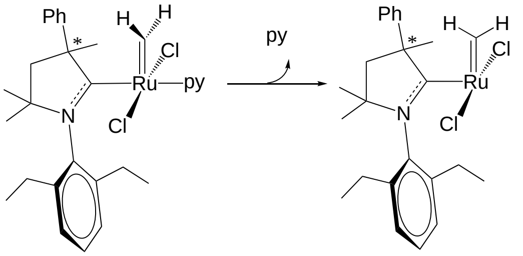


**M76** [**1.6**] → **M78** [**5.7**]

**Figure S26.** Computational investigation of pyridine dissociation from **1** to give **2** for **1-C1**$^{\mathbf{Ph}}$. Free energies (kcal/mol, at 1 mM standard-state concentration) calculated relative to **M75**.

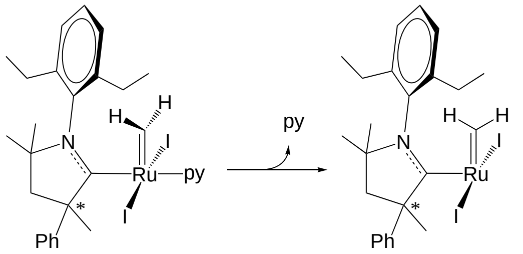


**M87** [**0.0**] → **M89** [**4.7**]

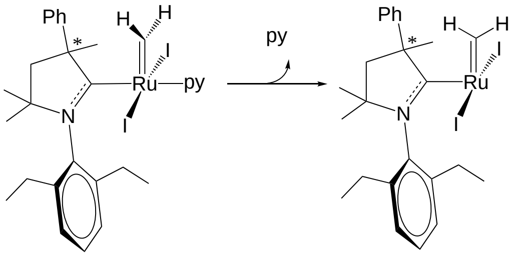


**M88** [**3.4**] → **M90** [**6.7**]

**Figure S27.** Computational investigation of pyridine dissociation from **1** to give **2** for **1-C1**$^{\mathbf{Ph}}$**(I**$_2$**)**. Free energies (kcal/mol, at 1 mM standard-state concentration) calculated relative to **M87**.

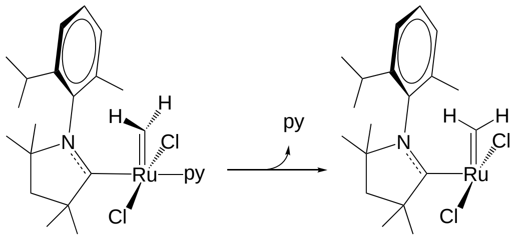


**M79** [**0.0**]　　**M81** [**0.4**]

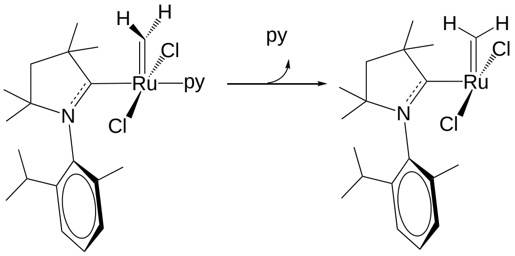


**M80** [**1.5**]　　**M82** [**3.7**]

**Figure S28.** Computational investigation of pyridine dissociation from **1** to give **2** for **1-C2$^{Me}$**. Free energies (kcal/mol, at 1 mM standard-state concentration) calculated relative to **M79**.

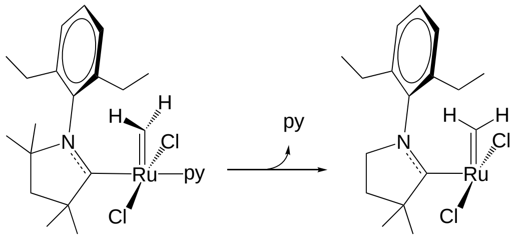


**M83** [**0.0**]　　**M85** [**2.8**]

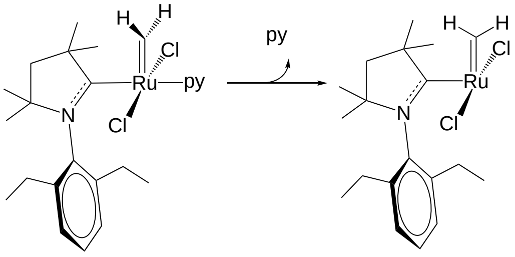


**M84** [**2.0**]　　**M86** [**7.0**]

**Figure S29.** Computational investigation of pyridine dissociation from **1** to give **2** for **1-C1$^{Me}$**. Free energies (kcal/mol, at 1 mM standard-state concentration) calculated relative to **M83**.

**Table S3.** Selected Interaction Energies E(2) from Second-Order Perturbation Theory Analysis of the Fock Matrix in NBO Basis.[a]

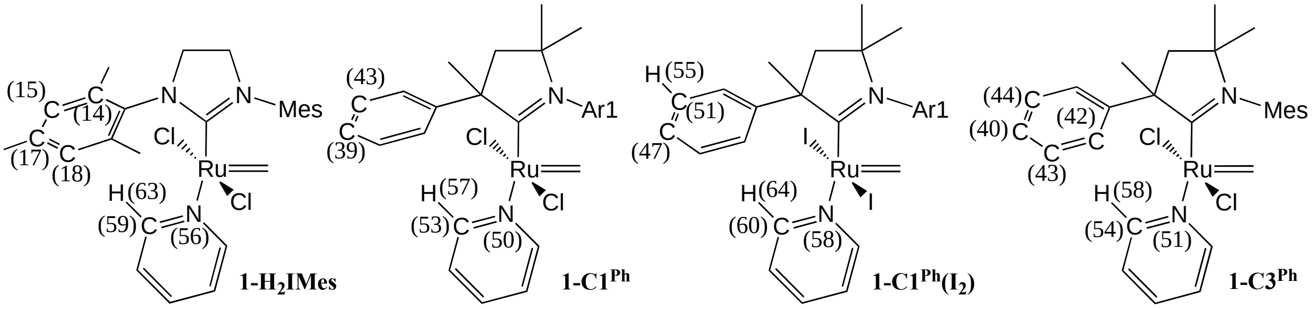


Mes = mesityl, Ar1 = 2,6-diethylphenyl

| Complex | Donor NBO | Acceptor NBO | E(2) (kcal/mol) |
|---|---|---|---|
| **1-H$_2$IMes** | BD ( 2) C 17- C 18 | BD*( 2) N 56- C 59 | 0.38 |
| **1-C1$^{Ph}$** | BD ( 2) C 39- C 43 | BD*( 2) N 50- C 53 | 0.70 |
| **1-C1$^{Ph}$(I$_2$)** | BD ( 2) C 47- C 51 | BD*( 1) C 60- H 64 | 0.45 |
| **1-C3$^{Ph}$** | BD ( 2) C 40- C 44 | BD*( 2) N 51- C 54 | 0.75 |

[a] Interaction energies <0.3 kcal/mol not shown.

## S4. Computational Methods

Construction, analysis, and visualization of molecular structures were carried out using the following software packages: ChemAxon's Marvin (version 6.2.1),[15] UCSF Chimera (version 1.14),[16] and Molden (version 5.0).[17] Conformational searches and preliminary strain relaxations of molecular models were performed in Spartan18 using the Merck force field (MMFF94)[18] and the semi-empirical PM6 methods[19]. As such empirical and semi-empirical methods are generally less accurate for transition-metal chemistry than for organic chemistry, internal coordinates, such as metal-ligand bond distances and angles, describing the immediate metal coordination environment, were frozen in these preliminary calculations. All density functional theory (DFT) calculations were performed with the Gaussian suite of programs, versions 09 D.01[20] and 16 C.01.[21]

### S4.1. Location of Stationary Points and Minimum Energy Crossing Points (MECPs)

Molecular geometries were optimized using Head-Gordon's long-range- and dispersion-corrected hybrid density functional ωB97XD functional.[22] This functional produces geometries in good agreement with those from X-ray diffraction for ruthenium catalysts for olefin metathesis and other homogeneous catalysts.[23] All elements except ruthenium were described with Dunning's correlation-consistent valence double-ζ plus polarization basis sets (cc-pVDZ)[24,25] as obtained from the EMSL basis set exchange database.[26,27] Ruthenium atoms were described by combining the Stuttgart 28-electron relativistic effective core potential (termed ECP28MDF[28] at the Stuttgart/Cologne Group website)[29] with the correlation-consistent valence double-ζ plus polarization basis set (cc-pVDZ-PP)[28] obtained from the EMSL basis set exchange database.[26,27] Numerical integration was performed using Gaussian's "ultrafine" grid. Wavefunctions were converged using the default convergence criteria for self-consistent field (SCF) procedures (RMS change in density matrix < $1.0 \cdot 10^{-8}$, max. change in density matrix = $1.0 \cdot 10^{-6}$), which correspond to the SCF option conver=8. All SCF solutions were checked for internal instability before optimizing geometry. Unstable solutions were re-optimized to a real, spin-restricted or unrestricted solution for spin-singlet or higher multiplets, respectively.

Next, geometries were optimized without symmetry constraints using tight convergence criteria (i.e., opt=tight, corresponding to max. force $1.5 \cdot 10^{-5}$, RMS force $1.0 \cdot 10^{-5}$, max. displacement $6.0 \cdot 10^{-5}$, RMS displacement $4.0 \cdot 10^{-5}$). In a few cases, the SCF convergence criteria were tightened tenfold compared to the above-described default (i.e., conver=9 was used instead of conver=8) to speed up geometry optimization. In all these cases, the resulting geometry was re-optimized with the default SCF convergence criteria. All reported geometries have thus been obtained with the default SCF convergence criteria (conver=8).

Minimum energy crossing points (MECPs) between spin-singlet and spin-triplet surfaces were located using Gaussian 09.d01 in conjunction with via the seam-of-crossing optimizer developed by Harvey (version: November-2009).[30] The DFT model, basis sets, and options of these calculations were identical to those of the regular geometry optimizations described above. To convergence criteria for the seam-of-crossing optimizations were as follows: The maximum allowed electronic energy gradient was $1.0 \cdot 10^{-4}$ a.u., and the maximum allowed energy difference between the two spin-states was 0.00050 a.u. The curvature along the seam was confirmed by the eigenvalues of state-averaged Hessian matrix obtained using the Glowfreq program (version November-2015).[31]

The input files of the geometry optimizations and Hessian calculations are available in the ioChem-BD repository.[32,33] at https://doi.org/10.19061/iochem-bd-6-79.

## S4.2. Hessian Calculations and Thermochemical Corrections

All located stationary points were subjected to analytical calculation of the second derivatives, i.e., the Hessian matrix. Except for the integration grid being reduced from "ultrafine" to "finegrid" in these coupled perturbed Hartree-Fock (CPHF) calculations, the basis sets and options of these Hessian calculations were identical to those of the geometry optimizations. The eigenvalues of the analytically calculated Hessian matrices were used to characterize each stationary point as either a minimum (positive eigenvalues only) or a transition state (a single negative eigenvalue) on the potential energy surface. The translational, rotational, and vibrational components of the thermal corrections to enthalpies and Gibbs free energies were calculated within the ideal-gas, rigid-rotor, and harmonic oscillator approximations (temperature = 263 K), except that all frequencies below 100 $cm^{-1}$ were shifted to 100 $cm^{-1}$ when calculating the vibrational component of entropy (i.e., quasi-harmonic oscillator approximation).[34,35]

## S4.3. Single-Point Energy Calculations

The energy of all stationary points and MECPs was calculated with the PBE functional developed by Perdew, Burke and Ernzerhof[36,37] in combination with Grimme's D3 empirical dispersion term with Becke-Johnson damping (GD3BJ).[38] When corrected for basis set superposition errors (BSSE), this combination of functional and empirical dispersion was found to be the most accurate in a validation study of density functionals for ruthenium-catalyzed olefin metathesis, reproducing mass-spectrometry-determined relative energies of olefin metathesis reaction steps in the gas phase with absolute errors close to 2 kcal/mol.[39] The PBE-D3BJ combination is expected to perform well also in the present study of ruthenium-mediated reactions related to olefin metathesis, and the large single-point (SP) basis sets (see below) should eliminate the need for BSSE corrections and contribute to accurate relative energies, with errors expected to remain within 2–5 kcal/mol. The BJ damping parameters used were those of Smith and co-workers, which were shown to increase the accuracy substantially compared to the original parameters.[40] Electrostatic and non-electrostatic solvation effects in $CHCl_3$ were accounted for using the polarizable continuum model (PCM) in combination with the "Dis", "Rep", and "Cav" keywords.[41-43] The solvent ($CHCl_3$) was characterized by the built-in value for the dielectric constant, and the solute cavity was constructed using the united atom topological model with atomic radii optimized for Hartree-Fock (keyword "UAHF"). Ruthenium was described by the ECP28MDF relativistic effective core potential[28] accompanied by a correlation-consistent valence quadruple-ζ plus polarization basis set (ECP28MDF_VQZ),[28] both obtained from the Stuttgart/Cologne Group website.[29] Carbon and hydrogen atoms were described by valence quadruple-ζ plus polarization (cc-pVQZ)[24,25] as obtained from the EMSL basis set exchange database.[26,27] For all other atoms, augmented valence quadruple-ζ plus polarization (aug-cc-pVQZ)[24,25,44] basis sets were taken from the EMSL repository.[26,27]

Finally, numerical integrations were performed with the "ultrafine" grid of Gaussian 16. The density-based SCF convergence criteria were $1.0 \cdot 10^{-5}$ (RMS change) and $1.0 \cdot 10^{-3}$ (maximum change), respectively, corresponding to the SCF option conver=5.

Input files are available in the ioChem-BD repository.[32,33] at https://doi.org/10.19061/iochem-bd-6-79.

## S4.4. Calculation of Gibbs Free Energies

Gibbs free energies were calculated at 263 K according to Equation S6:

$$G^{[M]} = E^{CHCl_3} + \Delta G_{wB97XD,qh}^{T=263\,K} + \Delta G_{1atm\to[M]}^{T=263\,K}, \qquad \text{Equation S6}$$

where $E^{CHCl_3}$ is the energy resulting from single-point calculation, including solvent effects (from the implicit solvation model) and the dispersion correction, $\Delta G_{wB97XD,qh}^{T=263\,K}$ is the thermal correction to the Gibbs free energy calculated at the geometry optimization level using the quasi-harmonic approximation, and $\Delta G_{1atm\to[M]}^{T=263\,K}$ is the standard-state correction corresponding to a solution (but exhibiting infinite-dilution, ideal-gas-like behavior) with a given concentration ([M]). The concentration is set to either 1 mM for general-purpose data that need to be comparable across different species, or to the experimental concentration used in the kinetic experiments, namely, 1.4 mM for computational data referring to experiments with **1-H$_2$IMes**, 61 μM for **1-C1$^{Ph}$**, 586 μM for **1-C1$^{Ph}$(I$_2$)**, 10 μM for **1-C2$^{Me}$**, 10 μM for **1-C1$^{Me}$**, and 27 μM for **1-C3$^{Ph}$**. Free energy values calculated with one or the other concentration are clearly identified by table/figure captions and footnotes.

All values are collected in Table S4, Table S6, Table S7, Table S8, Table S9, and Table S10 of the Computational Data section.

### S4.4.1. The Barrier of Diffusion-Controlled Reactions

Dissociative elementary reaction steps that require small or no geometrical strain sometimes do not involve enthalpic barriers on the potential energy surface. In such cases, attempts to explore the dissociation of the two fragments typically lead to minimum energy pathways with monotonically increasing potential energy, with the barrier corresponding to complete separation of the two fragments. However, even without a maximum on the potential energy surface, the corresponding free energy surface in solution may still be associated with a maximum corresponding to a variational transition state. The free energy of the latter can be estimated by reversing the Eyring equation using a typical rate constant ($4\cdot10^9$ s$^{-1}$ in common organic solvents) for diffusion-controlled reactions in usual conditions.[45] The associated free-energy barrier (4.4 kcal/mol) is calculated by reversing the Eyring equation (using 298 K to account for the fact that the above typical value of the reaction rate refers to usual conditions, thus room temperature rather than –10°C).[46] Finally, the estimated free energy for the variational transition state (labeled **TS$_{VAR}$**) of dimerization is obtained by adding the above correction to the energy of the two isolated monomers.

### S4.4.2. The Barrier Associated with Spin Inversion

The free energy of MECPs was calculated as follows: i) The single-point energy was taken to be the average of the single-point energies obtained for the two spin multiplicities at the MECP geometry, ii) the thermochemical corrections were taken to be the average obtained for the minima on either side of the MECP on the seam of crossing, and, iii) an additional penalty ($\Delta G_{pXover}$), suggested by Schneider and co-workers to be in the range 1–5 kcal/mol,[47] was added to account for a lower-than-unity spin-crossing probability.[48]

$$G^{[M]} = \bar{E}^{CHCl_3} + \overline{\Delta G}_{wB97XD,qh}^{T=263\,K} + \Delta G_{pXover} + \Delta G_{1atm\to[M]}^{T=263\,K}, \qquad \text{Equation S7}$$

where

$$\bar{E}^{CHCl_3} = \frac{E(S=0)^{CHCl_3} + E(S=1)^{CHCl_3}}{2},$$ Equation S8

and where $E(S=0)^{CHCl_3}$ and $E(S=1)^{CHCl_3}$ are the single-point energies calculated for the MECP geometry with spin-singlet and triplet-state multiplicity, respectively, and

$$\overline{\Delta G}_{wB97XD,qh}^{T=263\,K} = \frac{\Delta G(opt,\ S=0)_{wB97XD,qh}^{T=263\,K} + \Delta G(opt, S=1)_{wB97XD,qh}^{T=263\,K}}{2}$$ Equation S9

where $\Delta G(opt,\ S=0)_{wB97XD,qh}^{T=263\,K}$ and $\Delta G(opt, S=1)_{wB97XD,qh}^{T=263\,K}$ are the thermal corrections to the Gibbs free energy calculated at the geometry-optimization level with the quasi-harmonic approximation for the neighboring minima on the spin-singlet and spin-triplet surface, respectively. The resulting values are collected in Table S5.

## S4.5. Natural Bond Order and Partial Charge Analysis

The natural bond orbital analyses were performed with the NBO7 software,[49] using the electron density of the single-point energy calculations as input. To obtain a comparable set of orbitals among the complexes, Lewis structures were explicitly given via the $CHOOSE input section.

## S4.6. Calculations of Buried Volume

Buried volumes (the percentage of a sphere that is occupied, $\%V_{bur}$) for the DFT-optimized pyridine-free, 14-electron active complexes **2**, including hydrogen atoms, were obtained using the SambVca 2.1 web application developed by Cavallo and co-workers.[50] A sphere of radius 3.5 Å centered on ruthenium was used, and the van der Waals radii were those of Bondi,[51] scaled by 1.17. The mesh spacing for numerical integration was 0.10 Å.

## S5. Computational Data

A data set collection of computational results is available in the ioChem-BD repository[32] and can be accessed via https://doi.org/10.19061/iochem-bd-6-79.

**Table S4.** Free Energies (a.u.) and Relative Free Energies (kcal/mol) of Minima and Transition States of Complexes Bearing an $H_2$IMes Ligand.

| Model ID | Spin multiplicity | $\Delta G^{T=263\,K}_{wB97XD,qh}$ [a.u.] | $G^{[1\,mM]}$ [a.u.] | $\Delta G^{[1\,mM]}$ [kcal/mol][a] | $G^{[1.4\,mM]}$ [a.u.] | $\Delta G^{[1.4\,mM]}$ [kcal/mol][a] |
|---|---|---|---|---|---|---|
| **pyridine** | 1 | 0.065840 | -248.030422 | - | - | - |
| **ethylene** | 1 | 0.032032 | -78.479204 | - | - | - |
| **M1** | 1 | 0.495474 | -2226.414704 | 0.0 | -2226.414424 | 0.0 |
| **M2** | 1 | 0.406017 | -1978.372526 | 7.4 | -1978.372246 | 7.6 |
| 2 x **M2** | 1 | 0.406017 | -1978.372526 | 14.8 | -1978.372246 | 15.1 |
| **M3** | 1 | 0.840285 | -3956.749092 | 12.2 | -3956.748812 | 12.4 |
| **M4** | 1 | 0.837806 | -3956.727056 | 26.0 | -3956.726776 | 26.2 |
| **M5** | 1 | 0.840847 | -3956.774398 | -3.7 | -3956.774118 | -3.5 |
| **M6** | 1 | 0.839666 | -3956.749031 | 12.3 | -3956.748751 | 12.4 |
| **M7** | 1 | 0.841639 | -3956.768223 | 0.2 | -3956.767942 | 0.4 |
| **M8** | 1 | 0.840809 | -3956.756514 | 7.6 | -3956.756234 | 7.7 |
| **M9** | 1 | 0.842329 | -3956.750866 | 11.1 | -3956.750586 | 11.3 |
| **M10** | 1 | 0.841393 | -3956.756531 | 7.6 | -3956.756251 | 7.7 |
| **M11** | 1 | 0.843175 | -3956.753657 | 9.4 | -3956.753377 | 9.5 |
| **M12** | 1 | 0.845729 | -3956.761010 | 4.7 | -3956.760730 | 4.9 |
| **M13** | 1 | 0.844168 | -3956.759646 | 5.6 | -3956.759365 | 5.8 |
| **M14** | 1 | 0.844976 | -3956.769100 | -0.3 | -3956.768820 | -0.2 |
| **M15** | 1 | 0.847407 | -3956.751975 | 10.4 | -3956.751695 | 10.6 |
| **M16** | 1 | 0.846193 | -3956.778221 | -6.1 | -3956.777941 | -5.9 |
| **M17** | 1 | 0.847272 | -3956.764240 | 2.7 | -3956.763960 | 2.9 |
| **M18** | 1 | 0.846072 | -3956.769584 | -0.6 | -3956.769304 | -0.5 |
| **M19** | 1 | 0.845289 | -3956.766949 | 1.0 | -3956.766669 | 1.2 |
| **M20** | 1 | 0.794186 | -3878.281330 | 5.0 | -3878.281050 | 5.4 |
| **M21** | 1 | 0.791629 | -3878.274401 | 9.4 | -3878.274121 | 9.7 |
| **M23** | 3 | 0.842978 | -3956.785285 | -10.5 | -3956.785005 | -10.3 |
| **M24** | 3 | 0.838258 | -3956.691330 | 48.5 | -3956.691050 | 48.6 |
| **M25** | 3 | 0.841034 | -3956.766485 | 1.3 | -3956.766204 | 1.5 |
| **M26** | 3 | 0.843467 | -3956.766672 | 1.2 | -3956.766392 | 1.4 |
| **M27** | 3 | 0.842811 | -3956.759736 | 5.5 | -3956.759456 | 5.7 |
| **M28** | 3 | 0.844677 | -3956.768176 | 0.2 | -3956.767896 | 0.4 |
| **M29** | 3 | 0.844269 | -3956.757819 | 6.7 | -3956.757539 | 6.9 |
| **M30** | 3 | 0.844182 | -3956.765891 | 1.7 | -3956.765611 | 1.9 |
| **M32** | 3 | 0.844526 | -3956.767132 | 0.9 | -3956.766852 | 1.1 |
| **M33** | 3 | 0.844063 | -3956.769939 | -0.9 | -3956.769659 | -0.7 |
| **M34** | 3 | 0.788207 | -3878.265508 | 15.0 | -3878.265228 | 15.3 |
| **M35** | 5 | 0.788922 | -3878.266684 | 14.2 | -3878.266404 | 14.6 |

[a] Relative energies are calculated with respect to **M1**.

**Table S5.** Estimated Free Energy Components (a.u.) and Relative Free Energies (kcal/mol) of Minimum Energy Crossing Points (MECPs) of Complexes Bearing an $H_2$IMes Ligand.

| Model ID | $\overline{\Delta G}^{T=263\,K}_{wB97XD,qh}$ [a.u.] | $G^{[1\,mM]}$ [a.u.] | min $\Delta G^{[1\,mM]}$ [kcal/mol][a,b] | max $\Delta G^{[1\,mM]}$ [kcal/mol][a,c] | $G^{[1.4\,mM]}$ [a.u.] | min $\Delta G^{[1.4\,mM]}$ [kcal/mol][a,b] | max $\Delta G^{[1.4\,mM]}$ [kcal/mol][a,c] |
|---|---|---|---|---|---|---|---|
| **M22** | 0.844353648 | -3956.759179 | 6.9 | 10.9 | -3956.758899 | 7.1 | 11.1 |
| **M31** | 0.845126959 | -3956.759363 | 6.8 | 10.8 | -3956.759083 | 6.9 | 10.9 |

[a] Relative energies are calculated with respect to **M1**. [b]: Calculated using $\Delta G_{pXover} = 1$. [c]: Calculated using $\Delta G_{pXover} = 5$.

**Table S6.** Free Energies (a.u.) and Relative Free Energies (kcal/mol) of Species Bearing Ligand **C1$^{Ph}$**.

| Model ID | Spin multiplicity | $\Delta G^{T=263\,K}_{wB97XD,qh}$ [a.u.] | $G^{[1\,mM]}$ [a.u.] | $\Delta G^{[1\,mM]}$ [kcal/mol][a] | $G^{[61\,\mu M]}$ [a.u.] | $\Delta G^{[61\,\mu M]}$ [kcal/mol][a] |
|---|---|---|---|---|---|---|
| **M75** | 1 | 0.541081 | -2249.575410 | 0.0 | -2249.577739 | 0.0 |
| **M76** | 1 | 0.540887 | -2249.572938 | 1.6 | -2249.575267 | 1.6 |
| **M77** | 1 | 0.452479 | -2001.536374 | 5.4 | -2001.538703 | 3.9 |
| **M78** | 1 | 0.452422 | -2001.535894 | 5.7 | -2001.538223 | 4.2 |

[a] Relative energies are calculated with respect to **M75**.

**Table S7.** Free Energies (a.u.) and Relative Free Energies (kcal/mol) of Species Bearing Ligand **C1$^{Ph}$($I_2$)**.

| Model ID | Spin multiplicity | $\Delta G^{T=263\,K}_{wB97XD,qh}$ [a.u.] | $G^{[1\,mM]}$ [a.u.] | $\Delta G^{[1\,mM]}$ [kcal/mol][a] | $G^{[586\,\mu M]}$ [a.u.] | $\Delta G^{[586\,\mu M]}$ [kcal/mol][a] |
|---|---|---|---|---|---|---|
| **M87** | 1 | 0.538352 | -1920.928934 | 0.0 | -1920.929379 | 0.0 |
| **M88** | 1 | 0.539464 | -1920.923557 | 3.4 | -1920.924003 | 3.4 |
| **M89** | 1 | 0.449530 | -1672.891054 | 4.7 | -1672.891499 | 4.4 |
| **M90** | 1 | 0.449227 | -1672.887831 | 6.7 | -1672.888276 | 6.4 |

[a] Relative energies are calculated with respect to **M87**.

**Table S8.** Free Energies (a.u.) and Relative Free Energies (kcal/mol) of Species Bearing Ligand **C2**$^{Me}$.

| Model ID | Spin multiplicity | $\Delta G_{WB97XD,qh}^{T=263\,K}$ [a.u.] | $G^{[1\,mM]}$ [a.u.] | $\Delta G^{[1\,mM]}$ [kcal/mol][a] | $G^{[10\,\mu M]}$ [a.u.] | $\Delta G^{[10\,\mu M]}$ [kcal/mol][a] |
|---|---|---|---|---|---|---|
| **M79** | 1 | 0.488464 | -2058.039357 | 0.0 | -2058.043193 | 0.0 |
| **M80** | 1 | 0.488460 | -2058.036992 | 1.5 | -2058.040827 | 1.5 |
| **M81** | 1 | 0.399787 | -1810.008293 | 0.4 | -1810.012129 | -2.0 |
| **M82** | 1 | 0.400387 | -1810.002984 | 3.7 | -1810.006819 | 1.3 |

[a] Relative energies are calculated with respect to **M79.**

**Table S9.** Free Energies (a.u.) and Relative Free Energies (kcal/mol) of Species Bearing Ligand **C1**$^{Me}$.

| Model ID | Spin multiplicity | $\Delta G_{WB97XD,qh}^{T=263\,K}$ [a.u.] | $G^{[1\,mM]}$ [a.u.] | $\Delta G^{[1\,mM]}$ [kcal/mol][a] | $G^{[10\,\mu M]}$ [a.u.] | $\Delta G^{[10\,\mu M]}$ [kcal/mol][a] |
|---|---|---|---|---|---|---|
| **M83** | 1 | 0.488604 | -2058.042235 | 0.0 | -2058.046071 | 0.0 |
| **M84** | 1 | 0.488743 | -2058.039066 | 2.0 | -2058.042901 | 2.0 |
| **M85** | 1 | 0.400675 | -1810.007392 | 2.8 | -1810.011228 | 0.4 |
| **M86** | 1 | 0.400887 | -1810.000609 | 7.0 | -1810.004445 | 4.6 |

[a] Relative energies are calculated with respect to **M83**.

**Table S10.** Free Energies (a.u.) and Relative Free Energies (kcal/mol) of Species Bearing Ligand **C3**$^{\mathbf{Ph}}$.

| Model ID | Spin multiplicity | $\Delta G^{T=263\,K}_{wB97XD,qh}$ [a.u.] | $G^{[1\,mM]}$ [a.u.] | $\Delta G^{[1\,mM]}$ [kcal/mol][a] | $G^{[27\,\mu M]}$ [a.u.] | $\Delta G^{[27\,\mu M]}$ [kcal/mol][a] |
|---|---|---|---|---|---|---|
| **M36** | 1 | 0.511926 | -2210.336449 | 0.0 | -2210.339457 | 0.0 |
| **M37** | 1 | 0.511858 | -2210.335789 | 0.4 | -2210.338797 | 0.4 |
| **M38** | 1 | 0.423786 | -1962.296917 | 5.7 | -1962.299925 | 3.8 |
| 2 x **M38** | 1 | 0.423786 | -1962.296917 | 11.4 | -1962.299925 | 7.7 |
| **M39** | 1 | 0.421810 | -1962.296105 | 6.2 | -1962.299113 | 4.3 |
| 2 x **M39** | 1 | 0.421810 | -1962.296105 | 12.5 | -1962.299113 | 8.7 |
| **M40** | 1 | 0.422686 | -1962.280632 | 15.9 | -1962.283640 | 14.0 |
| **M41** | 1 | 0.871500 | -3924.591816 | 12.7 | -3924.594824 | 10.8 |
| **M42** | 1 | 0.872383 | -3924.595678 | 10.3 | -3924.598686 | 8.4 |
| **M43** | 1 | 0.874039 | -3924.598378 | 8.6 | -3924.601386 | 6.7 |
| **M44** | 1 | 0.871334 | -3924.592334 | 12.4 | -3924.595343 | 10.5 |
| **M45** | 1 | 0.873821 | -3924.606152 | 3.7 | -3924.609160 | 1.8 |
| **M46** | 1 | 0.871764 | -3924.595446 | 10.4 | -3924.598454 | 8.5 |
| **M47** | 1 | 0.873641 | -3924.605618 | 4.0 | -3924.608626 | 2.2 |
| **M48** | 1 | 0.873897 | -3924.600710 | 7.1 | -3924.603718 | 5.2 |
| **M49** | 1 | 0.872348 | -3924.615050 | -1.9 | -3924.618058 | -3.8 |
| **M50** | 1 | 0.873504 | -3924.609674 | 1.5 | -3924.612682 | -0.4 |
| **M51** | 1 | 0.873502 | -3924.610984 | 0.7 | -3924.613992 | -1.2 |
| **M52** | 1 | 0.872516 | -3924.608541 | 2.2 | -3924.611549 | 0.3 |
| **M53** | 1 | 0.872762 | -3924.608402 | 2.3 | -3924.611410 | 0.4 |
| **M54** | 1 | 0.874516 | -3924.605013 | 4.4 | -3924.608021 | 2.5 |
| **M55** | 1 | 0.872099 | -3924.611565 | 0.3 | -3924.614573 | -1.6 |
| **M56** | 1 | 0.872536 | -3924.609323 | 1.7 | -3924.612332 | -0.2 |
| **M57** | 1 | 0.872809 | -3924.608495 | 2.2 | -3924.611503 | 0.3 |
| **M58** | 1 | 0.872302 | -3924.608797 | 2.0 | -3924.611805 | 0.2 |
| **M59** | 1 | 0.875099 | -3924.600101 | 7.5 | -3924.603109 | 5.6 |
| **M60** | 1 | 0.874598 | -3924.598879 | 8.3 | -3924.601887 | 6.4 |
| **M61** | 1 | 0.875409 | -3924.593438 | 11.7 | -3924.596446 | 9.8 |
| **M62** | 1 | 0.874884 | -3924.596384 | 9.8 | -3924.599393 | 7.9 |
| **M63** | 1 | 0.875529 | -3924.589374 | 14.2 | -3924.592382 | 12.3 |
| **M64** | 1 | 0.874382 | -3924.587563 | 15.4 | -3924.590571 | 13.5 |
| **M65** | 1 | 0.875408 | -3924.593816 | 11.4 | -3924.596824 | 9.6 |
| **M66** | 1 | 0.874597 | -3924.593204 | 11.8 | -3924.596212 | 9.9 |
| **M67** | 1 | 0.874562 | -3924.598954 | 8.2 | -3924.601962 | 6.3 |
| **M68** | 1 | 0.875946 | -3924.586420 | 16.1 | -3924.589428 | 14.2 |
| **M69** | 1 | 0.874905 | -3924.597655 | 9.0 | -3924.600663 | 7.1 |
| **M70** | 1 | 0.875046 | -3924.598285 | 8.6 | -3924.601293 | 6.8 |
| **M72** | 1 | 0.875422 | -3924.588378 | 14.9 | -3924.591386 | 13.0 |
| **M73** | 1 | 0.874609 | -3924.591562 | 12.9 | -3924.594570 | 11.0 |
| **M74** | 1 | 0.874750 | -3924.596715 | 9.6 | -3924.599723 | 7.7 |

[a] Relative energies are calculated with respect to **M36**.

## S6. References


(1) Gawin, R.; Kozakiewicz, A.; Guńka, P. A.; Dąbrowski, P.; Skowerski, K., Bis(Cyclic Alkyl Amino Carbene) Ruthenium Complexes: A Versatile, Highly Efficient Tool for Olefin Metathesis. *Angew. Chem., Int. Ed.* **2017**, *56*, 981–986.

(2) Romero, P. E.; Piers, W. E.; McDonald, R., Rapidly initiating ruthenium olefin-metathesis catalysts Angew. Chemie 2004 116, 6287-6291. *Angew. Chem., Int. Ed.* **2004**, *43*, 6161–6165.

(3) Marx, V. M.; Sullivan, A. H.; Melaimi, M.; Virgil, S. C.; Keitz, B. K.; Weinberger, D. S.; Bertrand, G.; Grubbs, R. H., Cyclic Alkyl Amino Carbene (CAAC) Ruthenium Complexes as Remarkably Active Catalysts for Ethenolysis. *Angew. Chem., Int. Ed.* **2015**, *54*, 1919–1923.

(4) Tracz, A.; Matczak, M.; Urbaniak, K.; Skowerski, K., Nitro-Grela-Type Complexes Containing Iodides – Robust and Selective Catalysts for Olefin Metathesis Under Challenging Conditions. *Beilstein J. Org. Chem.* **2015**, *11*, 1823–1832.

(5) Blanco, C.; Nascimento, D. L.; Fogg, D. E., Routes to High-Performing Ruthenium-Iodide Catalysts for Olefin Metathesis: Phosphine Lability Is Key to Efficient Halide Exchange. *Organometallics* **2021**, ASAP article.

(6) Keitz, B. K.; Grubbs, R. H., Probing the Origin of Degenerate Metathesis Selectivity via Characterization and Dynamics of Ruthenacyclobutanes Containing Variable NHCs. *J. Am. Chem. Soc.* **2011**, *133*, 16277–16284.

(7) Nascimento, D. L.; Fogg, D. E., Origin of the Breakthrough Productivity of Ruthenium-CAAC Catalysts in Olefin Metathesis (CAAC = Cyclic Alkyl Amino Carbene). *J. Am. Chem. Soc.* **2019**, *141*, 19236–19240.

(8) Bailey, G. A.; Fogg, D. E., Confronting Neutrality: Maximizing Success in the Analysis of Transition-Metal Catalysts by MALDI Mass Spectrometry. *ACS Catal.* **2016**, *6*, 4962–4971.

(9) Bailey, G. A.; Foscato, M.; Higman, C. S.; Day, C. S.; Jensen, V. R.; Fogg, D. E., Bimolecular Coupling as a Vector for Decomposition of Fast-Initiating Olefin Metathesis Catalysts. *J. Am. Chem. Soc.* **2018**, *140*, 6931–6944.

(10) Nascimento, D. L.; Gawin, A.; Gawin, R.; Guńka, P. A.; Zachara, J.; Skowerski, K.; Fogg, D. E., Integrating Activity with Accessibility in Olefin Metathesis: An Unprecedentedly Reactive Ruthenium-Indenylidene Catalyst Bearing a Cyclic Alkyl Amino Carbene. *J. Am. Chem. Soc.* **2019**, *141*, 10626–10631.

(11) Amoroso, D.; Yap, G. P. A.; Fogg, D. E., Deactivation of Ruthenium Metathesis Catalysts via Facile Formation of Face-Bridged Dimers. *Organometallics* **2002**, *21*, 3335–3343.

(12) Amoroso, D.; Snelgrove, J. L.; Conrad, J. C.; Drouin, S. D.; Yap, G. P. A.; Fogg, D. E., An Attractive Route to Olefin Metathesis Catalysts: Facile Synthesis of a Ruthenium Alkylidene Complex Containing Labile Phosphane Donors. *Adv. Synth. Catal.* **2002**, *344*, 757–763.

(13) Higman, C. S.; Lanterna, A. E.; Marin, M. L.; Scaiano, J. C.; Fogg, D. E., Catalyst Decomposition During Olefin Metathesis Yields Isomerization-Active Ru Nanoparticles. *ChemCatChem* **2016**, *8*, 2446–2449.

(14) Connelly, N. G.; Damhus, T.; Hartshorn, R. M.; Hutton, A. T., *Nomenclature of Inorganic Chemistry – IUPAC Recommendations*. Royal Society of Chemistry: Cambridge, U.K., 2005.

(15)*Marvin 18.24.0*, 2018, ChemAxon Ltd. (http://www.chemaxon.com/).

(16) Pettersen, E. F.; Goddard, T. D.; Huang, C. C.; Couch, G. S.; Greenblatt, D. M.; Meng, E. C.; Ferrin, T. E., UCSF Chimera—A visualization system for exploratory research and analysis. *J. Comput. Chem.* **2004**, *25*, 1605–1612.

(17) Schaftenaar, G.; Noordik, J. H., Molden: a pre- and post-processing program for molecular and electronic structures. *J. Comput.-Aided Mol. Des.* **2000**, *14*, 123–134.

(18) Halgren, T. A., Merck molecular force field. I. Basis, form, scope, parameterization, and performance of MMFF94. *J. Comput. Chem.* **1996**, *17*, 490–519.

(19) Stewart, J. J. P., Optimization of parameters for semiempirical methods V: Modification of NDDO approximations and application to 70 elements. *J. Mol. Model.* **2007**, *13*, 1173–1213.

(20) Frisch, M. J.; Trucks, G. W.; Schlegel, H. B.; Scuseria, G. E.; Robb, M. A.; Cheeseman, J. R.; Scalmani, G.; Barone, V.; Mennucci, B.; Petersson, G. A.; Nakatsuji, H.; Caricato, M.; Li, X.; Hratchian, H. P.; Izmaylov, A. F.; Bloino, J.; Zheng, G.; Sonnenberg, J. L.; Hada, M.; Ehara, M.; Toyota, K.; Fukuda, R.; Hasegawa, J.; Ishida, M.; Nakajima, T.; Honda, Y.; Kitao, O.; Nakai, H.; Vreven, T.; Montgomery, J. A.; Peralta, J. E.; Ogliaro, F.; Bearpark, M.; Heyd, J. J.; Brothers, E.; Kudin, K. N.; Staroverov, V. N.; Keith, T.; Kobayashi, R.; Normand, J.; Raghavachari, K.; Rendell, A.; Burant, J. C.; Iyengar, S. S.; Tomasi, J.; Cossi, M.; Rega, N.; Millam, J. M.; Klene, M.; Knox, J. E.; Cross, J. B.; Bakken, V.; Adamo, C.; Jaramillo, J.; Gomperts, R.; Stratmann, R. E.; Yazyev, O.; Austin, A. J.; Cammi, R.; Pomelli, C.; Ochterski, J. W.; Martin, R. L.; Morokuma, K.; Zakrzewski, V. G.; Voth, G. A.; Salvador, P.; Dannenberg, J. J.; Dapprich, S.; Daniels, A. D.; Farkas, O.; Foresman, J. B.; Ortiz, J. V.; Cioslowski, J.; Fox, D. J., Gaussian 09, Revision D.01, Gaussian, Inc., Wallingford CT. 2013.

(21) Frisch, M. J.; Trucks, G. W.; Schlegel, H. B.; Scuseria, G. E.; Robb, M. A.; Cheeseman, J. R.; Scalmani, G.; Barone, V.; Petersson, G. A.; Nakatsuji, H.; Li, X.; Caricato, M.; Marenich, A. V.; Bloino, J.; Janesko, B. G.; Gomperts, R.; Mennucci, B.; Hratchian, H. P.; Ortiz, J. V.; Izmaylov, A. F.; Sonnenberg, J. L.; Williams; Ding, F.; Lipparini, F.; Egidi, F.; Goings, J.; Peng, B.; Petrone, A.; Henderson, T.; Ranasinghe, D.; Zakrzewski, V. G.; Gao, J.; Rega, N.; Zheng, G.; Liang, W.; Hada, M.; Ehara, M.; Toyota, K.; Fukuda, R.; Hasegawa, J.; Ishida, M.; Nakajima, T.; Honda, Y.; Kitao, O.; Nakai, H.; Vreven, T.; Throssell, K.; Montgomery Jr., J. A.; Peralta, J. E.; Ogliaro, F.; Bearpark, M. J.; Heyd, J. J.; Brothers, E. N.; Kudin, K. N.; Staroverov, V. N.; Keith, T. A.; Kobayashi, R.; Normand, J.; Raghavachari, K.; Rendell, A. P.; Burant, J. C.; Iyengar, S. S.; Tomasi, J.; Cossi, M.; Millam, J. M.; Klene, M.; Adamo, C.; Cammi, R.; Ochterski, J. W.; Martin, R. L.; Morokuma, K.; Farkas, O.; Foresman, J. B.; Fox, D. J., Gaussian 16 Rev. C.01: Wallingford, CT, 2016.

(22) Chai, J.-D.; Head-Gordon, M., Long-range corrected hybrid density functionals with damped atom-atom dispersion corrections. *Phys. Chem. Chem. Phys.* **2008**, *10*, 6615–6620.

(23) Minenkov, Y.; Singstad, Å.; Occhipinti, G.; Jensen, V. R., The Accuracy of DFT-Optimized Geometries of Functional Transition Metal Compounds: A Validation Study of Catalysts for Olefin Metathesis and Other Reactions in The Homogeneous Phase. *Dalton Trans.* **2012**, *41*, 5526–5541.

(24) Dunning, T. H., Gaussian Basis Sets for Use in Correlated Molecular Calculations. I. The Atoms Boron Through Neon and Hydrogen. *J. Chem. Phys.* **1989**, *90*, 1007–1023.

(25) Woon, D. E.; Dunning, T. H., Gaussian Basis Sets for Use in Correlated Molecular Calculations. III. The Atoms Aluminum through Argon. *J. Chem. Phys.* **1993**, *98*, 1358–1371.

(26) Schuchardt, K. L.; Didier, B. T.; Elsethagen, T.; Sun, L.; Gurumoorthi, V.; Chase, J.; Li, J.; Windus, T. L., Basis Set Exchange: A Community Database for Computational Sciences. *J. Chem. Inf. Model.* **2007**, *47*, 1045–1052.

(27) Feller, D., The Role of Databases in Support of Computational Chemistry Calculations. *J. Comput. Chem.* **1996**, *17*, 1571–1586.

(28) Peterson, K. A.; Figgen, D.; Dolg, M.; Stoll, H., Energy-consistent relativistic pseudopotentials and correlation consistent basis sets for the 4d elements Y–Pd. *J. Chem. Phys.* **2007**, *126*, 124101.

(29) http://www.tc.uni-koeln.de/PP/clickpse.en.html (accessed 18-03-2020).

(30) Harvey, J. N.; Aschi, M.; Schwarz, H.; Koch, W., The singlet and triplet states of phenyl cation. A hybrid approach for locating minimum energy crossing points between non-interacting potential energy surfaces. *Theor. Chem. Acc.* **1998**, *99*, 95–99.

(31) Gannon, K. L.; Blitz, M. A.; Liang, C.-H.; Pilling, M. J.; Seakins, P. W.; Glowacki, D. R.; Harvey, J. N., An experimental and theoretical investigation of the competition between chemical reaction and relaxation for the reactions of 1CH2 with acetylene and ethene: implications for the chemistry of the giant planets. *Faraday Discuss.* **2010**, *147*, 173–188.

(32) Álvarez-Moreno, M.; de Graaf, C.; López, N.; Maseras, F.; Poblet, J. M.; Bo, C., Managing the Computational Chemistry Big Data Problem: The ioChem-BD Platform. *J. Chem. Inf. Model.* **2015**, *55,* 95–103.

(33) Bo, C.; Maseras, F.; López, N., The role of computational results databases in accelerating the discovery of catalysts. *Nat. Catal.* **2018**, *1,* 809–810.

(34) Zhao, Y.; Truhlar, D. G., Computational characterization and modeling of buckyball tweezers: density functional study of concave–convex π⋯π interactions. *Phys. Chem. Chem. Phys.* **2008**, *10*, 2813–2818.

(35) Ribeiro, R. F.; Marenich, A. V.; Cramer, C. J.; Truhlar, D. G., Use of Solution-Phase Vibrational Frequencies in Continuum Models for the Free Energy of Solvation. *J. Phys. Chem. B* **2011**, *115*, 14556–14562.

(36) Perdew, J. P.; Burke, K.; Ernzerhof, M., Generalized Gradient Approximation Made Simple. *Phys. Rev. Lett.* **1996**, *77*, 3865–3868.

(37) Perdew, J.; Burke, K.; Ernzerhof, M., Generalized Gradient Approximation Made Simple [Phys. Rev. Lett. 77, 3865 (1996)]. *Phys. Rev. Lett.* **1997**, *78*, 1396–1396.

(38) Grimme, S.; Ehrlich, S.; Goerigk, L., Effect of the damping function in dispersion corrected density functional theory. *J. Comput. Chem.* **2011**, *32*, 1456–1465.

(39) Minenkov, Y.; Occhipinti, G.; Jensen, V. R., Complete Reaction Pathway of Ruthenium-Catalyzed Olefin Metathesis of Ethyl Vinyl Ether: Kinetics and Mechanistic Insight from DFT. *Organometallics* **2013**, *32*, 2099−2111.

(40) Smith, D. G. A.; Burns, L. A.; Patkowski, K.; Sherrill, C. D., Revised Damping Parameters for the D3 Dispersion Correction to Density Functional Theory. *J. Phys. Chem. Lett.* **2016**, *7*, 2197–2203.

(41) Cossi, M.; Scalmani, G.; Rega, N.; Barone, V., New developments in the polarizable continuum model for quantum mechanical and classical calculations on molecules in solution. *J. Chem. Phys.* **2002**, *117,* 43–54.

(42) Tomasi, J.; Mennucci, B.; Cammi, R., Quantum Mechanical Continuum Solvation Models. *Chem. Rev.* **2005**, *105*, 2999–3093.

(43) Scalmani, G.; Frisch, M. J., Continuous Surface Charge Polarizable Continuum Models of Solvation. I. General Formalism. *J. Chem. Phys.* **2010**, *132*, 114110.

(44) Kendall, R. A.; Dunning, T. H.; Harrison, R. J., Electron affinities of the first-row atoms revisited. Systematic basis sets and wave functions. *J. Chem. Phys.* **1992**, *96*, 6796–6806.

(45) Fernández-Ramos, A.; Miller, J. A.; Klippenstein, S. J.; Truhlar, D. G., Modeling the Kinetics of Bimolecular Reactions. *Chem. Rev.* **2006**, *106*, 4518-4584.

(46) McMullin, C. L.; Jover, J.; Harvey, J. N.; Fey, N., Accurate modelling of Pd(0) + PhX oxidative addition kinetics. *Dalton Trans.* **2010**, *39,* 10833-10836.

(47) Schiwek, C.; Meiners, J.; Förster, M.; Würtele, C.; Diefenbach, M.; Holthausen, M. C.; Schneider, S., Oxygen Reduction with a Bifunctional Iridium Dihydride Complex. *Angew. Chem., Int. Ed.* **2015**, *54,* 15271–15275.

(48) Harvey, J. N., Understanding the kinetics of spin-forbidden chemical reactions. *Phys. Chem. Chem. Phys.* **2007**, *9,* 331–343.

(49) Glendening, E. D.; Badenhoop, J. K.; Reed, A. E.; Carpenter, J. E.; Bohmann, J. A.; Morales, C. M.; Karafiloglou, P.; Landis, C. R.; Weinhold, F., NBO 7.0.; Theoretical Chemistry Institute, University of Wisconsin, Madison, WI 2018.

(50) Poater, A.; Cosenza, B.; Correa, A.; Giudice, S.; Ragone, F.; Scarano, V.; Cavallo, L., SambVca: A Web Application for the Calculation of the Buried Volume of N-Heterocyclic Carbene Ligands. *Eur. J. Inorg. Chem.* **2009**, 1759–1766.

(51) Bondi, A., Van Der Waals Volumes and Radii. *J. Phys. Chem.* **1964**, *68*, 441–451.
